# Evidence linking COVID-19 and the health/well-being of children and adolescents: an umbrella review

**DOI:** 10.1186/s12916-024-03334-x

**Published:** 2024-03-13

**Authors:** Chengchen Duan, Liu Liu, Tianyi Wang, Guanru Wang, Zhishen Jiang, Honglin Li, Gaowei Zhang, Li Ye, Chunjie Li, Yubin Cao

**Affiliations:** 1 State Key Laboratory of Oral Diseases & National Center for Stomatology & National Clinical Research Center for Oral Diseases, West China Hospital of Stomatology, Sichuan University, No.14, 3rd Section of Ren Min Nan Rd., Chengdu, 610041 China; 2 Department of Conservative Dentistry and Endodontics, West China Hospital of Stomatology, Sichuan University, Chengdu, China; 3 Department of Head and Neck Oncology, West China Hospital of Stomatology, Sichuan University, Chengdu, China; 4 Department of Oral and Maxillofacial Surgery, West China Hospital of Stomatology, Sichuan University, Chengdu, China; 5 Department of Evidence-Based Stomatology, West China Hospital of Stomatology, Sichuan University, Chengdu, China

**Keywords:** COVID-19, Children and adolescents, Comorbidity, Mental health, Umbrella review

## Abstract

**Background:**

Experiences during childhood and adolescence have enduring impacts on physical and mental well-being, overall quality of life, and socioeconomic status throughout one’s lifetime. This underscores the importance of prioritizing the health of children and adolescents to establish an impactful healthcare system that benefits both individuals and society. It is crucial for healthcare providers and policymakers to examine the relationship between COVID-19 and the health of children and adolescents, as this understanding will guide the creation of interventions and policies for the long-term management of the virus.

**Methods:**

In this umbrella review (PROSPERO ID: CRD42023401106), systematic reviews were identified from the Cochrane Database of Systematic Reviews; EMBASE (OvidSP); and MEDLINE (OvidSP) from December 2019 to February 2023. Pairwise and single-arm meta-analyses were extracted from the included systematic reviews. The methodological quality appraisal was completed using the AMSTAR-2 tool. Single-arm meta-analyses were re-presented under six domains associated with COVID-19 condition. Pairwise meta-analyses were classified into five domains according to the evidence classification criteria. Rosenberg’s FSN was calculated for both binary and continuous measures.

**Results:**

We identified 1551 single-arm and 301 pairwise meta-analyses from 124 systematic reviews that met our predefined criteria for inclusion. The focus of the meta-analytical evidence was predominantly on the physical outcomes of COVID-19, encompassing both single-arm and pairwise study designs. However, the quality of evidence and methodological rigor were suboptimal. Based on the evidence gathered from single-arm meta-analyses, we constructed an illustrative representation of the disease severity, clinical manifestations, laboratory and radiological findings, treatments, and outcomes from 2020 to 2022. Additionally, we discovered 17 instances of strong or highly suggestive pairwise meta-analytical evidence concerning long-COVID, pediatric comorbidity, COVID-19 vaccines, mental health, and depression.

**Conclusions:**

The findings of our study advocate for the implementation of surveillance systems to track health consequences associated with COVID-19 and the establishment of multidisciplinary collaborative rehabilitation programs for affected younger populations. In future research endeavors, it is important to prioritize the investigation of non-physical outcomes to bridge the gap between research findings and clinical application in this field.

**Supplementary Information:**

The online version contains supplementary material available at 10.1186/s12916-024-03334-x.

## Background

The coronavirus disease 2019 (COVID-19), caused by severe acute respiratory syndrome coronavirus 2 (SARS-CoV-2), has been spreading globally for more than 3 years [1, 2]. As of April 20, 2023, there have been over 765 million confirmed cases and over 6.9 million deaths reported worldwide [3]. COVID-19 has had varied effects on the health of children and adolescents, both directly and indirectly. COVID-19 infection can cause symptoms and impact the physical health of young people, affecting multiple organ systems directly [4–6]. Additionally, the policies implemented during the pandemic, as well as the preventive measures aimed at reducing the direct impact of COVID-19, often give rise to indirect consequences for children and adolescents. These indirect effects of COVID-19 have a disruptive impact on routine healthcare services and social interactions, which can further exacerbate mental and cognitive health challenges and worsen existing health disparities among this vulnerable population [7, 8]. Child and adolescent health refer to the physical, mental/cognitive, quality of life, and social well-being, of individuals from newborns until the age of 19. Experiences during childhood and adolescence have enduring impacts on physical and mental health, quality of life, and socioeconomic status over the lifespan [9]. Consequently, exploring the subsequent effects of COVID-19 on the health of children and adolescents has the potential to influence the future provision and design of comprehensive services for those affected by COVID‐19. By gaining insights into an individual’s informational, spiritual, psychological, social, and physical requirements during follow-up phases, personalized services can be developed to enhance the survivor experience. This endeavor plays a vital role in establishing a resilient and prosperous healthcare system that benefits both individuals and society.

Currently, the World Health Organization (WHO) has declared that the global health emergency caused by COVID-19 has ended. This highlights the need to transition from an emergency response to the long-term management of COVID-19 and other infectious diseases [10, 11]. Even after the emergency phase concludes, the ongoing transmission and emergence of new COVID-19 variants, as well as the remaining unvaccinated younger individuals and the significant global impact on health inequity, societal consequences, and economic repercussions, collectively emphasize the importance of continually assessing the available evidence on the correlation between COVID-19 and the health of children and adolescents. This assessment will inform stakeholders, including patients, healthcare providers, and policymakers, to mitigate conflicting effects and prioritize resources, interventions, and policies.

During the first year of the pandemic, a study analyzed all 6338 pediatric emergency admissions in England related to COVID-19 and found that adolescents have a higher likelihood of being hospitalized due to COVID-19 compared to younger children [12]. Surveys conducted among 13,002 American and 11,681 Chinese adolescents showed a similar 1-year prevalence of clinically significant depressive and anxiety symptoms during the COVID-19 pandemic [13, 14]. These nationally representative studies, conducted with large sample sizes, are commonly regarded as robust evidence to establish a link between COVID-19 and child and adolescent health across diverse domains and time periods. However, the changing public health policies of COVID-19 and the emergence of new viral strains could introduce complexities and inconsistencies in the overall evidence [15, 16]. In addition, many primary studies examining the relationship between COVID-19 and the health of children and adolescents used convenience sampling, including cross-sectional and observational designs that lacked control or comparison groups. The meta-analytical estimates from these studies may not accurately represent the true effects of the disease, as they are prone to biases such as measurement errors, poor control of confounders, biased participant selection, and data publication issues, ultimately weakening the strength of the aggregated scientific evidence [17]. The emergence of meta-analytical evidence through rapid reviews, compared to formal systematic reviews, further complicates this issue due to inadequate reporting of evidence, limited literature search, and increased publication bias [18].

The umbrella review, which involves quantifying systematic reviews and meta-analyses, provides a comprehensive assessment that captures the most extensive and high-quality medical evidence available [19]. By utilizing umbrella reviews, studies have reported evidence on the characteristic features of COVID-19 in children and adolescents during the initial phase of the pandemic [20], as well as the epidemiological impact and associations with mental health problems among this demographic [21, 22]. Although these studies to some extent synthesized evidence on various factors influencing pediatric health outcomes during the COVID-19 pandemic, they fell short of providing a comprehensive perspective on the diverse array of health and well-being outcomes among children and adolescents. Moreover, the conclusions drawn from these umbrella reviews lack an assessment based on evidence grading criteria, neglecting the systematic grading of the evidence obtained from studies. This oversight regarding the overall strength of evidence could potentially limit the credibility of the conclusions presented in umbrella reviews. Therefore, this study aims to conduct an umbrella review to evaluate the strength of meta-analytic estimates and summarize the current evidence linking direct and indirect impacts of COVID-19 to the health/well-being of children and adolescents. Furthermore, we intend to explore the potential impact of future research on the conclusions drawn from existing significant meta-analyses.

## Methods

### Protocol and reporting

The protocol of this umbrella review was prospectively defined and registered on the PROSPERO [23] website (ID: CRD42023401106) (https://www.crd.york.ac.uk/PROSPERO/). The differences between registered protocol and review were provided in Additional file 1. This review is reported according to the Preferred Reporting Items for Systematic Reviews and Meta‐Analyses (PRISMA) 2020 statement guidelines and a PRISMA checklist is included (Additional file 2) [24].

### Study search and selection

We performed a comprehensive systematic literature search without any restrictions on the date or language of publication. Three key electronic databases including the Cochrane Database of Systematic Reviews (CDSR) via The Cochrane Library; EMBASE (OvidSP) and MEDLINE (OvidSP) were searched from December 2019 to February 2023. Moreover, the Google Scholar and WHO database of publications on COVID-19 and reference lists of included studies were also searched manually to identify reports of additional studies. We merged keywords and subject headings appropriately for each database using the following search terms: (COVID-19 [MeSH] OR 2019-nCoV.m.p. OR SARS-CoV-2.m.p. OR novel coronavirus pneumonia.m.p.) AND (pediatrics [MeSH] OR pediatrics.m.p. OR neonate.m.p. OR children.m.p. OR adolescence.m.p. OR teenagers.m.p.) AND (meta-analysis [MeSH] OR systematic review.m.p.) (Additional file 3). Two independent authors (C.D. and T.W.) carried out the electronic database search and decided the final inclusion according to the following criteria: (1) systematic reviews with meta-analysis; (2) results from children and adolescents between 0 and 19 years old; (3) observing COVID-19 as the exposure. Full texts were obtained and independently assessed for eligibility if certain studies seemed to have any potentiality for inclusion if they could not be judged completely by titles and abstracts. Any disagreements were settled by consulting a third review author (L.L.). Observational studies, intervention studies, other types of reviews (descriptive, scoping), program evaluations, animal studies, conference abstracts, and letters/comments were excluded from the review.

### Data extraction

Two independent reviewers (C.D. and L.L.) screened the titles and abstracts, assigning unique identification numbers to all the included articles. Two authors (C.D. and T.W.) independently extracted the necessary data from each eligible review through a pre-designed extraction table and resolved any disagreements by discussion with a third reviewer (L.L.). Pooled estimates, including prevalence, odds ratio (OR), relative risk (RR), hazard ratio (HR), and standard mean difference (SMD), were extracted from each systematic review for all eligible health and well-being outcomes. The pre-designed extraction table included study identification (authors, year, and origin country), number of studies and participants included in the meta-analysis, outcome domain (physical, psychological/cognitive, quality of life, social, and health system), direct or indirect impact(s), COVID-19 condition(s) being assessed, health and well-being condition(s) of children and adolescents being assessed, methodological quality tool used, effect size and 95% CI, heterogeneity (*I*^2^ statistic), and publication bias assessment. We defined the “direct effects” as the consequences that directly correlate with COVID-19 infection or transmission, specifically within outcome domains. On the other hand, the “indirect effects” encompassed the broader consequences that arise from the pandemic, as well as the public health or political regulations associated with it. Furthermore, two senior researchers (Z.J. and G.W.), specializing in epidemiology and disease prevention, critically reviewed both the methodology and the coding results.

To address missing data, we initially reached out to the authors of the meta-analytical studies in an attempt to acquire the missing information directly from the original research teams. If the pooled estimates were not provided and no response was received from the authors, the entire row of data was excluded without any further statistical transformations [25]. In cases where the statistical significance of the combined effect in the meta-analysis was determined using *Z*-tests but did not include reported *P*-values, we calculated the corresponding *P*-values based on the respective *Z*-value [26]. All data domains were verified to be free of missing values through the aforementioned processes.

### Methodological quality appraisal, evidence grading, and presentation

Methodological quality of the systematic review will be made using the A Measurement Tool to Assess Systematic Reviews 2 (AMSTAR-2) [27] tool by two examiners (C.D. and T.W.). In this sense, systematic reviews are categorized as: High (Zero or one non-critical weakness); Moderate (More than one non-critical weakness); Low (One critical flaw with or without non-critical weaknesses); and Critically Low (More than one critical flaw with or without non-critical weaknesses).

For pairwise meta-analytical evidence, statistical quality was assessed by applying the previously published evidence grading protocol [28, 29]. Significant associations shown in meta-analyses were categorized into four evidence levels: strong, highly suggestive, suggestive, and weak evidence. Strong evidence was considered if all the following criteria were met: > 1000 cases included in the meta-analysis; a *P*-value ≤ 10^−6^ of statistical significance in valid meta-analysis; heterogeneity (*I*^2^) below 50%; the null value was excluded by the 95% prediction interval; and no evidence of small study effects and excess significance bias. Highly suggestive evidence was set if meta-analyses with > 1000 cases; a random effects *P*-value ≤ 10^−6^, and the largest study in the meta-analysis was statistically significant. Suggestive evidence was defined if meta-analyses with > 1000 cases, random effects *P*-value ≤ 10^−3^ were categorized. If the latter conditions were not verified, the meta-analysis was classified as weak evidence. The classifications were subgrouped based on health domains, and the results were tabulated accordingly: the main focus of interest for this study encompassed the direct and indirect impacts associated with COVID-19, which included physical, psychological/cognitive, quality of life, and social impacts. The data was compared by considering the evidence grade and subgroups, and various methods such as counting and clustering were employed.

For single-arm meta-analytical evidence, six pre-defined COVID-19 condition domains were created: laboratory-confirmed COVID-19, COVID-19-associated MIS-C, newborns from COVID-19-diagnosed mothers, long-COVID, events caused by the COVID-19 vaccine, and health impacts during the pandemic. The domain of newborns from COVID-19 diagnosed mothers is exclusively limited to infants. The remaining domains, however, encompass children and adolescents aged 0–19 years old. Summarizations were conducted under each domain using all relevant meta-analytical evidence regardless of topic overlap to present and describe the current body of systematic review evidence on impacts of COVID-19 on children and adolescents. The most meta-analytical evidence was centered around laboratory-confirmed COVID-19. It is important to acknowledge that data on patient disease presentations collected during the early stages of the COVID-19 pandemic may differ significantly from those observed in later phases. Nonetheless, the effect summaries and publication years of original meta-analyses may not accurately capture these variations. To examine the changing trends in the occurrence rates of various symptoms during the pandemic, taking into account viral strain evolution and diverse health interventions, we conducted additional reanalysis of primary studies in this domain. Specifically, we screened and extracted relevant information from the primary studies included in each systematic review, including details of the authors, data collection years, outcome indicators, number of events, and sample sizes. Subsequently, we removed duplicated evidence and reanalyzed the primary data reported for at least 2 years within the meta-analytical evidence. To account for heterogeneity among the included studies, a random effects model was used to combine the primary data outcomes if *Q* < 0.05 or *I*^2^ > 50% [30]. Alternatively, a fixed-effects model was applied to pool outcomes if these criteria were not met [30, 31].

We adhered to the presentation guidelines outlined in the *Cochrane Handbook for Systematic Reviews of Interventions* to effectively present our current work [25]. To present pairwise meta-analytical evidence, we initially employed the “summarizing outcome data” [25] strategy. This strategy enabled us to systematically summarize the meta-analyzed data, along with the previously mentioned quality assessments, without considering any overlap in study topics. By doing so, we were able to provide a comprehensive overview of the current body of evidence from systematic reviews on the study topic. To present single-arm meta-analytical evidence, we also utilized the “summarizing outcome data” strategy to offer a comprehensive perspective on the available evidence. Additionally, we conducted a “reanalyzing outcome data” [25] specifically for primary studies of laboratory-confirmed COVID-19. This reanalysis aided in the elimination of duplicated primary studies and standardized the collection years of data. As a result, we achieved a more coherent and consistent presentation of disease severity, clinical manifestations, laboratory and radiological findings, treatments, and outcomes associated with laboratory-confirmed COVID-19 across different years.

### Calculations of FSN

The Rosenberg’s FSN is the number of missing studies averaging a *z*-value of zero that should be added to make the combined effect size statistically insignificant. For statistically significant meta-analytic evidence, Rosenberg’s FSN was calculated using the workbook “Meta-Essentials” [32] for binary and continuous measures.

### Data handling and processing

All the data were collected using MS Office 365. Data processing and statistical analysis were conducted in the R programming environment (version 4.1.0). The “bibliometrix” package (version 4.1.2) was employed to perform bibliometric analysis on the included studies, following its standard analyzing protocol [33]. The “meta” package (version 6.2.1) was used for statistical transformation and reanalysis of the primary studies from single-arm meta-analytical evidence [34]. To visualize the reanalyzed data, the “forestploter” package (version 1.1.2) was utilized (https://github.com/cran/forestploter). Data table formatting and cleaning were achieved through the utilization of the “tidyverse” (version 1.3.1) and “reshape2” packages (version 1.4.4) [35, 36].

## Results

### Selection and characteristics of the included meta-analyses

**Fig. 1:**
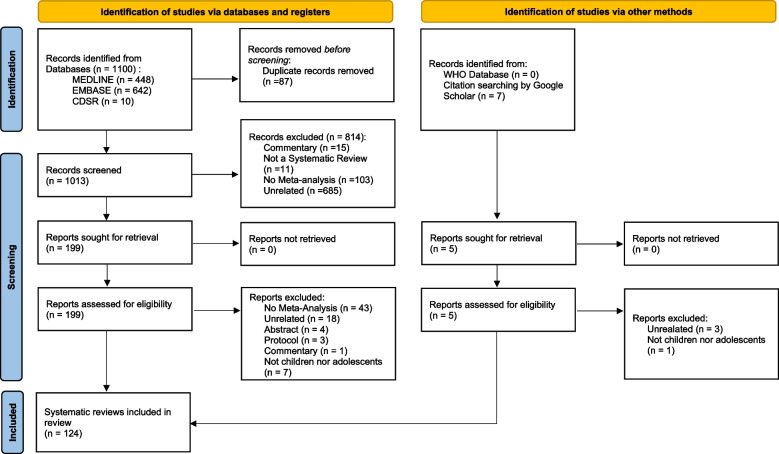
The PRISMA flowchart. This flow diagram provides a visual summary of the screening and selection processes, illustrating the number of articles recorded at each different stage

We retrieved a total of 1100 records from databases and registers (Fig. 1). After removing duplicates (*n* = 87), the title and abstract of 1013 records were screened against including criteria, and 814 records were excluded. Cohen’s kappa coefficient for title and abstract screening was 0.97 (95% CI 0.95–0.99). The full-text analysis was conducted on the remaining 199 records, 76 records were excluded, and 1 additional record was added through citation searching of reference lists. The list of excluded studies with reasons for exclusion is detailed in Additional file 4. Cohen’s kappa coefficient for full-text screening was 0.93 (95% CI 0.86–0.94), confirming excellent inter-examiner reliability. Ultimately, 124 systematic reviews with meta-analyses were included for data extraction and further analysis (Additional file 5) [4, 6, 20, 37–157]. Cohen’s kappa coefficient for data extraction was 0.90 (95% CI 0.89–0.91).

**Fig. 2:**
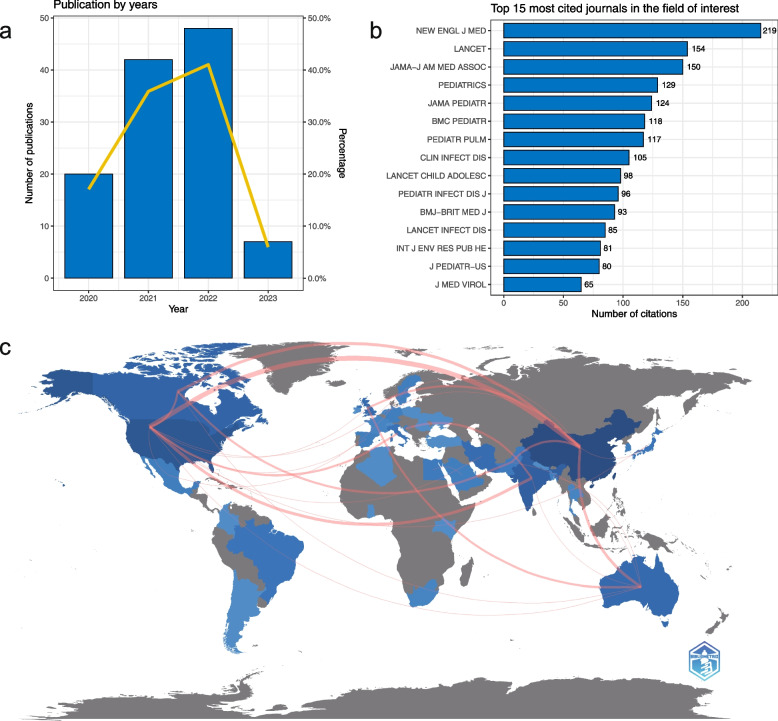
Bibliometrics for included systematic reviews. **a** Number of included publications by years. **b** Top 15 most cited journals in the field of interest. **c** The geographical collaborative distribution of included systematic reviews

All the included systematic reviews were published in 2020–2023. There was an increased trend of publishing relevant systematic reviews (Fig. 2a). The top 15 most cited journals in the field of interest are shown in Fig. 2b, in which 5 journals are multidisciplinary, 7 journals are focusing on pediatrics, and 3 journals are focusing on infectious disease and virology. Based on the number and relationship of publications in each country, a collaborative network was constructed and visualized (Fig. 2c). China, the USA, Australia, India, and the UK shared most collaborations.

Most included studies adhered to PRISMA guidelines (*n* = 99, 78.6%), followed by Meta-analyses of Observational Studies in Epidemiology (MOOSE) guidelines (*n* = 12, 9.5%) or a combination of multiple reporting guidelines (*n* = 9, 7.1%). However, a small portion of studies (*n* = 7, 7.9%) did not report following any systematic review reporting guidelines. To assess methodological quality, the Newcastle–Ottawa Scale (NOS) was used in most studies (*n* = 36, 28.6%), followed by the National Institutes of Health (NIH) Quality Assessment Tool (*n* = 14, 11.1%) and Joanna Briggs Institute (JBI) tools (*n* = 13, 10.3%).

In addition to encompassing the field of neonates for COVID19-diagnosed mothers, other domains include children and adolescents aged 0–19 years. We also explicitly indicate the age group sources of the relevant evidence for each subset within these domains. The descriptive characteristics of included meta-analyses under five major outcome domains (physical, psychological/cognitive, quality of life, social, and health system) are summarized in Tables 1 and 2. For pre-defined COVID-19 condition domains, the six domains were not evenly distributed: most topics focused on laboratory-confirmed infections (*n* = 60; 48.4%), followed by health impacts during the pandemic (*n* = 21; 16.9%), adverse events (AEs) caused by the COVID-19 vaccine (*n* = 20; 16.1%), COVID-19 associated multisystem inflammatory syndrome (MIS-C) (*n* = 15; 12.1%), newborns from diagnosed mothers (*n* = 6; 4.8%), and long-COVID (*n* = 2; 1.6%).

**Table 1:** Descriptive statistics of the included single-arm meta-analytical evidence

	Physical	Psychological/cognitive	Quality of life	Health system	Social
Number of meta-analyses	1497	27	10	11	6
Number of studies, median (min–max)	13 (1–1403)	13 (2–129)	11 (2–63)	3 (3–18)	6.5 (6.5–8)
Number of participants, median (min–max)	385 (1–11,595,017)	21,330 (176–1,241,604)	29,017 (150–29,017)	7046 (278–963,860)	73,431 (172–473,870)
*I*^2^ (*N*)					
*I*^2^ > 50%	681	17	1	2	2
*I*^2^ ≤ 25%	209	0	0	0	0

**Table 2:** Descriptive statistics of the included pairwise meta-analytical evidence

	Physical	Psychological/cognitive	Quality of life	Health system	Social
Number of meta-analyses	255	16	5	15	10
Number of studies, median (min–max)	6 (1–89)	3 (2–21)	3 (2–63)	4 (2–55)	14.5 (5–30)
Number of participants, median (min–max)	1674 (29–6,236,763)	10,737.5 (790–1,494,081)	23,033 (19,362–28,766)	7700 (43–3,231,572)	79,471 (172–310,585)
*P*-value (*N*)					
*P*-value < 10^−6^	14	7	0	1	0
*P*-value < 10^−3^	22	3	1	0	1
*P*-value < 0.05	109	1	0	5	5
*I*^2^ (*N*)					
*I*^2^ > 50%	112	14	4	9	9
*I*^2^ ≤ 25%	82	1	1	4	1
Overall grading (*N*)					
Not significant	111	5	4	9	4
Weak	116	1	0	5	5
Suggestive	16	6	1	0	1
Highly suggestive	8	3	0	1	0
Strong	4	1	0	0	0

### Methodological quality assessment

The Cohen kappa score for the AMSTAR-2 assessments was 0.89 (95% CI 0.83–0.91). No meta-analysis gained high overall confidence for methodological quality, 1 with moderate (0.8%), 18 studies were scored as low quality (14.5%), and 105 studies presented as critically low quality (84.7%) (Additional file 6). For critical flaws, most studies did not report a list of excluded studies and justify the exclusions (*n* = 116, 93.5%). In addition, inadequate accounting for the risk of bias (RoB) in primary studies when interpreting/discussing the results (*n* = 92, 74.2%), lacking report review methods as a priori (*n* = 60, 48.4%), and neglecting publication bias analysis during conducting relevant meta-analyses (*n* = 22, 17.7%) were also evident critical flaws. Most included studies have non-critical flaws such as lacking reports on the sources of funding (*n* = 115, 92.7%), inadequate assessment of the impact of individual RoB when performing evidence synthesis (*n* = 105, 84.7%), inadequate discussion of heterogeneity (*n* = 80), and lacking statements of the study designs for inclusion (*n* = 50, 40.3%).

### COVID-19-related evidence from single-arm meta-analyses

**Fig. 3:**
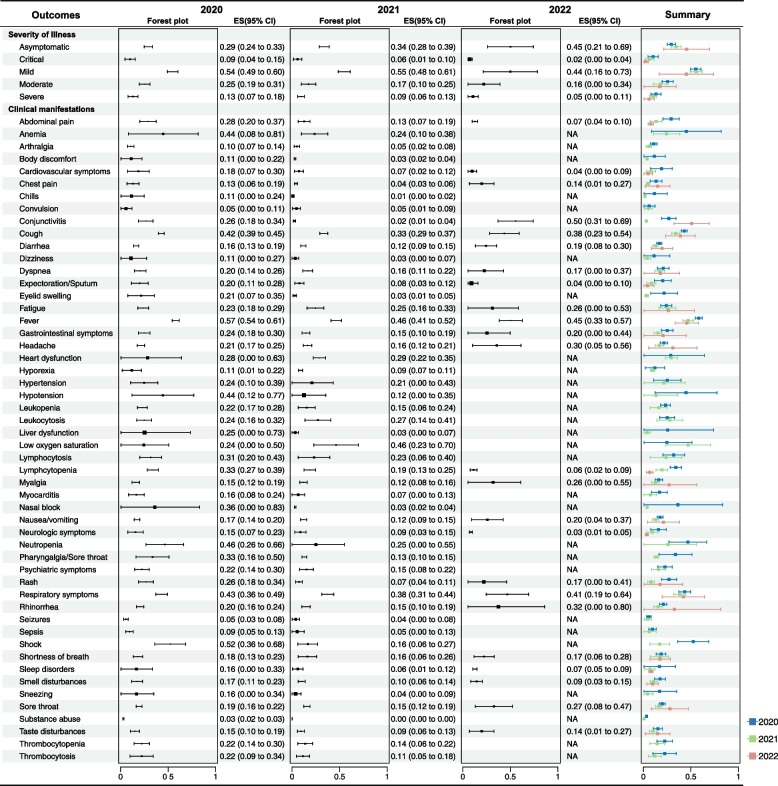
Forest plot for disease severity, and clinical manifestation associated with single-arm meta-analytical evidence of laboratory-confirmed COVID-19. Data are presented as effect size (ES) with 95% confidence intervals (CI). NA not available

**Fig. 4:**
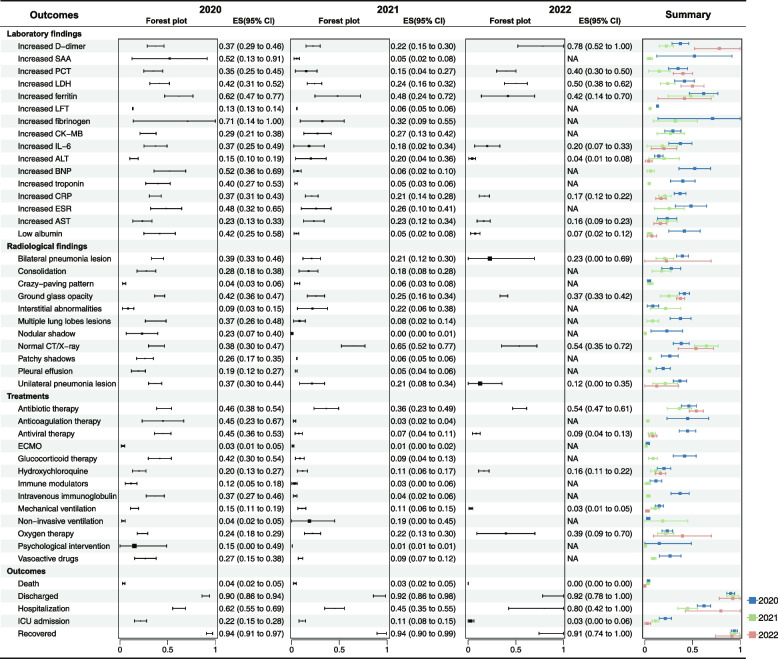
Forest plot for laboratory findings, radiological findings, treatment, and outcomes associated with single-arm meta-analytical evidence of laboratory-confirmed COVID-19. Data are presented as effect size (ES) with 95% confidence intervals (CI). SAA serum amyloid A, PCT procalcitonin, LDH lactate dehydrogenase, LFTs liver function tests, CK-MB creatine kinase-MB, IL-6 interleukin-6, ALT alanine transaminase, BNP brain natriuretic peptide, CRP C-reactive protein, AST aspartate aminotransferase, CT computed tomography, ECMO extracorporeal membrane oxygenation, ICU intensive care unit, NA not available

In total, 1551 meta-analytical comparisons were included in this umbrella review. These single-armed meta-analyses commonly utilized prevalence as effect size (*n* = 1464; 94.4%). Figures 3 and 4 present a comprehensive overview based on the reanalysis of primary data (Additional file 7) on the disease severity, clinical manifestations, laboratory and radiological findings, treatments, and outcomes related to laboratory-confirmed COVID-19.

#### Disease severity

In the past 3 years, mild symptoms consistently remained the primary manifestation of the disease, with a prevalence of approximately 50%. In contrast, the prevalence of severe and critical symptoms consistently stayed below 15% over the same period, showing a declining trend year by year. Notably, there was an upward trend in the prevalence of asymptomatic cases. In 2020, the percentage was 29.0% (95% CI 24.0–33.0%), which increased to 34.0% (95% CI 28.0–39.0%) in 2021, and further increased to 45.0% (95% CI 21.0–69.0%) in 2022.

#### Clinical manifestations

Fever consistently emerged as the most frequently reported symptom over the past 3 years, with a prevalence close to 50%. Concurrently, respiratory symptoms, primarily cough, also sustained a prevalence exceeding 30% throughout this period. Noteworthy patterns emerged in 2020, indicating a higher incidence of hematologic symptoms such as anemia, lymphocytosis, lymphocytopenia, neutropenia, thrombocytopenia, and thrombocytosis, with prevalence rates ranging from 22 to 46%. Subsequent observations in 2021 revealed a more frequent occurrence of low oxygen saturation (46%, 95% CI 23.0–70.0%) compared to preceding years. However, conjunctivitis (50.0%, 95% CI 31.0–69.0%) and rhinorrhea (32.0%, 95% CI 0–80.0%) appeared to be more prevalent in 2022. Conversely, cardiovascular and neurologic symptoms exhibited considerably lower combined prevalence rates of 4.0% (95% CI 0–9.0%) and 3.0% (95% CI 1.0–5.0%), respectively.

#### Laboratory findings

Most laboratory results were reported in studies conducted between 2020 and 2021. In 2020, there seemed to be a more pronounced prevalence of abnormal laboratory markers, with abnormalities observed in fibrinogen, troponin, ferritin, SAA, BNP, ESR, and albumin, each surpassing 40%. Contrastingly, by 2021, the prevalence of albumin, troponin, BNP, and SAA abnormalities did not exceed 6%. In 2022, abnormalities in D-dimer, PCT, and LDH became more prevalent, surpassing the threshold of 40%.

#### Radiological findings

In 2021 and 2022, normal CT scans and lung chest radiographs exhibited a high prevalence of 65.0% (CI 52.0–77.0%) and 54.0% (CI 35.0–72.0%), respectively, surpassing the prevalence of 38.0% (CI 30.0–47.0%) observed in 2020. Abnormal imaging findings such as bilateral pneumonia lesions, unilateral pneumonia lesions, and multiple lung lobe lesions were notably prevalent in 2020, exceeding 30% prevalence, which markedly decreased in the subsequent 2 years.

#### COVID-19-related treatments

During the 3-year duration, antibiotics emerged as a common treatment for COVID-19 in youths. However, therapies such as anticoagulation, antiviral medications, glucocorticoids, and intravenous immunoglobulin were sparingly utilized, each with a prevalence not exceeding 37.0%. The use of oxygen therapy exhibited an increasing trend, reaching 39.0% (CI 9.0–70.0%) in 2022. Conversely, mechanical ventilation was predominantly employed in 2020, with a prevalence of 15.0% (95% CI 11.0–19.0%), which notably decreased to 3.0% (95% CI 1.0–5.0%) by 2022.

#### COVID-19-related outcomes

Reported outcomes and prognoses for COVID-19 patients among youths consistently show relatively high rates of recovery and discharge. Additionally, a declining trend in the prevalence of ICU admissions and mortality has been observed year by year. Merely 3% (CI 0–6%) of cases necessitated ICU admission, and there were no reported mortalities in 2022.

Additional File 8 provides further details on COVID-19-related evidence obtained from single-arm meta-analyses. Additional file 9 [40, 69, 75, 97, 108, 109, 117, 122, 123, 132, 137] summarizes single-arm evidence about COVID-19-associated MIS-C. Additional file 10 [62, 156, 157] summarizes single-arm evidence about newborns from COVID-19-diagnosed mothers. Additional file 11 [47, 87] summarizes single-arm evidence about long-COVID. Additional file 12 [54, 67, 77, 139, 153] summarizes single-arm evidence about events caused by the COVID-19 vaccine. Additional file 13 [49, 50, 58, 82, 99, 113, 142] summarizes single-arm evidence about health impacts during the pandemic.

### COVID-19-related evidence from pairwise meta-analyses

Three hundred one meta-analytic comparisons from 47 pairwise systematic reviews were analyzed. Out of these, only 1.7% (*n* = 5) were considered to have strong meta-analytical evidence, while 4.0% (*n* = 12) and 8.0% (*n* = 24) were categorized as highly suggestive and suggestive evidence respectively (Table 2). A stricter *P*-value threshold revealed that 8.9% (*n* = 27) and 7.3% (*n* = 22) of the meta-analyses had significance at 10^−3^ and 10^−6^. The remaining 39.9% (*n* = 120) were statistically significant (*p* < 0.05). In terms of heterogeneity, approximately 49.2% (*n* = 148) of the included meta-analyses had high heterogeneity (*I*^2^ > 50%), while 29.6% (*n* = 89) presented low heterogeneity (*I*^2^ ≤ 25%).

**Fig. 5:**
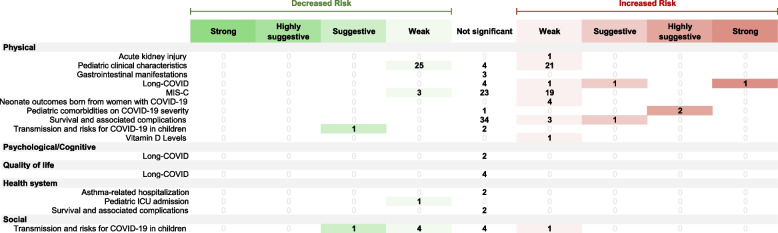
Evidence grading on direct effects of COVID-19 on physical, psychological/cognitive, quality of life, social, and health system domains. The right side illustrates associations that elevate the risk for the respective health condition (in red), while the left side demonstrates associations that lower the risk (in green). COVID coronavirus disease, MIS-C multisystem inflammatory syndrome in children, ICU intensive care unit

A total of 176 meta-analyses (58.4%) explored the direct impact of COVID-19 on children and adolescents. The existing evidence base is largely skewed in favor of a biomedical evaluation of health outcomes in COVID-19-infected individuals, focusing primarily on physical outcomes and suggesting an increased risk of impaired health (Fig. 5). Only one had strong meta-analytical evidence: long COVID-19 impact on physical outcomes (*n* = 1), while pediatric comorbidities presented highly suggestive evidence of impacting COVID-19 severity (*n* = 2). In addition, suggestive evidence was found on the effect of long-COVID (*n* = 1) as well as survival and associated complications (*n* = 1) on physical outcomes. Furthermore, transmission and risks for COVID-19 in children present suggestive evidence on both physical (*n* = 1) and social (*n* = 1) outcomes. A total of 84 meta-analyses indicated weak evidence, leaving 85 meta-analyses with no statistically significant results.

**Fig. 6:**
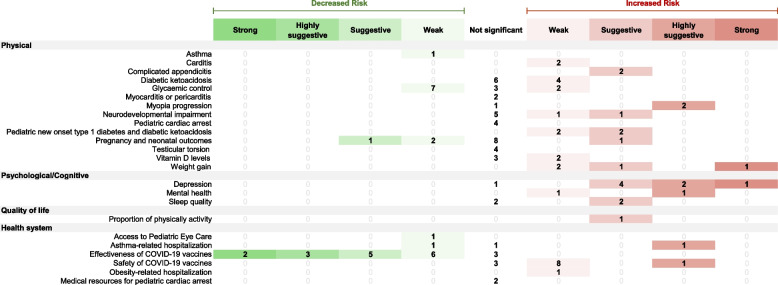
Evidence grading on indirect effects of COVID-19 on physical, psychological/cognitive, quality of life, social, and health system domains. The right side illustrates associations that elevate the risk for the respective health condition (in red), while the left side demonstrates associations that lower the risk (in green). COVID coronavirus disease

The COVID-19 pandemic’s indirect impacts on children and adolescents were reported in 125 meta-analyses (41.6%). Existing evidence tends to show an increased health risk for children and adolescents, particularly in physical, psychological, and quality of life outcomes. Specifically, among these meta-analyses, two were categorized as having strong evidence (Fig. 6), indicating an elevated risk of depression (*n* = 1) and weight gain (*n* = 1). Five meta-analyses presented highly suggestive evidence, associating the increased risk with myopia progression (*n* = 2), depression (*n* = 2), and mental health issues (*n* = 1). The population examined in the meta-analysis, which yielded highly suggestive evidence regarding mental health issues, consisted of children aged 5 to 13 years. In terms of health system outcomes, an additional meta-analysis offered highly suggestive evidence, highlighting an increased risk of asthma-related hospitalization during the COVID-19 pandemic. A further twenty meta-analyses had suggestive evidence, ten of which pertained to associations that already received strong or highly suggestive evidence. The remaining ten meta-analyses showed an increased risk in outcomes, including complicated appendicitis (*n* = 2), neurodevelopmental impairment (*n* = 1), pediatric new-onset type 1 diabetes and diabetic ketoacidosis (*n* = 2), pregnancy and neonatal outcomes (*n* = 1), sleep quality (*n* = 2), and physical activity decline (*n* = 1). A total of 53 meta-analyses were supported by weak evidence, while the remaining 48 meta-analyses did not have nominally statistically significant findings. Additionally, the health system outcomes section notably emphasized evidence concerning the effectiveness and safety of COVID-19 vaccines. Two more meta-analyses focusing on the effectiveness of COVID-19 vaccines (*n* = 2) were categorized as having strong evidence (Fig. 6). Four other meta-analyses presented highly suggestive evidence, reporting the effectiveness (*n* = 3) and safety (*n* = 1) of COVID-19 vaccines. The meta-analysis that provided highly suggestive evidence regarding the effectiveness of COVID-19 vaccines focused on a study population comprising children aged 5 to 11 years.

### Number of additional studies needed to change current pairwise meta-analytic evidence

For strong evidence, the median fail-safe number (FSN) was 8 (range 4–25). For highly suggestive and suggestive evidence the median FSN were 13 (range 4–158) and 11 (range 1–163), respectively. The FSN in 73.2% of these studies (*n* = 30) were higher than the number of studies included in the meta-analyses, meaning that adding studies in the future is unlikely to change the robustness of the statistical significance for these metanalytic evidence. For weak evidence, the median FSN was 11 (range 1–2569), and only 37.8% of studies (*n* = 48) had FSN higher than the number of studies included in the meta-analyses.

## Discussion

### Main findings from the single-arm meta-analytical evidence

Single-arm meta-analyses have provided extensive evidence on the prevalence and estimations across six domains associated with COVID-19 condition. These domains include laboratory-confirmed COVID-19, COVID-19-associated MIS-C, newborns from COVID-19-diagnosed mothers, long-COVID, events caused by the COVID-19 vaccine, and health impacts during the pandemic.

In this umbrella review, we specifically focus on laboratory-confirmed COVID-19 infections. Through reanalyzing the primary studies from these meta-analyses by removing overlapping data and remapping the actual data collection year, we investigated the distinct clinical characteristics, management, and outcomes of children and adolescents with COVID-19 infections from 2020 to 2022. Clinical manifestations among children exhibited variability over the years, illustrating a diverse range of features. More than half of these manifestations demonstrated a downward trend over time. Our analysis illustrates prevailing patterns in prevalence, indicating an increase in asymptomatic cases and a decrease in other severity levels of cases. In terms of COVID-19-related outcomes, there was a decrease in both admissions to the intensive care unit (ICU) and mortality rates over the years, while the number of discharged and recovered cases remained relatively stable. Interestingly, hospitalization rates rebounded in 2022, potentially attributed to the emergence and spread of novel COVID-19 strains with immune escape mechanisms [158]. COVID-19-related MIS-C is characterized by recurring high fever, damage to multiple organs, heightened inflammatory indicators, and frequent severe outcomes. Newborns from mothers diagnosed with COVID-19 generally experienced mild symptoms and had a low risk of vertical transmission, although adverse health outcomes were still possible. Furthermore, our findings suggest that children and adolescents affected by long-COVID commonly report symptoms such as fatigue, dyspnea, sore throat, mood changes, and sleep disorders. For events caused by COVID-19 vaccines, we observed that AEs were more frequently reported following booster doses compared to earlier doses. Solicited local and systemic AEs were also found to be common across all doses. Lastly, regarding the domain of pandemic lockdown, our findings reveal a significant correlation between social isolation and adverse effects on the mental health, sleep habits, and physical activity of children and adolescents.

### Main findings from the pairwise meta-analytical evidence

Among the pairwise meta-analyses, we observed strong evidence for five effects and highly suggestive evidence for 12 effects. These results were supported by highly significant findings. Based on the available evidence, we have classified the strong and highly suggestive evidence into three primary categories: (i) the direct effects of COVID-19 infection on children’s health, (ii) the indirect impacts of the COVID-19 pandemic on children’s well-being, and (iii) the efficacy and safety of COVID-19 vaccines. The direct effect is demonstrated by a higher risk of severe COVID-19 in children with comorbidities and persistent negative health challenges resulting from long-COVID. Several factors can explain the manifestations linked with long-COVID, including persistent acute organ damage, the presence of the virus in the body, and the activation of autoimmune mechanisms that target both the COVID-19 virus and host tissues [159]. Moreover, the indirect effects of COVID-19 had strong correlations with significant economic disruptions, increased social isolation, mental health challenges, and a shift towards remote work and online activities for children and adolescents [160]. Our study revealed that these indirect impacts are manifested in increased anxiety and depression levels, accelerated myopia progression, as well as significant increases in body weight and BMI during the COVID-19 pandemic home quarantine. The effectiveness and safety of COVID-19 vaccines have been confirmed to a certain extent, although several potential AEs have been reported.

### Similarities and disparities of the previous and current studies

In our study, we investigated and presented evidence of the negative physical correlations observed in individuals with long-COVID. Previous umbrella reviews, which primarily focused on adults, have examined the long-term consequences experienced by COVID-19 survivors beyond the acute phase. However, these evaluations were limited by the absence of graded evidence [161, 162]. In contrast, our study pooled single-arm meta-analyses specifically on long-COVID in children and adolescents, demonstrating similar outcomes. Nonetheless, it is important to note that these findings did not achieve high-level evidence ranking like those obtained through pairwise meta-analysis. This limitation can be attributed to the constraints imposed by the study design and sample size, which were influenced by limited time, resources, and evolving understanding of long-term consequences associated with acute COVID-19 [17]. The management of individuals with post-COVID conditions presents significant challenges due to the diverse range of symptoms, unpredictable duration, and absence of definitive risk factors. Furthermore, the symptoms of long-COVID can manifest in various combinations from patient to patient, with fluctuations in both frequency and severity. This dynamic nature adds an extra layer of complexity to the issue of long-COVID.

This umbrella review offered a comprehensive analysis of the correlation between the COVID-19 pandemic and the mental health of children and adolescents. Similar to previous umbrella reviews, we observed differing impacts regarding various mental health issues among this demographic. However, the methodological approaches of these umbrella reviews varied considerably. Due to significant heterogeneity in the methods and outcomes of the reviews included, some lead to a narrative synthesis presentation and an omission in evidence grading [21, 163]. One umbrella review [164] conducted a reanalysis of systematic reviews to re-examine data from preliminary studies captured within each systematic review, aiming to reduce potential biases that could have previously impacted the assessment of mental health during the pandemic. Despite the absence of evidence grading, the insights provided by this evidence remain significant. It is also important to acknowledge that disentangling the direct impact of the pandemic on the mental health of children and adolescents remains challenging due to the complexity of mental health disorders. Additionally, the implementation of bundled mitigation strategies at national or subnational levels complicates the identification of individual strategies that may have contributed to exacerbated mental health effects. To sum up, the integration of our current findings with previous studies has the potential to substantially enhance policymaking and practice in the field of mental and child health, thereby guiding future research endeavors to strengthen the global knowledge base.

### Mechanisms and implications from current evidence

#### The gradual reduction of health risk of COVID-19 in children and adolescents

The health risk of COVID-19 in children and adolescents appears to decrease gradually over time. Our research also indicates a rise in the number of asymptomatic cases among children over the years, alongside a decline in ICU admissions and mortality rates. These trends may be attributed to a globally prevalent variant strain during the period when the data included in the study was collected. With the ongoing pandemic, the Omicron variant (B.1.1.529) emerged as the dominant strain during that time, surpassing Delta in its transmission rate [165]. Additionally, the Omicron variant has demonstrated a lower rate of hospitalization, ICU admission, invasive mechanical ventilation (IMV), and in-hospital deaths, along with a higher prevalence of asymptomatic cases compared to the Delta variant [166, 167]. These observations suggest that the Omicron variant may have reduced pathogenicity and milder symptoms than previous variants. Our findings indicate that the most frequently reported symptoms are fever and cough, which aligns with the previous umbrella reviews not limited by age [20]. However, our study discovered a higher incidence of conjunctivitis in 2022, with a rate of 48.4%. This symptom is considered rare, as its prevalence among positive cases typically ranges from 0.8 to 31.6% [168]. Our findings indicate that the most frequently reported symptoms are fever and cough, which aligns with the previous umbrella reviews not limited by age [20]. However, our study discovered a higher incidence of conjunctivitis in 2022, with a rate of 48.4%. This symptom is considered rare, as its prevalence among positive cases typically ranges from 0.8 to 31.6% [168]. It has been established that frequency of hand-eye contact presents as an independent risk factor for COVID-19-related conjunctivitis [169].

Another key finding in our study is the lower health risk of COVID-19 for children and adolescents when compared to adults. The current umbrella review provides ample evidence to support the notion that most children and adolescents infected with COVID-19 exhibit mild or even asymptomatic symptoms. During the Omicron epidemic, the proportion of asymptomatic cases across all age groups was 33.72% in 2022 and 23.57% in 2021, both of which indicate a lower proportion of asymptomatic cases among adults compared to our reported findings [170]. Umbrella reviews without age limitations often overlook the critical role of age in influencing both COVID-19 susceptibility and disease severity [171]. The relative susceptibility among children and adolescents aged 0–19 years was also notably lower, ranging from 6 to 16% compared to adult groups, with the rate of critical illness in adults being 4.95 times higher than that in children [172]. However, the reasons behind this phenomenon remain incompletely understood, despite available data suggesting similar viral loads in both children and adults at the time of presentation [173]. Several hypotheses have been proposed to explain the disparity in COVID-19 severity between younger and older individuals, including more efficient local tissue responses [174], better thymic function [175], and cross-reactive immunity [176]. Currently, the prevailing viewpoint suggests that the lower incidence and severity of COVID-19 disease in infants can be explained by maternal immune transfer [177], an immature immune system [178], and reduced expression of COVID-19 attachment receptors such as ACE-2 [179]. However, there is also evidence suggesting that maternal COVID-19 may impact the neonatal immune system, potentially leading to an exacerbated inflammatory response and immune activation [180]. Consequently, the programming of the neonatal immune system by the maternal inflammatory milieu induced by COVID-19 remains uncertain.

From the perspective of childhood growth evolution, recent evidence suggests that the body’s energy allocation often avoids costly systemic inflammatory responses [181]. By prioritizing disease tolerance rather than maximal resistance, children are more likely to experience mild or even asymptomatic illness. However, this approach may result in a lower efficiency in clearing viruses, which could lead to some degree of viral persistence and the subsequent manifestation of other diseases associated with such persistence. Therefore, personalized treatments tailored to the severity of the disease should be implemented for pediatric patients, along with thorough long-term monitoring and follow-up care. Furthermore, our findings indicate that dysautonomia often presents symptoms commonly associated with long-COVID. However, whether dysautonomia is a direct consequence of COVID-19 infection or an immune-mediated process remains unclear [182]. Although current evidence suggests that long-COVID has a relatively milder health impact on pediatric patients compared to adults, the life course implications, including psychological, social, and economic impacts, are not fully revealed by current evidence and require more extensive long-term follow-up studies [183]. Insufficient lifecycle observations limit the evidence of the impact of long-COVID on pediatric patients, requiring caution when interpreting the relatively lower impact on this group. Nevertheless, school closure and social distancing measures may further compromise the social well-being of children with long-COVID symptoms [184]. It is crucial to implement social policies that promote the return to school and participation in extracurricular activities among young individuals to address specific risk factors like obesity associated with long-COVID. Additionally, this can help mitigate the negative cultural impact and psychological consequences caused by remote learning [184, 185].

The immune microenvironment, physiological and psychological factors, and social activities contribute to disparities in disease susceptibility among children and adolescents at different developmental stages [186, 187]. Previous literature has reported a higher susceptibility to severe COVID-19 and increased hospital admissions among pediatric patients aged ≤ 4 years or 12–17 years, indicating a bimodal age distribution [188]. Our research shows that serious consequences such as dyspnea, ICU admission, and death have also been observed and cannot be ignored, although a lower health risk of COVID-19 for children and adolescents due to uncommon vertical transmission during neonatal admission from infected mothers was observed.

However, at the umbrella review level, the current available primary and meta-analytical evidence has limited the evaluation process of COVID-19’s impact on age-specific subgroups of children and adolescents. Most of the included evidence has focused on the 0–19 age group as a whole [4, 6, 20, 37–151], with only a few studies examining specific age ranges. For example, Yasuhara J’s study included children and adolescents aged 12–19 years [152], while Watanabe A’s study included children aged 5–11 years [153], without direct comparison to other age-specific subgroups. The lack of age-specific research and comparisons poses challenges for subgroup analyses across various age groups, excluding neonatal groups that are typically distinguished in the literature and technically feasible to evaluate [154–157]. Therefore, our main analysis includes the 0–19 age group, with a separate domain for newborns from COVID-19-diagnosed mothers, to demonstrate the impact of COVID-19 on children and adolescents at different developmental stages.

#### The mental health risks of COVID-19-related social isolation on children and adolescents

Over the past 3 years, significant efforts have been made to control the spread of COVID-19 through various interventions, such as social distancing, mobility restrictions, and school closures. Nevertheless, these measures may have unintended and detrimental effects on the mental health of children and adolescents [189, 190]. The present umbrella review provides evidence supporting the idea that the pandemic has resulted in an increased burden of mental health concerns among this population, including conditions like depression and sleep disorders. These findings are consistent with previous research studies [21, 22]. However, their report indicated a pooled prevalence rate of 32% for depression (95% CI 27–38%) and 32% for anxiety (95% CI 27–37%) among children and adolescents worldwide following COVID-19 mitigation measures, which was lower than our findings. This suggests that the deterioration of mental health in the younger population during the pandemic may not solely be attributed to indirect impacts during the gradual relaxation of mandatory control measures in many countries. The underlying causes of this phenomenon may vary and encompass financial stressors [191], social isolation [192], physical health concerns [193], and heightened anxiety and fear stemming from the uncertainties of COVID-19 [194]. Therefore, it is crucial to monitor the negative impact on the mental health of children and adolescents in the future, with dedicated efforts aimed at enhancing their well-being. Policymakers and healthcare professionals should adopt a holistic approach that addresses these multifaceted issues to effectively mitigate the detrimental effects on mental health.

#### The safety and efficacy of vaccines for children

The present study indicates that COVID-19 vaccines effectively prevent severe illness and reduce transmission. Evidence from pooled studies in healthy children and adolescents suggests that the occurrence of AEs, including local and systemic AEs, is similar between the vaccine and placebo groups. Furthermore, serious AEs are mostly unrelated to vaccination [195]. Recent studies have also confirmed the favorable and safe response to COVID-19 vaccination among pediatric patients with inflammatory rheumatic diseases [196], endocrinological disorders [197], or inflammatory bowel disease [198], addressing concerns about potential AEs in vulnerable populations with inadequate or overactive immune responses. However, it is important to note that the evidence supporting these findings is currently limited to cross-sectional studies. Additionally, both local and systemic AEs were reported to occur slightly more frequently than in the adult population. Nonetheless, this does not suggest evidence against the vaccine’s safety, as reactogenicity is more common in young individuals than in adults [199]. Although side effects may vary depending on the person, they typically tend to be mild and temporary, similar to common childhood vaccines [200]. Based on emerging safety and efficacy data, along with increased vaccine availability, widespread vaccination is recommended, particularly for high-risk children.

#### COVID-19-related global policies related to children and adolescents

The impact of COVID-19-related global policies is a matter of great concern for the health and well-being of children and adolescents. The effects of policies and regulations implemented to control the COVID-19 outbreak are intricate and multifaceted. Policies that specifically target children and young people, such as school closures and healthcare restrictions, have direct consequences on their social interactions [201], education [202], and access to healthcare resources [203]. Moreover, policy decisions and measures have resulted in numerous social conflicts, further impacting the daily lives of children and adolescents. For example, children often find themselves caught in the midst of disputes between parents, friends, schoolmates, teachers, and activity leaders regarding COVID-19 measures [204]. Additionally, the social and economic repercussions of policy measures become apparent in later stages of the pandemic, underscoring the direct impact these challenges have on the everyday lives of children. As an illustration, school children from underprivileged families face multiple hurdles due to the pandemic, including financial insecurity and disparities in remote learning caused by a lack of digital devices [205]. These complex impacts make it difficult to establish a clear link between policies and child health, and limited research has focused on the long-term effects of policies on affected children. Furthermore, as COVID-19 continues to significantly impact the mental health of the general population, countries have developed and revised policies, guidelines, and new initiatives to address the psychological well-being of their citizens. These supportive policies further complicate the discussion surrounding policy impacts.

Evidence of the impact of COVID-19-related policies on children and adolescents is limited. As stated above, current meta-analytical evidence suggests that children may be less affected by certain social settings as a result of policy development, such as the reopening of schools and workplaces. However, the physical and psychological/cognitive effects of the virus may hinder a child’s ability to return to school for several weeks or months. Research on health system utilization in this area primarily focuses on medical resources, such as emergency services and ICU admissions. However, conflicting evidence exists, with only a limited number of studies available. Based on current meta-analytical data, it appears that health system utilization for life-threatening diseases and situations in younger patients is not significantly different from or may even be lower than the pre-pandemic era, except in cases involving younger patients with asthma and obesity. It is important to interpret these results cautiously since medical crowding and inadequate resources may overshadow the utilization of the health system by pediatric COVID-19 patients [206].

### Strengths and weaknesses

The strengths and weaknesses of this umbrella review are quite straightforward. First, one of the strengths is that we provided a comprehensive summary of the current available evidence by reviewing previous meta-analyses on the association between COVID-19 and various health outcomes in children and adolescents. Second, our study protocol was registered in PROSPERO, ensuring transparency and robustness in the planned analysis and results. Considering the worldwide prolonged transmission and evolving nature of COVID-19, this study holds clinical and social importance in shaping preventive strategies for children and adolescents in the post-pandemic era. Next, this study utilized a systematic approach, involving thorough searching, selection, and data extraction conducted by two independent authors with excellent inter-examiner reliability. Methodological quality and evidence classification were assessed using two established criteria, AMSTAR-2 [27] and evidence grading criteria [28, 29] to assess the methodological quality and evidence classification. Without limiting study designs and statistical significance, the current umbrella review filled the knowledge gaps and captured temporal changes in key aspects of COVID-19 in children and adolescents. However, some limitations still exist in this review. Firstly, most of the meta-analyses included in this review are based on observational studies, and the prospective or randomized study designs were not prominently featured. This observational nature of the studies introduces the potential for selection bias, as differences in baseline characteristics and confounding factors can exist among the study populations [207]. Consequently, these studies only provide associations between variables and cannot account for all possible confounders or factors that may influence the results. Secondly, many studies within this topic rely on self-reported data, which can be subjective and susceptible to recall bias [208]. The use of self-reporting may lead to inconsistencies or inaccuracies in the data collected. Moreover, the absence of a comparison group in single-arm studies makes it challenging to accurately establish the effects of COVID-19 on children and adolescents. The evaluation of the impact of COVID-19 on age-specific subgroups of children and adolescents is incomplete due to the limited availability of primary and meta-analytical evidence. The lack of age-specific research and comparisons poses challenges for subgroup analyses across various developmental stages, potentially resulting in an incomplete depiction of the differential impacts of COVID-19 across different developmental stages of children and adolescents. In this current study, we chose to present all available meta-analytical data from single-arm studies and categorized them into six predefined COVID-19 condition domains [25]. This approach allows us to illustrate the main findings of COVID-19 on children and adolescents in a time-dependent manner, considering the dynamic changes in disease severity, clinical manifestations, laboratory and radiological findings, treatment, and outcomes. Although this approach has its limitations, it provides valuable insights that can inform public health measures and interventions targeted at this population. It also emphasizes the need for further controlled studies tailored to address the specific impacts of COVID-19 on children and adolescents.

### Implications for practice and research

For practice, the effect estimates combined with different topic domains categorized from primary studies can help practitioners identify high-risk younger patients for COVID-19-related direct and indirect health outcomes. However, when it comes to children and adolescents with rare disease conditions, identifying high-risk groups remains a challenge. These individuals are often underrepresented in primary studies, likely due to decreased medical care utilization during the pandemic. This oversight may result in the neglect of rare disease conditions in both practice and policy decisions. Based on the complete picture of available evidence and the COVID-19 and health domains linking mechanisms, policymakers could better prioritize the prevention and intervention methods for children and adolescents affected by COVID-19 (in)directly. Connecting these individuals with appropriate support services is another key question in COVID-19 management. The effects and evidence summarized in this umbrella review suggests that such services could include medical/surgical management of physical illness and comorbidities, psychotherapy, physio and occupational therapy, and nursing. Multidisciplinary collaboration units dedicated to younger COVID-19 patients or pandemic lockdowns can be invaluable in providing tailored prevention and intervention strategies [209, 210]. Shifting the focus of COVID-19 global health emergency management to long-term management, alongside other infectious diseases, can accelerate the implementation of surveillance systems for COVID-19-related health consequences and rehabilitation programs for affected younger patients.

For research, the effect estimates in this review are heavily focused on the physical outcomes, investigating the relationship between COVID-19 and disease severity, clinical manifestations, laboratory/radiological findings, treatment, and outcomes in younger patients. Gaps still exist between available evidence in non-physical outcomes and current clinical practice. Furthermore, some meta-analytical results did not reveal significant associations with many COVID-19-related health conditions, such as the direct effects of long-COVID on the psychological/cognitive well-being and quality of life of younger patients in pairwise studies, which lead to the overall evidence consistency due to poor meta-analytic or methodological reasons. Future studies can provide a more nuanced understanding of how COVID-19 affects children and adolescents at different developmental stages by expanding the scope of research to include a wider range of age groups. This will enable the analysis of the impacts of COVID-19 on various stages of childhood and adolescence, facilitating the development of tailored interventions and guidelines specifically designed for these populations. It is important to note that this does not imply the absence of robust associations, but rather the current body of evidence does not yet support such inferences [211]. Therefore, updating and transforming this review into a living review would be valuable in incorporating emerging evidence from future meta-analytical studies on the impacts of COVID-19 on children and adolescents.

## Conclusions

In summary, this work evaluated the meta-analytical evidence regarding the associations between the (in)direct COVID-19 effects and multiple health and well-being domains of children and adolescents. The findings of this study can serve as a comprehensive evidence map to inform, educate, and train various interested parties, including key stakeholders such as policymakers, patients, and practitioners. It is also important to acknowledge that the majority of the findings and recommendations presented in this study are derived from observational studies that have methodological limitations. Thus, it is crucial to exercise caution when interpreting the results and implementing the implications of this study. Additionally, future research should prioritize the execution of high-quality studies utilizing prospective, long-term, or randomized designs to more comprehensively understand the causal effects of COVID-19, both direct and indirect, on children and adolescents.

### Supplementary Information

**Additional file 1.** Differences between protocol and review.

**Additional file 2.** PRISMA 2020 checklist.

**Additional file 3.** Search strategy.

**Additional file 4.** List of excluded studies with justification for exclusion.

**Additional file 5.** Detailed information on the characteristics of the included systematic reviews.

**Additional file 6.** AMSTAR2 methodological quality assessments.

**Additional file 7.** Primary studies data extracted from single-armed meta-analyses of laboratory-confirmed COVID-19.

**Additional file 8.** Additional COVID-19-related evidence from single-armed meta-analyses.

**Additional file 9.** Effect sizes and forest plots on COVID-19-associated multisystem inflammatory syndrome.

**Additional file 10.** Effect sizes and forest plots on newborns from COVID-19-diagnosed mothers.

**Additional file 11.** Effect sizes and forest plots on long-COVID.

**Additional file 12.** Effect sizes and forest plots on events caused by the COVID-19 vaccine.

**Additional file 13.** Effect sizes and forest plots on health impacts during the pandemic.

## Data Availability

All data generated and analyzed that support findings in this study are supplemented in the Supplementary Information. The Rscript snippets for generating descriptive summaries and reanalyzing single-arm studies are available on GitHub using the following link: https://github.com/Piperacillin/Child_COVID19_Umbrellareview.

## Abbreviations

AEs: Adverse events
AMSTAR-2: A Measurement Tool to Assess Systematic Reviews 2
COVID-19: Coronavirus disease 2019
CRP: C-reactive protein
JBI: Joanna Briggs Institute
LDH: Serum lactate dehydrogenase
MIS-C: Multisystem inflammatory syndrome
MOOSE: Meta-analyses of Observational Studies in Epidemiology
NIH: National Institutes of Health
NOS: Newcastle-Ottawa Scale
PCT: Procalcitonin
PRISMA: Preferred Reporting Items for Systematic Reviews and Meta‐Analyses
RoB: Risk of bias
SARS-CoV-2: Severe acute respiratory syndrome coronavirus 2
WBC: White blood cell
WHO: World Health Organization
