# Supplementary material for: Evidence linking COVID-19 and the health/well-being of children and adolescents: an umbrella review
Source: BMC Med. 2024 Mar 13;22:116. doi: 10.1186/s12916-024-03334-x (PMC10938697; doi:10.1186/s12916-024-03334-x)
Supplement: Supplementary file 1 — Additional file 1. Differences between protocol and review. [file 12916_2024_3334_MOESM1_ESM.docx]

**Additional file 1: Differences between protocol and review**

To adhere to traditional title formatting and reading habits, we modified the title by relocating "an umbrella review" from the beginning to the end.

To reduce redundancy and streamline the description of the methodology, we consolidated the information from the protocol sections on "Types of study to be included," "Participants/population," "Intervention(s), exposure(s)," and "Context" into the inclusion criteria section of the manuscript.

To conduct citation searching of full texts, Google Scholar was employed as our primary supplementary resource.

The search records from the Cochrane Database Abstracts of Reviews of Effects (DARE) were not included due to the absence of any COVID-19-related records in this database.
