# Supplementary material for: Evidence linking COVID-19 and the health/well-being of children and adolescents: an umbrella review
Source: BMC Med. 2024 Mar 13;22:116. doi: 10.1186/s12916-024-03334-x (PMC10938697; doi:10.1186/s12916-024-03334-x)
Supplement: Supplementary file 3 — Additional file 3. Search strategy. [file 12916_2024_3334_MOESM3_ESM.docx]

**Additional file 3: Search strategy**

**MEDLINE via Ovid**

#1 Coronavirus/

#2 Coronavirus Infections/

#3 COVID‐19/

#4 SARS‐CoV‐2/

#5 COVID‐19.rs.

#6 severe acute respiratory syndrome coronavirus 2.os.

#7 (2019 nCoV or 2019nCoV or 2019‐novel CoV).tw,kf.

#8 (corona vir* or coronavir* or neocorona vir* or neocoronavir*).tw,kf.

#9 COVID.mp.

#10 COVID19.tw,kf.

#11 (nCov 2019 or nCov 19).tw,kf.

#12 ("SARS‐CoV‐2" or "SARS‐CoV2" or SARSCoV2 or "SARSCoV‐2").mp.

#13 ("SARS coronavirus 2" or "SARS‐like coronavirus" or "Severe Acute Respiratory Syndrome Coronavirus‐2").mp.

#14 or/1‐13

#15 exp Child/

#16 exp Adolescent/

#17 exp Infant/

#18 (child* or boy* or girl* or adolescen* or teen* or toddler* or preschooler* or pre‐schooler* or baby or babies or infant*).tw.

#19 or/15-18

#20 (“meta-analy*” OR “meta-regression” OR “meta-synthesis”). ti,ab,kw

#21 meta-analysis/

#22 20 or 21

#23 14 and 19 and 22

**Embase via Ovid**

#1 coronaviridae/

#2 exp coronavirinae/

#3 exp coronavirus infection/

#4 (2019 nCoV or 2019nCoV or 2019‐novel CoV).ti,ab,kw.

#5 (corona vir* or coronavir* or neocorona vir* or neocoronavir*).ti,ab,kw.

#6 COVID.af.

#7 COVID19.ti,ab,kw.

#8 (nCov 2019 or nCov 19).ti,ab,kw.

#9 ("SARS‐CoV‐2" or "SARS‐CoV2" or SARSCoV2 or "SARSCoV‐2").af.

#10 ("SARS coronavirus 2" or "SARS‐like coronavirus" or "Severe Acute Respiratory Syndrome Coronavirus‐2").af.

#11 or/1‐10

#12 exp Child/

#13 exp Adolescent/

#14 exp Infant/

#15 (child* or boy* or girl* or adolescen* or teen* or toddler* or preschooler* or pre‐schooler* or baby or babies or infant*).tw.

#16 or/12-15

#17 (“meta-analy*” OR “meta-regression” OR “meta-synthesis”). ti,ab,kw

#18 meta-analysis/

#19 17 or 18

#20 11 and 16 and 19

**EBM Reviews - Cochrane Database of Systematic Reviews via Ovid**

#1 ("2019 nCoV" or 2019nCoV or "2019 novel CoV"):ti,ab,kw

#2 ((corona next vir*) or coronavir* or (neocorona next vir*) or) neocoronavir*):ti,ab,kw

#3 COVID:ti,ab,kw

#4 COVID19:ti,ab,kw

#5 ("SARS‐CoV‐2" or "SARS‐CoV2" or SARSCoV2 or "SARSCoV‐2"):ti,ab,kw

#6 ("SARS coronavirus 2" or "SARS‐like coronavirus" or "Severe Acute Respiratory Syndrome Coronavirus‐2"):ti,ab,kw

#7 COVID-19/

#8 SARS-CoV-2/

#9 coronavirus/

#10 or/1-9

#11 exp Child/

#12 exp Adolescent/

#13 exp Infant/

#14 (child* or boy* or girl* or adolescen* or teen* or toddler* or preschooler* or pre‐schooler* or baby or babies or infant*).tw.

#15 or/11-14

#16 (“meta-analy*” OR “meta-regression” OR “meta-synthesis”). ti,ab,kw

#17 meta-analysis/

#18 16 or 17

#19 10 and 15 and 18
