# Supplementary material for: Evidence linking COVID-19 and the health/well-being of children and adolescents: an umbrella review
Source: BMC Med. 2024 Mar 13;22:116. doi: 10.1186/s12916-024-03334-x (PMC10938697; doi:10.1186/s12916-024-03334-x)
Supplement: Supplementary file 4 — Additional file 4. List of excluded studies with justification for exclusion. [file 12916_2024_3334_MOESM4_ESM.docx]

|  | **Reference** | **Reason** |
| --- | --- | --- |
|  | Anonymous (2020). "Correction to Lancet Gastroenterol Hepatol 2020; 5: 667-68 (The Lancet Gastroenterology & Hepatology (2020) 5(7) (649-657) doi: 10.1016/S2468-1253%2820%2930160-6 | Commentary |
|  | Kumar, J. (2020). "Pediatric Coronavirus Disease-19 (COVID-19): Meta-analyzing Literature Versus Natural History: Authors' Reply." Indian Pediatrics 57(9): 870 doi: 10.1007/s13312-020-1978-4 | Commentary |
|  | Cai, J., et al. (2021). "Comment on "COVID-19 infection in children: A systematic review and meta-analysis of clinical features and laboratory findings"." Archives de pediatrie: organe officiel de la Societe francaise de pediatrie. doi: 10.1016/j.arcped.2021.11.016 | Commentary |
|  | Diaz Gonzalez-Colmenero, F., et al. (2021). "Letter to the editor: "Prevalence of mental health problems among children and adolescents during the COVID-19 pandemic: A systematic review and meta-analysis"." Journal of Affective Disorders 294: 479-480. doi: 10.1016/j.jad.2021.07.069 | Commentary |
|  | Neef, V. and F. J. Raimann (2021). "Response to: Characterization of neonates born to mothers with SARS-CoV-2 infection: Review and meta-analysis." Pediatrics and Neonatology 62(6): 693. doi: 10.1016/j.pedneo.2021.08.007 | Commentary |
|  | Rogers, J. P., et al. (2021). "Erratum: Suicide, self-harm and thoughts of suicide or self-harm in infectious disease epidemics: A systematic review and meta-analysis (Journal of Physical Chemistry (2021) 30: e41 (1-2) DOI: 10.1017/S2045796021000214)." Epidemiology and Psychiatric Sciences: e41. doi: 10.1017/S2045796021000354 | Commentary |
|  | Tang, M., et al. (2021). "Comment on "Characterization of neonates born to mothers with SARS-CoV-2 infection: Review and meta-analysis"." Pediatrics and Neonatology. doi: 10.1016/j.pedneo.2021.08.003 | Commentary |
|  | Anonymous (2022). "Erratum regarding missing Declaration of Competing Interest statements in previously published articles (Clinical Epidemiology and Global Health (2021) 12, (S221339842100110X), (10.1016/j.cegh.2021.100802))." Clinical Epidemiology and Global Health 13: 100991. doi: 10.1016/j.cegh.2022.100991 | Commentary |
|  | Anonymous (2022). "Erratum: Incubation period of COVID-19 caused by unique SARS-CoV-2 strains: a systematic review and meta-analysis (JAMA Network Open (2022) 5:8 (e2228008) DOI: 10.1001/jamanetworkopen.2022.28008)." JAMA Network Open 5(9): e2235424. doi: 10.1001/jamanetworkopen.2022.35424 | Commentary |
|  | Anonymous (2022). ""Changes in alcohol use during COVID-19 and associations with contextual and individual difference variables: A systematic review and meta-analysis." Correction to Acuff et al. (2022)." Psychology of addictive behaviors: journal of the Society of Psychologists in Addictive Behaviors 36(4): 386. doi: 10.1037/adb0000852 | Commentary |
|  | Cai, J., et al. (2022). "Comment on "COVID-19 infection in children: A systematic review and meta-analysis of clinical features and laboratory findings"." Archives de Pediatrie 29(2): 157. doi: 10.1016/j.arcped.2021.11.016 | Commentary |
|  | Hasse, J. M. (2022). "Editor's note." Nutrition in Clinical Practice 37(2): 238. doi: 10.1002/ncp.10850 | Commentary |
|  | Rosenbauer, J., et al. (2022). "Comments on Rahmati et al., The global impact of COVID-19 pandemic on the incidence of pediatric new-onset type 1 diabetes and ketoacidosis: A systematic review and meta-analysis. J Med Virol. 2022; 1-16 (doi: 10.1002/jmv.27996)." Journal of Medical Virology. doi: 10.1002/jmv.28272 | Commentary |
|  | Wyckoff, M. H., et al. (2022). "2021 International consensus on cardiopulmonary resuscitation and emergency cardiovascular care science with treatment recommendations." Circulation 145(9): E645-E721. doi: 10.1161/CIR.0000000000001017 | Commentary |
|  | Cortese, S., et al. (2023). "Commentary: The impact of Covid-19 on psychopathology in children and young people worldwide - reflections on Newlove-Delgado et al. (2023)." Journal of child psychology and psychiatry, and allied disciplines. doi: 10.1111/jcpp.13765 | Commentary |
|  | Aggarwal, G., et al. (2020). "Diabetes mellitus association with coronavirus disease 2019 (COVID-19) severity and mortality: A pooled analysis." Journal of diabetes 12(11): 851-855. doi: 10.1111/1753-0407.13091 | No meta-analysis |
|  | Bradley, R., et al. (2020). "The effects of vitamin D on acute viral respiratory infections: A rapid review." Advances in Integrative Medicine 7(4): 192-202. doi: 10.1016/j.aimed.2020.07.011 | No meta-analysis |
|  | Cerro, G., et al. (2020). "CHILDHOOD-ONSET INTERSTITIAL LUNG DISEASE: A RARE CASE OF IDIOPATHIC, NONSPECIFIC INTERSTITIAL PNEUMONIA IN A TEENAGER." Chest 158(4 Supplement): A1086-A1087. doi: 10.1016/j.chest.2020.08.998 | No meta-analysis |
|  | Flisser, E., et al. (2020). "PREGNANCY LOSS RATES AFTER SINGLE, EUPLOID FROZEN-THAWED EMBRYO TRANSFER IN THE COVID-19 ERA." Fertility and Sterility 114(3 Supplement): e556-e557. doi: 10.1016/j.fertnstert.2020.09.115. doi: 10.1016/j.fertnstert.2020.09.115 | No meta-analysis |
|  | Frontera, J., et al. (2020). "Global Consortium Study of Neurological Dysfunction in COVID-19 (GCS-Neuro COVID): Study Design and Rationale." Neurocritical Care. doi: 10.1007/s12028-020-00995-3 | No meta-analysis |
|  | Geppe, N. A., et al. (2020). "Coronavirus infection in children." Voprosy Prakticheskoi Pediatrii 15(5): 73-86. doi: 10.20953/1817-7646-2020-5-73-86 | No meta-analysis |
|  | Harnett, J., et al. (2020). "The effects of Sambucus nigra berry on acute respiratory viral infections: A rapid review of clinical studies." Advances in Integrative Medicine 7(4): 240-246. doi: 10.1016/j.aimed.2020.08.001 | No meta-analysis |
|  | Islam, N., et al. (2020). "Thoracic imaging tests for the diagnosis of COVID-19." Cochrane Database of Systematic Reviews 2020(11): CD013639. doi: 10.1002/14651858.CD013639.pub3 | No meta-analysis |
|  | Jarvis, C. I., et al. (2020). "Quantifying the impact of physical distance measures on the transmission of COVID-19 in the UK." BMC Medicine 18(1): 124. doi: 10.1186/s12916-020-01597-8 | No meta-analysis |
|  | Jefferson, T., et al. (2020). "Physical interventions to interrupt or reduce the spread of respiratory viruses." The Cochrane database of systematic reviews 11: CD006207. doi: 10.1002/14651858.CD006207.pub5 | No meta-analysis |
|  | Junior, H. S., et al. (2020). "Multisystem inflammatory syndrome associated with COVID-19 from the pediatric emergency physician's point of view." Jornal de Pediatria. doi: 10.1016/j.jped.2020.08.004 | No meta-analysis |
|  | Santos, V. S., et al. (2020). "Prolonged Fecal Shedding of SARS-CoV-2 in Pediatric Patients: A Quantitative Evidence Synthesis." Journal of pediatric gastroenterology and nutrition 71(2): 150-152. doi: 10.1097/MPG.0000000000002798 | No meta-analysis |
|  | Alabbas, A., et al. (2021). "Canadian Association of Paediatric Nephrologists COVID-19 Rapid Response: Guidelines for Management of Acute Kidney Injury in Children." Canadian Journal of Kidney Health and Disease 8: 2054358121990135. doi: 10.1177/2054358121990135 | No meta-analysis |
|  | Anonymous (2021). "The Annual Assembly of Hospice and Palliative Care." Journal of Pain and Symptom Management 61(3): A1-A10. doi: 10.1016/s0885-3924%2821%2900013-0 | No meta-analysis |
|  | "Psychiatry Update 2021 Spring Abstract." Annals of Clinical Psychiatry 33(3 SUPPL). | No meta-analysis |
|  | "2021 Advances in Inflammatory Bowel Diseases Annual Meeting." American Journal of Gastroenterology 116(SUPPL). | No meta-analysis |
|  | Aurini, J. and S. Davies (2021). "COVID-19 school closures and educational achievement gaps in Canada: Lessons from Ontario summer learning research." Canadian review of sociology: Revue canadienne de sociologie 58(2): 165-185. doi: 10.1111/cars.12334 | No meta-analysis |
|  | Axfors, C., et al. (2021). "Mortality outcomes with hydroxychloroquine and chloroquine in COVID-19 from an international collaborative meta-analysis of randomized trials." Nature Communications 12(1): 2349. doi: 10.1038/s41467-021-22446-z | No meta-analysis |
|  | Barnett, P., et al. (2021). "Implementation of telemental health services before COVID-19: Rapid umbrella review of systematic reviews." Journal of Medical Internet Research 23(7): e26492. doi: 10.2196/26492 | No meta-analysis |
|  | Carretta, D. M., et al. (2021). "Cardiac involvement in COVID-19 patients: A contemporary review." Infectious Disease Reports 13(2): 494-517. doi: 10.3390/idr13020048 | No meta-analysis |
|  | Chagla, Z. (2021). "In patients with COVID-19, viral shedding time is 17 d and varies in some subgroups." Annals of Internal Medicine 174(8): JC94. doi: 10.7326/ACPJ202108170-094 | No meta-analysis |
|  | Chen, Y., et al. (2021). "Covid-19 associated acute chest syndrome in children with sickle cell disease." Journal of Investigative Medicine 69(2): 550. doi: 10.1136/jim-2021-SRMC.331 | No meta-analysis |
|  | Chevret, S. (2021). "Challenges of using historical data in clinical trials." Revue d'Epidemiologie et de Sante Publique 69(Supplement 1): S12-S13. doi: 10.1016/j.respe.2021.04.017 | No meta-analysis |
|  | Chuey, A., et al. (2021). "Moderated Online Data-Collection for Developmental Research: Methods and Replications." Frontiers in psychology 12: 734398. doi: 10.3389/fpsyg.2021.734398 | No meta-analysis |
|  | Correale, C., et al. (2021). "Pediatric patients with epilepsy showed elevate rate of anxious depressive symptoms during COVID-19 pandemic: Preliminary findings of monocentric a cross-sectional study." Epilepsia 62(SUPPL 3): 319. doi: 10.1111/epi.17079 | No meta-analysis |
|  | Dinnes, J., et al. (2021). "Rapid, point-of-care antigen and molecular-based tests for diagnosis of SARS-CoV-2 infection." The Cochrane database of systematic reviews **3**: CD013705. doi: 10.1002/14651858.CD013705 | No meta-analysis |
|  | Dioguardi, M., et al. (2021). "Innate Immunity in Children and the Role of ACE2 Expression in SARS-CoV-2 Infection." Pediatric Reports 13(3): 363-382. doi: 10.3390/pediatric13030045 | No meta-analysis |
|  | Edelson, S. and V. Reyna (2021). "How Fuzzy-trace Theory Predicts Development of Risky Decision Making, with Novel Extensions to Culture and Reward Sensitivity." Developmental review: DR 62. doi: 10.1016/j.dr.2021.100986 | No meta-analysis |
|  | Giurge, L. M., et al. (2021). "A multicountry perspective on gender differences in time use during COVID-19." Proceedings of the National Academy of Sciences of the United States of America 118(12): e2018494118. doi: 10.1073/pnas.2018494118 | No meta-analysis |
|  | Hatmi, Z. N. (2021). "A Systematic Review of Systematic Reviews on the COVID-19 Pandemic." SN Comprehensive Clinical Medicine 3(2): 419-436. doi: 10.1007/s42399-021-00749-y | No meta-analysis |
|  | Heidary, F., et al. (2021). "Acyclovir as a potential add-on therapy in COVID-19 treatment regimens." Pharmaceutical Sciences 27: S68-S77. doi: 10.34172/PS.2021.38 | No meta-analysis |
|  | Jachvadze, M., et al. (2021). "INFLUENCE OF VITAMIN D ON HUMAN HEALTH (REVIEW)." Georgian medical news (321): 36-41. | No meta-analysis |
|  | Liu, C. Y., et al. (2021). "Rapid Review of Social Contact Patterns during the COVID-19 Pandemic." Epidemiology 32(6): 781-791. doi: 10.1097/EDE.0000000000001412 | No meta-analysis |
|  | Ludvigsson, J. F. (2021). "Case report and systematic review suggest that children may experience similar long-term effects to adults after clinical COVID-19." Acta Paediatrica, International Journal of Paediatrics 110(3): 914-921. doi: 10.1111/apa.15673 | No meta-analysis |
|  | Mirzaei, F. (2021). "How to manage internet addiction and prevent from social pain among students?" Journal of Pain Management 14(1): 29-31. | No meta-analysis |
|  | Mohaghegh, S., et al. (2021). "Role of ACE2 polymorphism in COVID-19: impact of age." Clinical Chemistry and Laboratory Medicine. doi: 10.1515/cclm-2020-1877 | No meta-analysis |
|  | Morina, N., et al. (2021). "Potential impact of physical distancing on physical and mental health: A rapid narrative umbrella review of meta-analyses on the link between social connection and health." BMJ Open 11(3): e042335. doi: 10.1136/bmjopen-2020-042335 | No meta-analysis |
|  | Murphy Jones, L. and P. Khatri (2021). "Multisystem inflammatory syndrome in children: a microcosm of challenges and opportunities for translational bioinformatics in pediatric research." Current opinion in pediatrics 33(3): 325-330. doi: 10.1097/MOP.0000000000001012 | No meta-analysis |
|  | Nicastro, E., et al. (2021). "COVID-19 in Immunosuppressed Children." Frontiers in Pediatrics 9: 629240. doi: 10.3389/fped.2021.629240 | No meta-analysis |
|  | Oto, B. B., et al. (2021). "Shall we change our attitude in screening for retinopathy of prematurity during the COVID-19 pandemic?" Ophthalmologica 244(SUPPL 1). | No meta-analysis |
|  | Patra, P. K., et al. (2021). "Non-SARS, non-MERS human coronavirus infections and risk of Kawasaki disease: a meta-analysis." Future Virology. doi: 10.2217/fvl-2021-0176 | No meta-analysis |
|  | Pfeiffer, A. F., et al. (2021). "The deleterious effects of COVID-19 in the peripartum period: A case report." Pediatric Reports 13(2): 334-339. doi: 10.3390/pediatric13020041 | No meta-analysis |
|  | Pires, C. (2021). "What is the state-of-the-art in clinical trials on vaccine hesitancy 2015-2020?" Vaccines 9(4): 348. doi: 10.3390/vaccines9040348 | No meta-analysis |
|  | Pourfridoni, M., et al. (2021). "Fluid and Electrolyte Disturbances in COVID-19 and Their Complications." BioMed Research International 2021: 6667047. doi: 10.1155/2021/6667047 | No meta-analysis |
|  | Rahman, A., et al. (2021). "20.1 IMPROVING ACCESS TO PSYCHOSOCIAL INTERVENTIONS FOR PERINATAL DEPRESSION IN LOW- AND MIDDLE-INCOME COUNTRIES: LESSONS FROM THE FIELD." Journal of the American Academy of Child and Adolescent Psychiatry 60(10 Supplement): S288. doi: 10.1016/j.jaac.2021.07.667 | No meta-analysis |
|  | Rajput, S., et al. (2021). "COVID-19 and Gut Microbiota: A Potential Connection." Indian Journal of Clinical Biochemistry 36(3): 266-277. doi: 10.1007/s12291-020-00948-9 | No meta-analysis |
|  | Robaina-Castellanos, G. R. and S. d. l. C. Riesgo-Rodriguez (2021). "Congenital and Intrapartum SARS-CoV-2 Infection in Neonates: Hypotheses, Evidence and Perspectives." MEDICC Review 23(1): 72-83. doi: 10.37757/MR2021.V23. N1.13 | No meta-analysis |
|  | Saha, S. and S. Saha (2021). "Epidemiological burden of parents being the index cases of COVID-19 infected children." World journal of methodology 11(1): 1-14. doi: 10.5662/wjm. v11. i1.1 | No meta-analysis |
|  | Samanta, D., et al. (2021). "Conceptualization and implementation of an interdisciplinary clinic for children with drug-resistant epilepsy during the COVID-19 pandemic." Epilepsy and Behavior 125: 108403. doi: 10.1016/j.yebeh.2021.108403 | No meta-analysis |
|  | Samuel, R., et al. (2021). "Deliberate self-harm among children and young people at a large secondary care centre." Archives of Disease in Childhood 106(SUPPL 1): A192-A193. doi: 10.1136/archdischild-2021-rcpch.335 | No meta-analysis |
|  | Simon Junior, H., et al. (2021). "Multisystem inflammatory syndrome associated with COVID-19 from the pediatric emergency physician's point of view." Jornal de Pediatria 97(2): 140-159. doi: 10.1016/j.jped.2020.08.004 | No meta-analysis |
|  | Sudre, C. H., et al. (2021). "Anosmia, ageusia, and other COVID-19-like symptoms in association with a positive SARS-CoV-2 test, across six national digital surveillance platforms: an observational study." The Lancet. Digital health 3(9): e577-e586. doi: 10.1016/S2589-7500(21)00115-1 | No meta-analysis |
|  | The Lancet Global, H. (2021). "Progressing the investment case in maternal and child health." The Lancet Global Health 9(5): e558. doi: 10.1016/S2214-109X%2821%2900178-9 | No meta-analysis |
|  | Trahan, M. J., et al. (2021). "364 Obstetrical and neonatal outcomes among pregnancies with SARS-CoV-2." American Journal of Obstetrics and Gynecology 224(2 Supplement): S237. doi: 10.1016/j.ajog.2020.12.385 | No meta-analysis |
|  | van Veenendaal, N. R., et al. (2021). "Supporting parents as essential care partners in neonatal units during the SARS-CoV-2 pandemic." Acta Paediatrica, International Journal of Paediatrics 110(7): 2008-2022. doi: 10.1111/apa.15857 | No meta-analysis |
|  | Walkup, J. T. (2021). "5.2 PSYCHOSIS WORKUP, MEASUREMENT-BASED CARE, MEDICATION SAFETY, DEEP BRAIN STIMULATION, INSOMNIA, AND YOUTH AND COVID-19." Journal of the American Academy of Child and Adolescent Psychiatry 60(10 Supplement): S130. doi: 10.1016/j.jaac.2021.07.519 | No meta-analysis |
|  | Walsh, K., et al. (2021). "Narrative review: COVID-19 and pediatric anxiety." Journal of Psychiatric Research 144: 421-426. doi: 10.1016/j.jpsychires.2021.10.013 | No meta-analysis |
|  | Weber, G. M., et al. (2021). "International changes in COVID-19 clinical trajectories across 315 hospitals and 6 countries: Retrospective cohort study." Journal of Medical Internet Research 23(10): e31400. doi: 10.2196/31400 | No meta-analysis |
|  | Zaccari, V., et al. (2021). "Narrative Review of COVID-19 Impact on Obsessive-Compulsive Disorder in Child, Adolescent and Adult Clinical Populations." Frontiers in Psychiatry 12: 673161. doi: 10.3389/fpsyt.2021.673161 | No meta-analysis |
|  | Zhou, C., et al. (2021). "Laboratory parameters between multisystem inflammatory syndrome in children and Kawasaki disease." Pediatric Pulmonology 56(12): 3688-3698. doi: 10.1002/ppul.25687 | No meta-analysis |
|  | Abbasi, M. A., et al. (2022). "Efficacy of Dexamethasone and Methylprednisolone in Hospitalization Outcomes of COVID-19 Patients: A Comparative Retrospective Study." Archives of Clinical Infectious Diseases 17(2): e129727. doi: 10.5812/archcid-129727 | No meta-analysis |
|  | "9. Gemeinsame Jahrestagung der Deutschen Gesellschaft fur Neurorehabilitation e. V. und der Deutschen Gesellschaft fur Neurotraumatologie und Klinische Neurorehabilitation e. V." Neurologie und Rehabilitation 28(Supplement 1). | No meta-analysis |
|  | "Scrubs (QUB Surgical Society) Medical Students' Academic Medicine Conference and Research Symposium." Ulster Medical Journal 91(3). | No meta-analysis |
|  | "12th International Symposium on Hodgkin Lymphoma." HemaSphere 6(Supplement 5). | No meta-analysis |
|  | Borel, M., et al. (2022). "Long-term physical, mental and social health effects of COVID-19 in the pediatric population: a scoping review." World Journal of Pediatrics 18(3): 149-159. doi: 10.1007/s12519-022-00515-7 | No meta-analysis |
|  | Dauletbaev, N., et al. (2022). "A scoping review of mHealth monitoring of pediatric bronchial asthma before and during COVID-19 pandemic." Paediatric respiratory reviews 43: 67-77. doi: 10.1016/j.prrv.2022.01.002 | No meta-analysis |
|  | Estes, A. and D. Macariola (2022). "A Clinical Differentiation of Multi system Inflammatory Syndrome in Children (MISC) & Kawasaki Disease (KD)." Pediatrics 149. | No meta-analysis |
|  | Gao, C. X., et al. (2022). "Inequalities in Access to Mental Health Treatment by Australian Youths During the COVID-19 Pandemic." Psychiatric services (Washington, D.C.): appips20220345. doi: 10.1176/appi.ps.20220345 | No meta-analysis |
|  | Godsey, C., et al. (2022). "A collaborative, retrospective study of pediatric critical illness in wisconsin during COVID-19." Critical Care Medicine 50(1 SUPPL): 63. doi: 10.1097/01.ccm.0000806960.55637.4a | No meta-analysis |
|  | Lee, J. Y., et al. (2022). "3.129 The Effect of Mobile Digital Interventions on Mental Health Measures During the COVID-19 Pandemic: A Systematic Review of Controlled Studies." Journal of the American Academy of Child and Adolescent Psychiatry 61(10 Supplement): S270. doi: 10.1016/j.jaac.2022.09.407 | No meta-analysis |
|  | Li, J., et al. (2022). "Zinc Intakes and Health Outcomes: An Umbrella Review." Frontiers in nutrition 9: 798078. doi: 10.3389/fnut.2022.798078 | No meta-analysis |
|  | Martin Delawalla, M. L., et al. (2022). "182. The Impact of the COVID-19 Pandemic on Adolescent Social Media Use, Substance Use, and Depressive Symptoms: A Scoping Review." Journal of Adolescent Health 70(4 Supplement): S95. doi: 10.3389/fnut.2022.798078 | No meta-analysis |
|  | Nzoumbou-Boko, R., et al. (2022). "Malaria research in the Central African Republic from 1987 to 2020: an overview." Tropical Medicine and Health 50(1): 70. doi: 10.1186/s41182-022-00446-z | No meta-analysis |
|  | Oduwole, E. O., et al. (2022). "Overview of Tools and Measures Investigating Vaccine Hesitancy in a Ten Year Period: A Scoping Review." Vaccines 10(8): 1198. doi: 10.3390/vaccines10081198 | No meta-analysis |
|  | Ougrin, D., et al. (2022). "Pandemic-related emergency psychiatric presentations for self-harm of children and adolescents in 10 countries (PREP-kids): a retrospective international cohort study." European Child and Adolescent Psychiatry 31(7): 1-13. doi: 10.1007/s00787-021-01741-6 | No meta-analysis |
|  | Perski, O., et al. (2022). "Interventions to increase personal protective behaviours to limit the spread of respiratory viruses: A rapid evidence review and meta-analysis." British journal of health psychology 27(1): 215-264. doi: 10.1111/bjhp.12542 | No meta-analysis |
|  | Petrovic, T., et al. (2022). "IgG N-glycome changes during the course of severe COVID-19: An observational study." eBioMedicine 81: 104101. doi: 10.1016/j.ebiom.2022.104101 | No meta-analysis |
|  | Prentice, A. M. (2022). "Breastfeeding in the Modern World." Annals of Nutrition and Metabolism 78(Supplement 2): 29-38. doi: 10.1159/000524354 | No meta-analysis |
|  | Siemens, W., et al. (2022). "Three out of four published systematic reviews on COVID-19 treatments were not registered and one-third of those registered were published: a meta-research study." Journal of Clinical Epidemiology 152: 36-46. doi: 10.1016/j.jclinepi.2022.09.011 | No meta-analysis |
|  | Staub, H. L. and L. P. Staub (2022). "Post-COVID-19 multisystem inflammatory syndrome: An evolving concept." Scientia Medica 32(1): e-42436. doi: 10.15448/1980-6108.2022.1.42436 | No meta-analysis |
|  | Summerlin, J., et al. (2022). "A Review of Current and Emerging Therapeutic Options for Hemophagocytic Lymphohistiocytosis." Annals of Pharmacotherapy. doi: 10.1177/10600280221134719 | No meta-analysis |
|  | Watcharapalakorn, A., et al. (2022). "Coronavirus disease 2019 outbreak and associated public health measures increase the progression of myopia among children and adolescents: Evidence synthesis." Ophthalmic & physiological optics: the journal of the British College of Ophthalmic Opticians (Optometrists) 42(4): 744-752. doi: 10.1111/opo.12976 | No meta-analysis |
|  | Wijaya, J. T., et al. (2022). "RISK FACTORS FOR COVID-19 MORTALITY IN PAEDIATRIC POPULATIONS: A SCOPING REVIEW." Archives of Disease in Childhood 107(Supplement 2): A207. doi: 10.1136/archdischild-2022-rcpch.332 | No meta-analysis |
|  | Braga, P. P., et al. (2023). "Children wearing face masks to prevent communicable diseases: scoping review." Revista Paulista de Pediatria 41: e2021164. doi: 10.1590/1984-0462/2023/41/2021164 | No meta-analysis |
|  | Fazaludeen Koya, S., et al. (2023). "Vector-Borne and Zoonotic Diseases in the Eastern Mediterranean Region: A Systematic Review." Journal of Epidemiology and Global Health. doi: 10.1007/s44197-023-00091-7 | No meta-analysis |
|  | Rusconi, F., et al. (2023). "Pregnancy outcomes in Italy during COVID-19 pandemic: A population-based cohort study." BJOG: An International Journal of Obstetrics and Gynaecology 130(3): 276-284. doi: 10.1111/1471-0528.17315 | No meta-analysis |
|  | Shukla, S., et al. (2023). "Association between public health emergencies and sexual and reproductive health, gender-based violence, and early marriage among adolescent girls: a rapid review." BMC public health 23(1): 117. doi: 10.1186/s12889-023-15054-7 | No meta-analysis |
|  | Tope, P., et al. (2023). "The impact of lag time to cancer diagnosis and treatment on clinical outcomes prior to the COVID-19 pandemic: a scoping review of systematic reviews and meta-analyses." eLife 12: e81354. doi: 10.7554/eLife.81354 | No meta-analysis |
|  | Bai, H., Ji, Y., Wang, J., and Zhang, X. (2020). Efficacy of human coronavirus immune convalescent plasma for the treatment of corona virus disease -19 disease in hospitalized children: A protocol for systematic review and meta-analysis. Medicine 99, e22017. doi: 10.1097/MD.0000000000022017 | No meta-analysis |
|  | Bi, L., Li, Y., Hu, X., Wang, Q., Liang, X., Yu, X., Dong, L., and Xie, Q. (2020). The effectiveness and safety of traditional Chinese medicine for the treatment of children with COVID-19. Medicine 99, e21247. doi: 10.1097/MD.0000000000021247 | No meta-analysis |
|  | Dambha-Miller, H., Albasri, A., Hodgson, S., Wilcox, C., Islam, N., Khan, S., Little, P., and Griffin, S. (2020). Drug treatments affecting ACE2 in COVID-19 infection: A systematic review protocol. BJGP Open 4, bjgpopen20X101115. doi: 10.3399/bjgpopen20X101115 | No meta-analysis |
|  | Medeiros, K. S., Sarmento, A. C. A., Martins, E. S., Costa, A. P. F., Eleuterio, J., Jr., and Goncalves, A. K. (2020). Impact of SARS-CoV-2 (COVID-19) on pregnancy: a systematic review and meta-analysis protocol. BMJ open 10, e039933. doi: 10.1136/bmjopen-2020-039933 | No meta-analysis |
|  | Silva Junior, F. J. G. d., Sales, J. C. E. S., Monteiro, C. F. d. S., Costa, A. P. C., Campos, L. R. B., Miranda, P. I. G., Monteiro, T. A. d. S., Lima, R. A. G., and Lopes-Junior, L. C. (2020). Impact of COVID-19 pandemic on mental health of young people and adults: a systematic review protocol of observational studies. BMJ open 10, e039426. doi: 10.1136/bmjopen-2020-039426 | No meta-analysis |
|  | Smith, E. R., Oakley, E., He, S., Zavala, R., Ferguson, K., Miller, L., Grandner, G. W., Abejirinde, I. O. O., Afshar, Y., Ahmadzia, H., Aldrovandi, G., Akelo, V., Tippett Barr, B. A., Bevilacqua, E., Brandt, J. S., Broutet, N., Fernandez-Buhigas, I., Carrillo, J., Clifton, R., Conry, J., Cosmi, E., Delgado-Lopez, C., Divakar, H., Driscoll, A. J., Favre, G., Flaherman, V., Gale, C., Gil, M. M., Godwin, C., Gottlieb, S., Bellolio, O. H., Kara, E., Khagayi, S., Kim, C. R., Knight, M., Kotloff, K., Lanzone, A., Le Doare, K., Lees, C., Litman, E., Lokken, E. M., Longo, V. L., Magee, L. A., Martinez-Portilla, R. J., McClure, E., Metz, T. D., Money, D., Mullins, E., Nachega, J. B., Panchaud, A., Playle, R., Poon, L. C., Raiten, D., Regan, L., Rukundo, G., Sanin-Blair, J., Temmerman, M., Thorson, A., Thwin, S. S., Tolosa, J. E., Townson, J., Valencia-Prado, M., Visentin, S., von Dadelszen, P., Waldorf, K. A., Whitehead, C., Yang, H., Thorlund, K., and Tielsch, J. M. (2020). Protocol for a sequential, prospective meta-analysis to describe coronavirus disease 2019 (COVID-19) in the pregnancy and postpartum periods. medRxiv. doi: 10.1101/2020.11.08.20228056 | No meta-analysis |
|  | Bell, Z., Scott, S., Visram, S., Rankin, J., Bambra, C., and Heslehurst, N. (2021). Food insecurity and the nutritional health and well-being of women and children in high-income countries: Protocol for a qualitative systematic review. BMJ Open 11, e048180. doi: 10.1136/bmjopen-2020-048180 | No meta-analysis |
|  | Dimala, C. A., Kadia, B. M., Nguyen, H., and Donato, A. (2021). Community and provider acceptability of the COVID19 vaccine: A systematic review and meta-analysis protocol. Journal of General Internal Medicine 36, S59. doi: 10.1007/s11606-021-06830-5 | No meta-analysis |
|  | Mulder, I. A., Huntley, B., Di Mascio, D., Berghella, V., and Chauhan, S. P. (2021). 797 Adverse outcomes among individuals with and without SARS-CoV-2 infection: a systematic review and meta-analysis. American Journal of Obstetrics and Gynecology 224, S496-S497. dx.doi: 10.1016/j.ajog.2020.12.820 | No meta-analysis |
|  | Tang, Z., Li, M., Chen, W., Ran, X., Li, H., and Chen, Z. (2021). Clinical symptoms of COVID-19 pneumonia in children: A protocol for systematic review and meta-analysis. Medicine 100, e24108. doi: 10.1097/MD.0000000000024108 | No meta-analysis |
|  | Osborne, B., Moorjani-Houle, M., Fakhraei, R., Walker, M., Wen, S. W., and Guo, Y. (2022). Impact of COVID-19 pandemic on emergency department visits and infant health: a scoping review protocol. BMJ Open 12, e061778. doi: 10.1136/bmjopen-2022-061778 | No meta-analysis |
|  | Rajan, R., Kshatriya, M., Banfield, L., Athale, U., Thabane, L., and Constantine Samaan, M. (2022). Impact of virtual care on health-related quality of life in children with diabetes mellitus: A systematic review protocol. BMJ Open 12, e053642. doi: 10.1136/bmjopen-2021-053642 | No meta-analysis |
|  | Smith, E. R., Oakley, E., He, S., Zavala, R., Ferguson, K., Miller, L., Grandner, G. W., Abejirinde, I. O. O., Afshar, Y., Ahmadzia, H., Aldrovandi, G., Akelo, V., Tippett Barr, B. A., Bevilacqua, E., Brandt, J. S., Broutet, N., Buhigas, I. F., Carrillo, J., Clifton, R., Conry, J., Cosmi, E., Delgado-Lopez, C., Divakar, H., Driscoll, A. J., Favre, G., Flaherman, V., Gale, C., Gil, M. M., Godwin, C., Gottlieb, S., Bellolio, O. H., Kara, E., Khagayi, S., Kim, C. R., Knight, M., Kotloff, K., Lanzone, A., Le Doare, K., Lees, C., Litman, E., Lokken, E. M., Longo, V. L., Magee, L. A., Martinez-Portilla, R. J., McClure, E., Metz, T. D., Money, D., Mullins, E., Nachega, J. B., Panchaud, A., Playle, R., Poon, L. C., Raiten, D., Regan, L., Rukundo, G., Sanin-Blair, J., Temmerman, M., Thorson, A., Thwin, S., Tolosa, J. E., Townson, J., Valencia-Prado, M., Visentin, S., Von Dadelszen, P., Waldorf, K. A., Whitehead, C., Yang, H., Thorlund, K., and Tielsch, J. M. (2022). Protocol for a sequential, prospective metaanalysis to describe coronavirus disease 2019 (COVID-19) in the pregnancy and postpartum periods. PLoS ONE 17, e0270150. doi: 10.1371/journal.pone.0270150 | No meta-analysis |
|  | Soheili, M., Moradi, G., Baradaran, H. R., Soheili, M., Mokhtari, M. M., and Moradi, Y. (2022). Clinical manifestation and maternal complications and neonatal outcomes in pregnant women with COVID-19: a comprehensive evidence synthesis and meta-analysis. The journal of maternal-fetal & neonatal medicine: the official journal of the European Association of Perinatal Medicine, the Federation of Asia and Oceania Perinatal Societies, the International Society of Perinatal Obstetricians 35, 5672-5685. doi: 10.1080/14767058.2021.1888923 | No meta-analysis |
|  | Charide, R., Stallwood, L., Munan, M., Sayfi, S., Hartling, L., Butcher, N. J., Offringa, M., Elliott, S., Richards, D. P., Mathew, J. L., Akl, E. A., Kredo, T., Mbuagbaw, L., Motillal, A., Baba, A., Prebeg, M., Relihan, J., Scott, S. D., Suvada, J., Falavigna, M., Klugar, M., Lotfi, T., Stevens, A., Pottie, K., and Schunemann, H. J. (2023). Knowledge mobilization activities to support decision-making by youth, parents, and adults using a systematic and living map of evidence and recommendations on COVID-19: protocol for three randomized controlled trials and qualitative user-experience studies. Trials 24, 27. doi: 10.1186/s13063-023-07067-9 | No meta-analysis |
|  | Alsaied, T. (2020). "From Other Journals: A Review of Recent Articles in Pediatric Cardiology." Pediatric Cardiology 41(7): 1532-1537. doi: 10.1007/s00246-020-02438-4 | Not a Systematic Review |
|  | Green, M. S., et al. (2020). "The confounded crude case-fatality rates (CFR) for COVID-19 hide more than they reveal-a comparison of age-specific and age-adjusted CFRs between seven countries." PLoS ONE 15(10): e0241031. doi: 10.1371/journal.pone.0241031 | Not a Systematic Review |
|  | "Abstracts From the 2021 Crohn's & ColitisCongress held January 21-24, 2021." Gastroenterology 160(3 Supplement): 1-20. | Not a Systematic Review |
|  | Chen, P. Z., et al. (2021). "Heterogeneity in transmissibility and shedding SARS-CoV-2 via droplets and aerosols." eLife 10. doi: 10.7554/eLife.65774 | Not a Systematic Review |
|  | Conrady, B., et al. (2021). "Cryptosporidium spp. Infections in Combination with Other Enteric Pathogens in the Global Calf Population." Animals: an open access journal from MDPI 11(6). doi: 10.3390/ani11061786 | Not a Systematic Review |
|  | Dyussenova, S. B., et al. (2021). "The role of Vitamin D in respiratory viral infections and other infectious diseases." Open Access Macedonian Journal of Medical Sciences 9: 1109-1114. doi: 10.3889/oamjms.2021.6992 | Not a Systematic Review |
|  | Macdonald, J. A., et al. (2021). "Cohort profile: The Men and Parenting Pathways (MAPP) Study: A longitudinal Australian cohort study of men's mental health and well-being at the normative age for first-time fatherhood." BMJ Open 11(7): e047909. doi: 10.1136/bmjopen-2020-047909 | Not a Systematic Review |
|  | Yip, T. (2021). "6.2 ETHNIC/RACIAL IDENTITY AND DISCRIMINATION: IMPLICATIONS FOR ADOLESCENT AND YOUNG ADULT DEVELOPMENT." Journal of the American Academy of Child and Adolescent Psychiatry 60(10 Supplement): S132. doi: 10.1016/j.jaac.2021.07.527 | Not a Systematic Review |
|  | Moore, D. P. (2022). "DEVELOPING VACCINES TO COMBAT HUMAN DISEASES: A HISTORY." Current Allergy and Clinical Immunology 35(1): 6-15. doi: 10.1016/j.jadohealth.2022.01.099 | Not a Systematic Review |
|  | Piras, A., et al. (2022). "Myopericarditis in a Male Adolescent Following the Second Shot of COVID-19 Pfizer m-RNA Vaccine: Typical Example and Analysis of 110 Single Case Reports." Pediatric Reports 14(4): 401-409. doi: 10.3390/pediatric14040048 | Not a Systematic Review |
|  | Borchering, R. K., et al. (2023). "Impact of SARS-CoV-2 vaccination of children ages 5-11 years on COVID-19 disease burden and resilience to new variants in the United States, November 2021-March 2022: A multi-model study." Lancet regional health. Americas 17: 100398. doi: 10.1016/j.lana.2022.100398 | Not a Systematic Review |
|  | Abdo, C., et al. (2020). "Domestic violence and substance abuse during COVID19: A systematic review." Indian Journal of Psychiatry 62(Suppl 3): S337-S342. doi: 10.4103/psychiatry.IndianJPsychiatry_1049_20 | Unrelated |
|  | Allotey, J., et al. (2020). "Clinical manifestations, risk factors, and maternal and perinatal outcomes of coronavirus disease 2019 in pregnancy: living systematic review and meta-analysis." BMJ (Clinical research ed.) 370: m3320. doi: 10.1136/bmj.m3320 | Unrelated |
|  | Anastassopoulou, C., et al. (2020). "Human genetic factors associated with susceptibility to SARS-CoV-2 infection and COVID-19 disease severity." Human Genomics 14(1): 40. doi: 10.1186/s40246-020-00290-4 | Unrelated |
|  | Banerjee, D., et al. (2020). "Impact of the COVID-19 pandemic on psychosocial health and well-being in South-Asian (World Psychiatric Association zone 16) countries: A systematic and advocacy review from the Indian Psychiatric Society." Indian Journal of Psychiatry 62(9 Supplement 3): S343-S353. doi: 10.4103/psychiatry.IndianJPsychiatry_1002_20 | Unrelated |
|  | Baradaran, A., et al. (2020). "COVID-19 associated multisystem inflammatory syndrome: A systematic review and meta-analysis." Iranian Journal of Allergy, Asthma and Immunology 19(6): 570-588. doi: 10.18502/ijaai.v19i6.4927 | Unrelated |
|  | Byambasuren, O., et al. (2020). "Estimating the extent of asymptomatic COVID-19 and its potential for community transmission: Systematic review and meta-analysis." Journal of the Association of Medical Microbiology and Infectious Disease Canada 5(4): 223-234. doi: 10.3138/jammi-2020-0030 | Unrelated |
|  | Cao, K., et al. (2020). "Current evidence of 2019 novel coronavirus disease (CoVID-19) ocular transmission: A systematic review and meta-analysis." BioMed Research International 2020: 7605453. doi: 10.1155/2020/7605453 | Unrelated |
|  | Capobianco, G., et al. (2020). "COVID-19 in pregnant women: A systematic review and meta-analysis." European Journal of Obstetrics and Gynecology and Reproductive Biology 252: 543-558. doi: 10.1016/j.ejogrb.2020.07.006 | Unrelated |
|  | Chai, K. L., et al. (2020). "Convalescent plasma or hyperimmune immunoglobulin for people with COVID-19: a living systematic review." The Cochrane database of systematic reviews 10: CD013600. doi: 10.1002/14651858.CD013600.pub3 | Unrelated |
|  | da Silva, F. A. F., et al. (2020). "Covid-19 gastrointestinal manifestations: A systematic review." Revista da Sociedade Brasileira de Medicina Tropical 53: 1-11. doi: 10.1590/0037-8682-0714-2020 | Unrelated |
|  | Das, R. R., et al. (2020). "Effect of chloroquine and hydroxychloroquine on COVID-19 virological outcomes: An updated meta-analysis." Indian journal of medical microbiology 38(3 & 4): 265-272. doi: 10.4103/ijmm.IJMM_20_330 | Unrelated |
|  | Di Mascio, D., et al. (2020). "Outcome of coronavirus spectrum infections (SARS, MERS, COVID-19) during pregnancy: a systematic review and meta-analysis." American journal of obstetrics & gynecology MFM 2(2): 100107. doi: 10.1016/j.ajogmf.2020.100107 | Unrelated |
|  | Di Toro, F., et al. (2020). "Impact of COVID-19 on maternal and neonatal outcomes: a systematic review and meta-analysis." Clinical microbiology and infection: the official publication of the European Society of Clinical Microbiology and Infectious Diseases. doi: 10.1016/j.cmi.2020.10.007 | Unrelated |
|  | Doyle, F. L. and L. Klein (2020). "Postnatal Depression Risk Factors: An Overview of Reviews to Inform COVID-19 Research, Clinical, and Policy Priorities." Frontiers in global women's health 1: 577273. doi: 10.3389/fgwh.2020.577273 | Unrelated |
|  | Elhabyan, A., et al. (2020). "The role of host genetics in susceptibility to severe viral infections in humans and insights into host genetics of severe COVID-19: A systematic review." Virus research 289: 198163. doi: 10.1016/j.virusres.2020.198163 | Unrelated |
|  | Elshazli, R. M., et al. (2020). "Diagnostic and prognostic value of hematological and immunological markers in COVID-19 infection: A meta-analysis of 6320 patients." PLoS ONE 15(8): e0238160. doi: 10.1371/journal.pone.0238160 | Unrelated |
|  | Gao, Y., et al. (2020). "Impacts of immunosuppression and immunodeficiency on COVID-19: A systematic review and meta-analysis." The Journal of infection 81(2): e93-e95. doi: 10.1016/j.jinf.2020.05.017 | Unrelated |
|  | Gao, Y.-J., et al. (2020). "Clinical features and outcomes of pregnant women with COVID-19: a systematic review and meta-analysis." BMC Infectious Diseases 20(1): 564. doi: 10.1186/s12879-020-05274-2 | Unrelated |
|  | Garg, M., et al. (2020). "Diagnostic accuracy of CT and radiographic findings for novel coronavirus 2019 pneumonia: Systematic review and meta-analysis." Clinical imaging 72: 75-82. doi: 10.1016/j.clinimag.2020.11.021 | Unrelated |
|  | Gordon, M., et al. (2020). "Rapid systematic review of neonatal COVID-19 including a case of presumed vertical transmission." BMJ Paediatrics Open 4(1): 000718. doi: 10.1136/bmjpo-2020-000718 | Unrelated |
|  | Hasani, H., et al. (2020). "The Novel Coronavirus Disease (COVID-19): A PRISMA Systematic Review and Meta-Analysis of Clinical and Paraclinical Characteristics." BioMed Research International 2020: 3149020. doi: 10.1155/2020/3149020 | Unrelated |
|  | Hassanipour, S., et al. (2020). "A systematic review and meta-analysis of pregnancy and covid-19: Signs and symptoms, laboratory tests, and perinatal outcomes." International Journal of Reproductive BioMedicine 18(12): 1005-1018. doi: 10.18502/ijrm.v18i12.8022 | Unrelated |
|  | He, J., et al. (2020). "Proportion of asymptomatic coronavirus disease 2019 (COVID-19): a systematic review and meta-analysis." Journal of Medical Virology. doi: 10.1002/jmv.26326 | Unrelated |
|  | Jutzeler, C. R., et al. (2020). "Comorbidities, clinical signs and symptoms, laboratory findings, imaging features, treatment strategies, and outcomes in adult and pediatric patients with COVID-19: A systematic review and meta-analysis." Travel medicine and infectious disease: 101825. doi: 10.1016/j.tmaid.2020.101825 | Unrelated |
|  | Kadir, R. A., et al. (2020). "COVID-19 coagulopathy in pregnancy: Critical review, preliminary recommendations, and ISTH registry-Communication from the ISTH SSC for Women's Health." Journal of thrombosis and haemostasis : JTH 18(11): 3086-3098. doi: 10.1111/jth.15072 | Unrelated |
|  | Kashi, A. H., et al. (2020). "Urinary Viral Shedding of COVID-19 and its Clinical Associations: A Systematic Review and Meta-analysis of Observational Studies." Urology journal 17(5): 433-441. doi: 10.22037/uj.v16i7.6248 | Unrelated |
|  | Kasraeian, M., et al. (2020). "COVID-19 pneumonia and pregnancy; a systematic review and meta-analysis." The journal of maternal-fetal & neonatal medicine: the official journal of the European Association of Perinatal Medicine, the Federation of Asia and Oceania Perinatal Societies, the International Society of Perinatal Obstetricians: 1-8. doi: 10.1080/14767058.2020.1763952 | Unrelated |
|  | Kisely, S., et al. (2020). "Occurrence, prevention, and management of the psychological effects of emerging virus outbreaks on healthcare workers: rapid review and meta-analysis." The BMJ 369: m1642. doi: 10.1136/bmj.m1642 | Unrelated |
|  | Krishnaratne, S., et al. (2020). "Measures implemented in the school setting to contain the COVID‐19 pandemic: a rapid scoping review." Cochrane Database of Systematic Reviews (12). doi: 10.1002/14651858.CD013812 | Unrelated |
|  | Langford, B. J., et al. (2020). "Bacterial co-infection and secondary infection in patients with COVID-19: a living rapid review and meta-analysis." Clinical Microbiology and Infection 26(12): 1622-1629. doi: 10.1016/j.cmi.2020.07.016 | Unrelated |
|  | Levin, A. T., et al. (2020). "Assessing the age specificity of infection fatality rates for COVID-19: systematic review, meta-analysis, and public policy implications." European Journal of Epidemiology 35(12): 1123-1138. doi: 10.1007/s10654-020-00698-1 | Unrelated |
|  | Madewell, Z. J., et al. (2020). "Household Transmission of SARS-CoV-2: A Systematic Review and Meta-analysis." JAMA Network Open: e2031756. doi: 10.1001/jamanetworkopen.2020.31756 | Unrelated |
|  | McNett, M., et al. (2020). "The Global Consortium Study of Neurological Dysfunction in COVID-19 (GCS-NeuroCOVID): Development of Case Report Forms for Global Use." Neurocritical Care 33(3): 793-828. doi: 10.1007/s12028-020-01100-4 | Unrelated |
|  | Melo, A. S., et al. (2020). "FRIENDLY PROTOCOL FOR OOCYTE DONORS: FERTILITY OPTIMIZATION IN TIMES OF SOCIAL ISOLATION DUE TO COVID-19?" Fertility and Sterility 114(3 Supplement): e460-e461. doi: 10.1016/j.fertnstert.2020.08.1327 | Unrelated |
|  | Melo, G. C. d. and K. C. G. M. d. Araujo (2020). "COVID-19 infection in pregnant women, preterm delivery, birth weight, and vertical transmission: a systematic review and meta-analysis." Cadernos de saude publica 36(7): e00087320. doi: 10.1590/0102-311x00087320 | Unrelated |
|  | Meyerowitz-Katz, G. and L. Merone (2020). "A systematic review and meta-analysis of published research data on COVID-19 infection fatality rates." International journal of infectious diseases: IJID: official publication of the International Society for Infectious Diseases 101: 138-148. doi: 10.1016/j.ijid.2020.09.1464 | Unrelated |
|  | Momtazmanesh, S., et al. (2020). "Cardiovascular disease in COVID-19: a systematic review and meta-analysis of 10,898 patients and proposal of a triage risk stratification tool." Egyptian Heart Journal 72(1): 41. https://dx.doi: 10.1186/s43044-020-00075-z | Unrelated |
|  | Piechotta, V., et al. (2020). "Convalescent plasma or hyperimmune immunoglobulin for people with COVID-19: a living systematic review." The Cochrane database of systematic reviews 7: CD013600. doi: 10.1002/14651858.CD013600.pub2 | Unrelated |
|  | Rogers, J. P., et al. (2020). "Psychiatric and neuropsychiatric presentations associated with severe coronavirus infections: a systematic review and meta-analysis with comparison to the COVID-19 pandemic." The lancet. Psychiatry. doi: 10.1016/S2215-0366%2820%2930203-0 | Unrelated |
|  | Rokkas, T. (2020). "Gastrointestinal involvement in covid-19: A systematic review and meta-analysis." Annals of Gastroenterology 33(4): 355-365. doi: 10.20524/aog.2020.0506 | Unrelated |
|  | Roncon, L., et al. (2020). "Diabetic patients with COVID-19 infection are at higher risk of ICU admission and poor short-term outcome." Journal of clinical virology: the official publication of the Pan American Society for Clinical Virology 127: 104354. doi: 10.1016/j.jcv.2020.104354 | Unrelated |
|  | Roshandel, M. R., et al. (2020). "Diagnostic and methodological evaluation of studies on the urinary shedding of SARS-CoV-2, compared to stool and serum: A systematic review and meta-analysis." Cellular and molecular biology (Noisy-le-Grand, France) 66(6): 148-156. | Unrelated |
|  | Sun, Z., et al. (2020). "A systematic review of chest imaging findings in COVID-19." Quantitative Imaging in Medicine and Surgery 10(5): 1058-1079. doi: 10.21037/QIMS-20-564 | Unrelated |
|  | Thomas, B., et al. (2020). "Maternal and perinatal outcomes and pharmacological management of Covid-19 infection in pregnancy: a systematic review protocol." Systematic Reviews 9(1): 161. doi: 10.1186/s13643-020-01418-2 | Unrelated |
|  | Wang, Y., et al. (2020). "Cerebrovascular disease is associated with the risk of mortality in coronavirus disease 2019." Neurological sciences: official journal of the Italian Neurological Society and of the Italian Society of Clinical Neurophysiology 41(8): 2017-2019. doi: 10.1007/s10072-020-04542-y | Unrelated |
|  | Weiss, A., et al. (2020). "Spatial and temporal dynamics of SARS-CoV-2 in COVID-19 patients: A systematic review and meta-analysis." eBioMedicine 58: 102916. doi: 10.1016/j.ebiom.2020.102916 | Unrelated |
|  | Wu, B.-B., et al. (2020). "Association between ABO blood groups and COVID-19 infection, severity and demise: A systematic review and meta-analysis." Infection, genetics and evolution: journal of molecular epidemiology and evolutionary genetics in infectious diseases 84: 104485. doi: 10.1016/j.meegid.2020.104485 | Unrelated |
|  | Yan, H., et al. (2020). "Mental Health of Pregnant and Postpartum Women During the Coronavirus Disease 2019 Pandemic: A Systematic Review and Meta-Analysis." Frontiers in psychology 11: 617001. doi: 10.3389/fpsyg.2020.617001 | Unrelated |
|  | Yanes-Lane, M., et al. (2020). "Proportion of asymptomatic infection among COVID-19 positive persons and their transmission potential: A systematic review and meta-analysis." PLoS ONE 15(11 November): e0241536. doi: 10.1371/journal.pone.0241536 | Unrelated |
|  | Yang, H., et al. (2020). "The chest CT features of coronavirus disease 2019 (COVID-19) in China: a meta-analysis of 19 retrospective studies." Virology journal 17(1): 159. doi: 10.1186/s12985-020-01432-9 | Unrelated |
|  | Yang, Z. and Y. Liu (2020). "Vertical Transmission of Severe Acute Respiratory Syndrome Coronavirus 2: A Systematic Review." American Journal of Perinatology. doi: 10.1055/s-0040-1712161 | Unrelated |
|  | Yap, M., et al. (2020). "Clinical manifestations, prevalence, risk factors, outcomes, transmission, diagnosis and treatment of COVID-19 in pregnancy and postpartum: A living systematic review protocol." BMJ Open 10(12): e041868. doi: 10.1136/bmjopen-2020-041868 | Unrelated |
|  | Yee, J., et al. (2020). "Clinical manifestations and perinatal outcomes of pregnant women with COVID-19: a systematic review and meta-analysis." Scientific reports 10(1): 18126. doi: 10.1038/s41598-020-75096-4 | Unrelated |
|  | Zhou, S., et al. (2020). "Coronavirus-associated kidney outcomes in COVID-19, SARS, and MERS: a meta-analysis and systematic review." Renal Failure 43(1): 1-15. doi: 10.1080/0886022X.2020.1847724 | Unrelated |
|  | Zhu, J., et al. (2020). "Clinical characteristics of 3062 COVID-19 patients: A meta-analysis." Journal of Medical Virology 92(10): 1902-1914. doi: 10.1002/jmv.25884 | Unrelated |
|  | Akem Dimala, C., et al. (2021). "Community and provider acceptability of the COVID-19 vaccine: A systematic review and meta-analysis." Tropical Medicine and International Health 26(SUPPL 1): 246-247. https://dx.doi: 10.1111/tmi.13632 | Unrelated |
|  | Aktaa, S., et al. (2021). "Incidence and mortality due to thromboembolic events during the COVID-19 pandemic: multi-sourced population-based health records cohort study." Thrombosis Research 202: 17-23. doi: 10.1016/j.thromres.2021.03.006 | Unrelated |
|  | Al Kiyumi, M., et al. (2021). "The impact of vitamin d deficiency on the severity of symptoms and mortality rate among adult patients with Covid-19: A systematic review and meta-analysis." Indian Journal of Endocrinology and Metabolism 25(4): 261-282. doi: 10.4103/ijem.ijem_115_21 | Unrelated |
|  | Al-Namaeh, M. (2021). "COVID-19 and conjunctivitis: a meta-analysis." Therapeutic advances in ophthalmology 13: 25158414211003368. doi: 10.1177/25158414211003368 | Unrelated |
|  | Alfano, G., et al. (2021). "The frail world of haemodialysis patients in the COVID-19 pandemic era: a systematic scoping review." Journal of nephrology 34(5): 1387-1403. doi: 10.1007/s40620-021-01136-5 | Unrelated |
|  | Anandraj Vaithy, K., et al. (2021). "Rapid diagnosis of tuberculous lymphadenitis using polymerase chain reaction with IS6110. A 5-year meta-analytic study with special emphasis on predicted impact of COVID-19 associated social stigma and guidance for the implementation of the End TB Strategies." Journal International Medical Sciences Academy 34(2): 92-100. | Unrelated |
|  | Aragoneses, J., et al. (2021). "Oral Manifestations of COVID-19: Updated Systematic Review With Meta-Analysis." Frontiers in Medicine 8: 726753. doi: 10.3389/fmed.2021.726753 | Unrelated |
|  | Arfaras-Melainis, A., et al. (2021). "Effect of hydroxychloroquine on qtc in patients diagnosed with covid-19: A systematic review and meta-analysis." Journal of Cardiovascular Development and Disease 8(5): 55. doi: 10.3390/jcdd8050055 | Unrelated |
|  | Asadi-Pooya, A. A., et al. (2021). "COVID-19, de novo seizures, and epilepsy: a systematic review." Neurological Sciences 42(2): 415-431. doi: 10.1007/s10072-020-04932-2 | Unrelated |
|  | Bakhit, M., et al. (2021). "Antibiotic prescribing for acute infections in synchronous telehealth consultations: a systematic review and meta-analysis." BJGP Open 5(6). doi: 10.3399/BJGPO.2021.0106 | Unrelated |
|  | Bakolis, I., et al. (2021). "Changes in daily mental health service use and mortality at the commencement and lifting of COVID-19 'lockdown' policy in 10 UK sites: A regression discontinuity in time design." BMJ Open 11(5): e049721. doi: 10.1136/bmjopen-2021-049721 | Unrelated |
|  | Balderas-Delgado, C., et al. (2021). "Pregnancy and COVID-19. Considerations in the practice of Hematology." Gaceta Medica de Mexico 157(Supplement 3): S112-S119. doi: 10.24875/GMM.M21000486 | Unrelated |
|  | Baldini, T., et al. (2021). "Cerebral venous thrombosis and severe acute respiratory syndrome coronavirus-2 infection: A systematic review and meta-analysis." European Journal of Neurology 28(10): 3478-3490. doi: 10.1111/ene.14727 | Unrelated |
|  | Bann, D., et al. (2021). "Changes in the behavioural determinants of health during the COVID-19 pandemic: Gender, socioeconomic and ethnic inequalities in five British cohort studies." Journal of Epidemiology and Community Health 75(12): 1136-1142. doi: 10.1136/jech-2020-215664 | Unrelated |
|  | Belletti, A., et al. (2021). "Barotrauma in Coronavirus Disease 2019 Patients Undergoing Invasive Mechanical Ventilation: A Systematic Literature Review." Critical Care Medicine. doi: 10.1097/CCM.0000000000005283 | Unrelated |
|  | Bellos, I., et al. (2021). "Maternal and perinatal outcomes in pregnant women infected by SARS-CoV-2: A meta-analysis." European Journal of Obstetrics and Gynecology and Reproductive Biology 256: 194-204. doi: 10.1016/j.ejogrb.2020.11.038 | Unrelated |
|  | Belsky, J. A., et al. (2021). "COVID-19 in immunocompromised patients: A systematic review of cancer, hematopoietic cell and solid organ transplant patients." The Journal of infection 82(3): 329-338. doi: 10.1016/j.jinf.2021.01.022 | Unrelated |
|  | Bergeri, I., et al. (2021). "Global SARS-CoV-2 seroprevalence: a systematic review and meta-analysis of standardized population-based studies from Jan 2020-May 2022." medRxiv. doi: 10.1101/2021.12.14.21267791 | Unrelated |
|  | Bobrovitz, N., et al. (2021). "Global seroprevalence of SARS-CoV-2 antibodies: A systematic review and meta-analysis." PLoS ONE 16(6): e0252617. doi: 10.1371/journal.pone.0252617 | Unrelated |
|  | Bolarinwa, O. A., et al. (2021). "Mapping Evidence of Impacts of COVID-19 Outbreak on Sexual and Reproductive Health: A Scoping Review." Healthcare (Basel, Switzerland) 9(4). doi: 10.3390/healthcare9040436 | Unrelated |
|  | Borges, L. P., et al. (2021). "Rapid diagnosis of COVID-19 in the first year of the pandemic: A systematic review." International Immunopharmacology 101: 108144. doi: 10.1016/j.intimp.2021.108144 | Unrelated |
|  | Byambasuren, O., et al. (2021). "Estimating the extent of asymptomatic covid-19 and its potential for community transmission: Systematic review and meta-analysis." Canadian Journal of Infection Control 36(1): 30-38. | Unrelated |
|  | Cai, J., et al. (2021). "Cesarean Section or Vaginal Delivery to Prevent Possible Vertical Transmission From a Pregnant Mother Confirmed With COVID-19 to a Neonate: A Systematic Review." Frontiers in Medicine 8: 634949. doi: 10.3389/fmed.2021.634949 | Unrelated |
|  | Cai, X., et al. (2021). "Risk Factors for Acute Kidney Injury in Adult Patients With COVID-19: A Systematic Review and Meta-Analysis." Frontiers in Medicine 8: 719472. doi: 10.3389/fmed.2021.719472 | Unrelated |
|  | Caramaschi, S., et al. (2021). "Histopathological findings and clinicopathologic correlation in COVID-19: a systematic review." Modern Pathology 34(9): 1614-1633. doi: 10.1038/s41379-021-00814-w | Unrelated |
|  | Chai, S., et al. (2021). "Effect of age of COVID-19 inpatient on the severity of the disease: A meta-analysis." International Journal of Clinical Practice 75(10): e14640. doi: 10.1111/ijcp.14640 | Unrelated |
|  | Chen, C., et al. (2021). "The epidemiological and radiographical characteristics of asymptomatic infections with the novel coronavirus (COVID-19): A systematic review and meta-analysis." International journal of infectious diseases: IJID : official publication of the International Society for Infectious Diseases. doi: 10.1016/j.ijid.2021.01.017 | Unrelated |
|  | Chen, M., et al. (2021). "Safety of SARS-CoV-2 vaccines: a systematic review and meta-analysis of randomized controlled trials." Infectious Diseases of Poverty 10(1): 94. doi: 10.1186/s40249-021-00878-5 | Unrelated |
|  | Chen, X., et al. (2021). "Ratio of asymptomatic COVID-19 cases among ascertained SARS-CoV-2 infections in different regions and population groups in 2020: a systematic review and meta-analysis including 130 123 infections from 241 studies." BMJ Open 11(12): e049752. doi: 10.1136/bmjopen-2021-049752 | Unrelated |
|  | Chen, Y., et al. (2021). "The Association Between COVID-19 and Thyroxine Levels: A Meta-Analysis." Frontiers in Endocrinology 12: 779692. doi: 10.3389/fendo.2021.779692 | Unrelated |
|  | Cheng, C., et al. (2021). "The incubation period of COVID-19: a global meta-analysis of 53 studies and a Chinese observation study of 11 545 patients." Infectious Diseases of Poverty 10(1): 119. doi: 10.1186/s40249-021-00901-9 | Unrelated |
|  | Chmielewska, B., et al. (2021). "Effects of the COVID-19 pandemic on maternal and perinatal outcomes: a systematic review and meta-analysis." The Lancet. Global health. doi: 10.1016/S2214-109X%2821%2900079-6 | Unrelated |
|  | Cho, J., et al. (2021). "Extrapulmonary manifestations and complications of severe acute respiratory syndrome coronavirus 2 infection: a systematic review." Singapore medical journal. doi: 10.11622/smedj.2021100 | Unrelated |
|  | Chua, P. E. Y., et al. (2021). "Epidemiological and clinical characteristics of non-severe and severe pediatric and adult COVID-19 patients across different geographical regions in the early phase of pandemic: a systematic review and meta-analysis of observational studies." Journal of investigative medicine: the official publication of the American Federation for Clinical Research. doi: 10.1136/jim-2021-001858 | Unrelated |
|  | Chun, H. S., et al. (2021). "A Meta-Analysis of Treatment Effects on Viral Pneumonia Using TCM Injections Specified in the Clinical Guideline for COVID-19 in China." Journal of Pharmacopuncture 24(3): 107-121. doi: 10.3831/KPI.2021.24.3.107 | Unrelated |
|  | Cohen, C., et al. (2021). "SARS-CoV-2 incidence, transmission and reinfection in a rural and an urban setting: results of the PHIRST-C cohort study, South Africa, 2020-2021." medRxiv: the preprint server for health sciences. doi: 10.1101/2021.07.20.21260855 | Unrelated |
|  | Dao, T. L., et al. (2021). "Co-infection of SARS-CoV-2 and influenza viruses: A systematic review and meta-analysis." Journal of clinical virology plus 1(3): 100036. doi: 10.1016/j.jcvp.2021.100036 | Unrelated |
|  | DeArmond, M., et al. (2021). "Diagnostic accuracy of rapid antigen tests for COVID-19 compared to the viral genetic test in adults: a systematic review protocol." JBI evidence synthesis 19(5): 1148-1156. doi: 10.11124/JBIES-20-00561 | Unrelated |
|  | Dhillon, R. A., et al. (2021). "The mystery of COVID-19 reinfections: A global systematic review and meta-analysis." Annals of Medicine and Surgery 72: 103130. doi: 10.1016/j.amsu.2021.103130 | Unrelated |
|  | Du, Y., et al. (2021). "Association of body mass index (BMI) with critical COVID-19 and in-hospital mortality: A dose-response meta-analysis." Metabolism: Clinical and Experimental 117: 154373. doi: 10.1016/j.metabol.2020.154373 | Unrelated |
|  | Dubey, P., et al. (2021). "Current trends and geographical differences in therapeutic profile and outcomes of COVID-19 among pregnant women - a systematic review and meta-analysis." BMC Pregnancy and Childbirth 21(1): 247. doi: 10.1186/s12884-021-03685-w | Unrelated |
|  | Eberle, C. and S. Stichling (2021). "Telemedical Approaches to Managing Gestational Diabetes Mellitus During COVID-19: Systematic Review." JMIR pediatrics and parenting 4(3): e28630. doi: 10.2196/28630 | Unrelated |
|  | Elsawah, H. K., et al. (2021). "Efficacy and safety of remdesivir in hospitalized Covid-19 patients: Systematic review and meta-analysis including network meta-analysis." Reviews in Medical Virology 31(4): e2187. doi: 10.1002/rmv.2187 | Unrelated |
|  | Emile, S. H., et al. (2021). "Rate of Application and Outcome of Non-operative Management of Acute Appendicitis in the Setting of COVID-19: Systematic Review and Meta-analysis." Journal of gastrointestinal surgery: official journal of the Society for Surgery of the Alimentary Tract 25(7): 1905-1915. doi: 10.1007/s11605-021-04988-1 | Unrelated |
|  | Falsaperla, R., et al. (2021). "Neonates Born to COVID-19 Mother and Risk in Management within 4 Weeks of Life: A Single-Center Experience, Systematic Review, and Meta-Analysis." American Journal of Perinatology. doi: 10.1055/s-0041-1729557 | Unrelated |
|  | Funakoshi, K., et al. (2021). "Longer Prehospitalization and Preintubation Periods in Intubated Non-survivors and ECMO Patients With COVID-19: A Systematic Review and Meta-Analysis." Frontiers in Medicine 8: 727101. doi: 10.3389/fmed.2021.727101 | Unrelated |
|  | Gajbhiye, R. K., et al. (2021). "Differential impact of COVID-19 in pregnant women from high-income countries and low- to middle-income countries: A systematic review and meta-analysis." International journal of gynaecology and obstetrics: the official organ of the International Federation of Gynaecology and Obstetrics. doi: 10.1002/ijgo.13793 | Unrelated |
|  | Garg, M., et al. (2021). "Diagnostic accuracy of CT and radiographic findings for novel coronavirus 2019 pneumonia: Systematic review and meta-analysis." Clinical imaging 72: 75-82. doi: 10.1016/j.clinimag.2020.11.021 | Unrelated |
|  | Gastine, S., et al. (2021). "Systematic Review and Patient-Level Meta-Analysis of SARS-CoV-2 Viral Dynamics to Model Response to Antiviral Therapies." Clinical Pharmacology and Therapeutics 110(2): 321-333. doi: 10.1002/cpt.2223 | Unrelated |
|  | Gholami, M., et al. (2021). "COVID-19 and healthcare workers: A systematic review and meta-analysis." International journal of infectious diseases: IJID: official publication of the International Society for Infectious Diseases 104: 335-346. doi: 10.1016/j.ijid.2021.01.013 | Unrelated |
|  | Giesbers, S., et al. (2021). "Treatment of COVID-19 in pregnant women: A systematic review and meta-analysis." European Journal of Obstetrics and Gynecology and Reproductive Biology 267: 120-128. doi: 10.1016/j.ejogrb.2021.10.007 | Unrelated |
|  | Goh, X. L., et al. (2021). "Incidence of SARS-CoV-2 vertical transmission: A meta-analysis." Archives of Disease in Childhood: Fetal and Neonatal Edition 106(1): 112-113. doi: 10.1136/archdischild-2020-319791 | Unrelated |
|  | Granger, C., et al. (2021). "Clinical manifestations and outcomes of coronavirus disease-19 in heart transplant recipients: a multicentre case series with a systematic review and meta-analysis." Transplant international: official journal of the European Society for Organ Transplantation 34(4): 721-731. doi: 10.1111/tri.13837 | Unrelated |
|  | Green, M. S., D. Nitzan, N. Schwartz, Y. Niv & V. Peer (2021) Sex differences in the case-fatality rates for COVID-19-A comparison of the age-related differences and consistency over seven countries. PLoS ONE, 16, e0250523. doi: 10.1371/journal.pone.0250523 | Unrelated |
|  | Groff, D., A. Sun, A. E. Ssentongo, D. M. Ba, N. Parsons, G. R. Poudel, A. Lekoubou, J. S. Oh, J. E. Ericson, P. Ssentongo & V. M. Chinchilli (2021) Short-term and Long-term Rates of Postacute Sequelae of SARS-CoV-2 Infection: A Systematic Review. JAMA Network Open, 4, e2128568. doi: 10.1001/jamanetworkopen.2021.28568 | Unrelated |
|  | Haiduc, A. A., M. Ogunjimi, R. Shammus, S. Mahmood, R. Kutty, A. Lotto, R. Guerrero, A. Harky & R. Dhannapuneni (2021) COVID-19 and congenital heart disease: an insight of pathophysiology and associated risks. Cardiology in the young, 31, 233-240. doi: 10.1017/S1047951120003741 | Unrelated |
|  | Hayashi, Y., K. Wagatsuma, M. Nojima, T. Yamakawa, T. Ichimiya, Y. Yokoyama, T. Kazama, D. Hirayama & H. Nakase (2021) The characteristics of gastrointestinal symptoms in patients with severe COVID-19: a systematic review and meta-analysis. Journal of Gastroenterology, 56, 409-420. doi: 10.1007/s00535-021-01778-z | Unrelated |
|  | He, J., Y. Guo, R. Mao & J. Zhang (2021) Proportion of asymptomatic coronavirus disease 2019: A systematic review and meta-analysis. Journal of Medical Virology, 93, 820-830. doi: 10.1002/jmv.26326 | Unrelated |
|  | Hernandez-Diaz, S., B. T. Bateman, L. Straub, Y. Zhu, H. Mogun, M. Fischer & K. F. Huybrechts (2021) Safety of Tenofovir Disoproxil Fumarate for Pregnant Women Facing the Coronavirus Disease 2019 Pandemic. American Journal of Epidemiology, 190, 2339-2349. doi: 10.1093/aje/kwab109 | Unrelated |
|  | Huntley, B. J. F., I. A. Mulder, D. Di Mascio, W. S. Vintzileos, A. M. Vintzileos, V. Berghella & S. P. Chauhan (2021) Adverse Pregnancy Outcomes Among Individuals With and Without Severe Acute Respiratory Syndrome Coronavirus 2 (SARS-CoV-2) A Systematic Review and Meta-analysis. Obstetrics and Gynecology, 137, 585-596. doi: 10.1097/AOG.0000000000004320 | Unrelated |
|  | Islam, M. A., S. Kundu, S. S. Alam, T. Hossan, M. A. Kamal & R. Hassan (2021) Prevalence and characteristics of fever in adult and paediatric patients with coronavirus disease 2019 (COVID-19): A systematic review and meta-analysis of 17515 patients. PLoS ONE, 16, e0249788. doi: 10.1371/journal.pone.0249788 | Unrelated |
|  | Jafari, M., A. Pormohammad, S. A. Sheikh Neshin, S. Ghorbani, D. Bose, S. Alimohammadi, S. Basirjafari, M. Mohammadi, C. Rasmussen-Ivey, M. H. Razizadeh, M. Nouri-Vaskeh & M. Zarei (2021) Clinical characteristics and outcomes of pregnant women with COVID-19 and comparison with control patients: A systematic review and meta-analysis. Reviews in medical virology, e2208. doi: 10.1002/rmv.2208 | Unrelated |
|  | Jolliffe, D. A., C. A. Camargo, J. D. Sluyter, M. Aglipay, J. F. Aloia, D. Ganmaa, P. Bergman, H. A. Bischoff-Ferrari, A. Borzutzky, C. T. Damsgaard, G. Dubnov-Raz, S. Esposito, C. Gilham, A. A. Ginde, I. Golan-Tripto, E. C. Goodall, C. C. Grant, C. J. Griffiths, A. M. Hibbs, W. Janssens, A. V. Khadilkar, I. Laaksi, M. T. Lee, M. Loeb, J. L. Maguire, P. Majak, D. T. Mauger, S. Manaseki-Holland, D. R. Murdoch, A. Nakashima, R. E. Neale, H. Pham, C. Rake, J. R. Rees, J. Rosendahl, R. Scragg, D. Shah, Y. Shimizu, S. Simpson-Yap, G. Trilok-Kumar, M. Urashima & A. R. Martineau (2021) Vitamin D supplementation to prevent acute respiratory infections: a systematic review and meta-analysis of aggregate data from randomised controlled trials. The lancet. Diabetes & endocrinology. doi: 10.1016/S2213-8587%2821%2900051-6 | Unrelated |
|  | Kaggwa, M. M., J. Kajjimu, J. Sserunkuma, S. M. Najjuka, L. M. Atim, R. Olum, A. Tagg & F. Bongomin (2021) Prevalence of burnout among university students in low- and middle-income countries: A systematic review and meta-analysis. PloS one, 16, e0256402. doi: 10.1371/journal.pone.0256402 | Unrelated |
|  | Karki, S. J., Joachim, A., Heinsohn, T., and Lange, B. (2021). Risk of infection and contribution to transmission of SARS-CoV-2 in school staff: A systematic review. BMJ Open 11, e052690. doi: 10.1136/bmjopen-2021-052690 | Unrelated |
|  | Kaye, A. D., Cornett, E. M., Brondeel, K. C., Lerner, Z. I., Knight, H. E., Erwin, A., Charipova, K., Gress, K. L., Urits, I., Urman, R. D., Fox, C. J., and Kevil, C. G. (2021). Biology of COVID-19 and related viruses: Epidemiology, signs, symptoms, diagnosis, and treatment. Best Practice and Research: Clinical Anaesthesiology 35, 269-292. doi: 10.1016/j.bpa.2020.12.003 | Unrelated |
|  | Khan, A. (2021). Pre-Conception and Pregnancy Parental Exposure to Pesticides and Pediatric Neuroblastoma. A Meta-Analysis of Nine Studies. Pediatric Blood and Cancer 68. doi: 10.1002/pbc.29349 | Unrelated |
|  | Khan, D. S. A., Hamid, L.-R., Ali, A., Salam, R. A., Zuberi, N., Lassi, Z. S., and Das, J. K. (2021). Differences in pregnancy and perinatal outcomes among symptomatic versus asymptomatic COVID-19-infected pregnant women: a systematic review and meta-analysis. BMC pregnancy and childbirth 21, 801. doi: 10.1186/s12884-021-04250-1 | Unrelated |
|  | Khera, D., Chugh, A., Khasbage, S., and Singh, S. (2021). Does Bacille Calmette-Guerin Vaccination Provides Protection against COVID-19: A Systematic Review and Meta-analysis. Indian journal of community medicine: official publication of Indian Association of Preventive & Social Medicine 46, 592-599. doi: 10.4103/ijcm.IJCM_952_20 | Unrelated |
|  | Kohler, F., Muller, S., Hendricks, A., Kastner, C., Reese, L., Boerner, K., Flemming, S., Lock, J. F., Germer, C.-T., and Wiegering, A. (2021). Changes in appendicitis treatment during the COVID-19 pandemic - A systematic review and meta-analysis. International journal of surgery (London, England) 95, 106148. doi: 10.1016/j.ijsu.2021.106148 | Unrelated |
|  | Kotlyar, A. M., Grechukhina, O., Chen, A., Popkhadze, S., Grimshaw, A., Tal, O., Taylor, H. S., and Tal, R. (2021). Vertical transmission of coronavirus disease 2019: a systematic review and meta-analysis. American journal of obstetrics and gynecology 224, 35-53. e3. doi: 10.1016/j.ajog.2020.07.049 | Unrelated |
|  | Kow, C. S., and Hasan, S. S. (2021). Real-world effectiveness of BNT162b2 mRNA vaccine: a meta-analysis of large observational studies. Inflammopharmacology 29, 1075-1090. doi: 10.1007/s10787-021-00839-2 | Unrelated |
|  | Kow, C. S., and Hasan, S. S. (2021). A meta-analysis on the preadmission use of DPP-4 inhibitors and risk of a fatal or severe course of illness in patients with COVID-19. Therapies 76, 361-364. doi: 10.1016/j.therap.2020.12.015 | Unrelated |
|  | Kunzler, A. M., Rothke, N., Gunthner, L., Stoffers-Winterling, J., Tuscher, O., Coenen, M., Rehfuess, E., Schwarzer, G., Binder, H., Schmucker, C., Meerpohl, J. J., and Lieb, K. (2021). Mental burden and its risk and protective factors during the early phase of the SARS-CoV-2 pandemic: systematic review and meta-analyses. Globalization and health 17, 34. doi: 10.1186/s12992-021-00670-y | Unrelated |
|  | La Verde, M., Riemma, G., Torella, M., Cianci, S., Savoia, F., Licciardi, F., Scida, S., Morlando, M., Colacurci, N., and De Franciscis, P. (2021). Maternal death related to COVID-19: A systematic review and meta-analysis focused on maternal co-morbidities and clinical characteristics. International journal of gynaecology and obstetrics: the official organ of the International Federation of Gynaecology and Obstetrics 154, 212-219. doi: 10.1002/ijgo.13726 | Unrelated |
|  | Langford, B. J., So, M., Raybardhan, S., Leung, V., Soucy, J.-P. R., Westwood, D., Daneman, N., and MacFadden, D. R. (2021). Antibiotic prescribing in patients with COVID-19: rapid review and meta-analysis. Clinical microbiology and infection: the official publication of the European Society of Clinical Microbiology and Infectious Diseases 27, 520-531. doi: 10.1016/j.cmi.2020.12.018 | Unrelated |
|  | Lassi, Z. S., Ana, A., Das, J. K., Salam, R. A., Padhani, Z. A., Irfan, O., and Bhutta, Z. A. (2021). A systematic review and meta-analysis of data on pregnant women with confirmed COVID-19: Clinical presentation, and pregnancy and perinatal outcomes based on COVID-19 severity. Journal of global health 11, 05018. doi: 10.7189/jogh.11.05018 | Unrelated |
|  | Lawson, H., Sheikh, J., Yap, M., Chatterjee, S., Kew, T., Debenham, L., Llavall, A. N., Dixit, A., Balaji, R., Zhou, D., Ansari, K., Allotey, J., Stallings, E., Bonet, M., Lee, S. I., Qiu, X., Yuan, M., Coomar, D., Van Wely, M., Van Leeuwen, E., Kostova, E., Kunst, H., Khalil, A., Tiberi, S., Brizuela, V., Broutet, N., Kara, E., Kim, C. R., Thorson, A., Oladapo, O., Mofenson, L., Zamora, J., and Thangaratinam, S. (2021). Maternal and perinatal outcomes in pregnant and recently pregnant women with coronavirus disease 2019: Living systematic review and meta-analysis. BJOG: An International Journal of Obstetrics and Gynaecology 128, 81-82. doi: 10.1111/1471-0528.16710 | Unrelated |
|  | Lee, H.-Y., Ahn, J., Park, J., Kang, C. K., Won, S.-H., Kim, D. W., Park, J.-H., Chung, K.-H., Joh, J.-S., Bang, J. H., Kang, C. H., Oh, M.-D., and Pyun, W. B. (2021). Different therapeutic associations of renin-angiotensin system inhibitors with coronavirus disease 2019 compared with usual pneumonia. The Korean journal of internal medicine 36, 617-628. doi: 10.3904/kjim.2020.656 | Unrelated |
|  | Lee, R. A., Herigon, J. C., Benedetti, A., Pollock, N. R., and Denkinger, C. M. (2021). Performance of Saliva, Oropharyngeal Swabs, and Nasal Swabs for SARS-CoV-2 Molecular Detection: A Systematic Review and Meta-analysis. Journal of clinical microbiology. doi: 10.1128/JCM.02881-20 | Unrelated |
|  | Lewis, L., Losty, P., and Sinha, I. (2021). Bronchiolitis prevalence rates in Congenital Diaphragmatic Hernia: a systematic review and meta-analysis. European Respiratory Journal 58. doi: 10.1183/13993003.congress-2021.PA1959 | Unrelated |
|  | Li, F., Lu, H., Zhang, Q., Li, X., Wang, T., Liu, Q., Yang, Q., and Qiang, L. (2021). Impact of COVID-19 on female fertility: A systematic review and meta-Analysis protocol. BMJ Open 11, e045524. doi: 10.1136/bmjopen-2020-045524 | Unrelated |
|  | Li, J., Liao, X., Zhou, Y., Wang, L., Yang, H., Zhang, W., Zhang, Z., and Kang, Y. (2021). Association between glucocorticoids treatment and viral clearance delay in patients with COVID-19: a systematic review and meta-analysis. BMC Infectious Diseases 21, 1063. doi: 10.1186/s12879-021-06548-z | Unrelated |
|  | Li, X., Ostropolets, A., Makadia, R., Shaoibi, A., Rao, G., Sena, A. G., Martinez-Hernandez, E., Delmestri, A., Verhamme, K., Rijnbeek, P. R., Duarte-Salles, T., Suchard, M., Ryan, P., Hripcsak, G., and Prieto-Alhambra, D. (2021). Characterizing the incidence of adverse events of special interest for COVID-19 vaccines across eight countries: a multinational network cohort study. medRxiv: the preprint server for health sciences. doi: 10.1101/2021.03.25.21254315 | Unrelated |
|  | Liyanage, P., Rocklov, J., and Tissera, H. A. (2021). The impact of covid-19 lockdown on dengue transmission in sri lanka; a natural experiment for understanding the influence of human mobility. PLoS Neglected Tropical Diseases 15, e0009420. doi: 10.1371/journal.pntd.0009420 | Unrelated |
|  | Ma, Q., Liu, J., Liu, Q., Kang, L., Liu, R., Jing, W., Wu, Y., and Liu, M. (2021). Global Percentage of Asymptomatic SARS-CoV-2 Infections Among the Tested Population and Individuals With Confirmed COVID-19 Diagnosis: A Systematic Review and Meta-analysis. JAMA network open 4, e2137257. doi: 10.1001/jamanetworkopen.2021.37257 | Unrelated |
|  | Macedo, A., Goncalves, N., and Febra, C. (2021). COVID-19 fatality rates in hospitalized patients: systematic review and meta-analysis. Annals of Epidemiology 57, 14-21. doi: 10.1016/j.annepidem.2021.02.012 | Unrelated |
|  | Madewell, Z. J., Yang, Y., Longini, I. M., Jr., Halloran, M. E., and Dean, N. E. (2021). Factors Associated With Household Transmission of SARS-CoV-2: An Updated Systematic Review and Meta-analysis. JAMA network open 4, e2122240. doi: 10.1001/jamanetworkopen.2021.22240 | Unrelated |
|  | Mai, V., Tan, B. K., Mainbourg, S., Potus, F., Cucherat, M., Lega, J.-C., and Provencher, S. (2021). Venous thromboembolism in COVID-19 compared to non-COVID-19 cohorts: A systematic review with meta-analysis. Vascular pharmacology 139, 106882. doi: 10.1016/j.vph.2021.106882 | Unrelated |
|  | Mair, M., Singhavi, H., Pai, A., Singhavi, J., Gandhi, P., Conboy, P., Baker, A., and Das, S. (2021). A Meta-Analysis of 67 Studies with Presenting Symptoms and Laboratory Tests of COVID-19 Patients. Laryngoscope 131, 1254-1265. doi: 10.1002/lary.29207 | Unrelated |
|  | Manabe, T., Kambayashi, D., Akatsu, H., and Kudo, K. (2021). Favipiravir for the treatment of patients with COVID-19: a systematic review and meta-analysis. BMC infectious diseases 21, 489. doi: 10.1186/s12879-021-06164-x | Unrelated |
|  | Manoharan, L., Cattrall, J. W. S., Harris, C., Newell, K., Thomson, B., Pritchard, M. G., Bannister, P. G., Sigfrid, L., Solomon, T., Horby, P. W., Carson, G., and Olliaro, P. (2021). Evaluating clinical characteristics studies produced early in the Covid-19 pandemic: A systematic review. PloS one 16, e0251250. doi: 10.1371/journal.pone.0251250 | Unrelated |
|  | Matar, R., Alrahmani, L., Monzer, N., Debiane, L. G., Berbari, E., Fares, J., Fitzpatrick, F., and Murad, M. H. (2021). Clinical Presentation and Outcomes of Pregnant Women with Coronavirus Disease 2019: A Systematic Review and Meta-analysis. Clinical Infectious Diseases 72, 521-533. doi: 10.1093/cid/ciaa828 | Unrelated |
|  | Mazziotti, R., and Rutigliano, G. (2021). Tele-mental health for reaching out to patients in a time of pandemic: Provider survey and meta-analysis of patient satisfaction. JMIR Mental Health 8, e26187. doi: 10.2196/26187 | Unrelated |
|  | McLean, S. A., Booth, A. T., Schnabel, A., Wright, B. J., Painter, F. L., and McIntosh, J. E. (2021). Exploring the Efficacy of Telehealth for Family Therapy Through Systematic, Meta-analytic, and Qualitative Evidence. Clinical child and family psychology review 24, 244-266. doi: 10.1007/s10567-020-00340-2 | Unrelated |
|  | Michelen, M., Manoharan, L., Elkheir, N., Cheng, V., Dagens, A., Hastie, C., O'Hara, M., Suett, J., Dahmash, D., Bugaeva, P., Rigby, I., Munblit, D., Harriss, E., Burls, A., Foote, C., Scott, J., Carson, G., Olliaro, P., Sigfrid, L., and Stavropoulou, C. (2021). Characterising long COVID: A living systematic review. BMJ Global Health 6, e005427. doi: 10.1136/bmjgh-2021-005427 | Unrelated |
|  | Million, M., Lagier, J.-C., Tissot-Dupont, H., Ravaux, I., Dhiver, C., Tomei, C., Cassir, N., Delorme, L., Cortaredona, S., Amrane, S., Aubry, C., Bendamardji, K., Berenger, C., Doudier, B., Edouard, S., Hocquart, M., Mailhe, M., Porcheto, C., Seng, P., Triquet, C., Gentile, S., Jouve, E., Giraud-Gatineau, A., Chaudet, H., Camoin-Jau, L., Colson, P., Gautret, P., Fournier, P.-E., Maille, B., Deharo, J.-C., Habert, P., Gaubert, J.-Y., Jacquier, A., Honore, S., Guillon-Lorvellec, K., Obadia, Y., Parola, P., Brouqui, P., and Raoult, D. (2021). Early combination therapy with hydroxychloroquine and azithromycin reduces mortality in 10,429 COVID-19 outpatients. Reviews in cardiovascular medicine 22, 1063-1072. doi: 10.31083/j.rcm2203116 | Unrelated |
|  | Miniati, M., Marzetti, F., Palagini, L., Marazziti, D., Orru, G., Conversano, C., and Gemignani, A. (2021). Eating Disorders Spectrum During the COVID Pandemic: A Systematic Review. Frontiers in psychology 12, 663376. doi: 10.3389/fpsyg.2021.663376 | Unrelated |
|  | Mousa, M., Vurivi, H., Kannout, H., Uddin, M., Alkaabi, N., Mahboub, B., Tay, G. K., and Alsafar, H. S. (2021). Genome-wide association study of hospitalized COVID-19 patients in the United Arab Emirates. eBioMedicine 74, 103695. doi: 10.1016/j.ebiom.2021.103695 | Unrelated |
|  | Musa, S. S., Bello, U. M., Zhao, S., Abdullahi, Z. U., Lawan, M. A., and He, D. (2021). Vertical Transmission of SARS-CoV-2: A Systematic Review of Systematic Reviews. Viruses 13. doi: 10.3390/v13091877 | Unrelated |
|  | Mutiawati, E., Syahrul, S., Fahriani, M., Fajar, J. K., Mamada, S. S., Maliga, H. A., Samsu, N., Ilmawan, M., Purnamasari, Y., Asmiragani, A. A., Ichsan, I., Emran, T. B., Rabaan, A. A., Masyeni, S., Nainu, F., and Harapan, H. (2021). Global prevalence and pathogenesis of headache in COVID-19: A systematic review and meta-analysis. F1000Research 9, 1-21. doi: 10.12688/F1000RESEARCH.27334.1 | Unrelated |
|  | Nassar, M., Nso, N., Alfishawy, M., Novikov, A., Yaghi, S., Medina, L., Toz, B., Lakhdar, S., Idrees, Z., Kim, Y., Gurung, D. O., Siddiqui, R. S., Zheng, D., Agladze, M., Sumbly, V., Sandhu, J., Castillo, F. C., Chowdhury, N., Kondaveeti, R., Bhuiyan, S., Perez, L. G., Ranat, R., Gonzalez, C., Bhangoo, H., Williams, J., Osman, A. E., Kong, J., Ariyaratnam, J., Mohamed, M., Omran, I., Lopez, M., Nyabera, A., Landry, I., Iqbal, S., Gondal, A. Z., Hassan, S., Daoud, A., Baraka, B., Trandafirescu, T., and Rizzo, V. (2021). Current systematic reviews and meta-analyses of COVID-19. World journal of virology 10, 182-208. doi: 10.5501/wjv.v10.i4.182 | Unrelated |
|  | Novoa, R. H., Quintana, W., Llancari, P., Urbina-Quispe, K., Guevara-Rios, E., and Ventura, W. (2021). Maternal clinical characteristics and perinatal outcomes among pregnant women with coronavirus disease 2019. A systematic review. Travel medicine and infectious disease 39, 101919. doi: 10.1016/j.tmaid.2020.101919 | Unrelated |
|  | Ozlusen, B., Kozan, S., Akcan, R. E., Kalender, M., Yaprak, D., Peltek, I. B., Keske, S., Gonen, M., and Ergonul, O. (2021). Effectiveness of favipiravir in COVID-19: a live systematic review. European Journal of Clinical Microbiology and Infectious Diseases 40, 2575-2583. doi: 10.1007/s10096-021-04307-1 | Unrelated |
|  | Pabalan, N., Tharabenjasin, P., Suntornsaratoon, P., Jarjanazi, H., and Muanprasat, C. (2021). Ethnic and age-specific acute lung injury/acute respiratory distress syndrome risk associated with angiotensin-converting enzyme insertion/deletion polymorphisms, implications for COVID-19: A meta-analysis. Infection, Genetics and Evolution 88, 104682. doi: 10.1016/j.meegid.2020.104682 | Unrelated |
|  | Pashazadeh Kan, F., Raoofi, S., Rafiei, S., Khani, S., Hosseinifard, H., Tajik, F., Raoofi, N., Ahmadi, S., Aghalou, S., Torabi, F., Dehnad, A., Rezaei, S., Hosseinipalangi, Z., and Ghashghaee, A. (2021). A systematic review of the prevalence of anxiety among the general population during the COVID-19 pandemic. Journal of Affective Disorders 293, 391-398. doi: 10.1016/j.jad.2021.06.073 | Unrelated |
|  | Patanavanich, R., and Glantz, S. A. (2021). Smoking is associated with worse outcomes of COVID-19 particularly among younger adults: a systematic review and meta-analysis. BMC public health 21, 1554. doi: 10.1186/s12889-021-11579-x | Unrelated |
|  | Patrick, M. T., Zhang, H., Wasikowski, R., Prens, E. P., Weidinger, S., Gudjonsson, J. E., Elder, J. T., He, K., and Tsoi, L. C. (2021). Associations between COVID-19 and skin conditions identified through epidemiology and genomic studies. The Journal of allergy and clinical immunology 147, 857-869.e7. doi: 10.1016/j.jaci.2021.01.006 | Unrelated |
|  | Pelizzo, G., Silvestro, S., Avanzini, M. A., Zuccotti, G., Mazzon, E., and Calcaterra, V. (2021). Mesenchymal stromal cells for the treatment of interstitial lung disease in children: A look from pediatric and pediatric surgeon viewpoints. Cells 10, 3270. doi: 10.3390/cells10123270 | Unrelated |
|  | Pfeiffer, A. F., James, R., Neuhoff, B. K., Pfeiffer, W. B., Lowery, D. R., and Rizvi, S. A. A. (2021). The deleterious effects of COVID-19 in the peripartum period: A case report. Pediatric Reports 13, 334-339. doi: 10.3390/pediatric13020041 | Unrelated |
|  | Piechotta, V., Iannizzi, C., Chai, K. L., Valk, S. J., Kimber, C., Dorando, E., Monsef, I., Wood, E. M., Lamikanra, A. A., Roberts, D. J., McQuilten, Z., So-Osman, C., Estcourt, L. J., and Skoetz, N. (2021). Convalescent plasma or hyperimmune immunoglobulin for people with COVID-19: a living systematic review. The Cochrane database of systematic reviews 5, CD013600. doi: 10.1002/14651858.CD013600.pub4 | Unrelated |
|  | Rabbani, G., Shariful Islam, S. M., Rahman, M. A., Amin, N., Marzan, B., Robin, R. C., and Alif, S. M. (2021). Pre-existing COPD is associated with an increased risk of mortality and severity in COVID-19: a rapid systematic review and meta-analysis. Expert review of respiratory medicine 15, 705-716. doi: 10.1080/17476348.2021.1866547 | Unrelated |
|  | Raina, R., Chakraborty, R., Mawby, I., Agarwal, N., Sethi, S., and Forbes, M. (2021). Critical analysis of acute kidney injury in pediatric COVID-19 patients in the intensive care unit. Pediatric Nephrology 36, 2627-2638. doi: 10.1007/s00467-021-05084-x | Unrelated |
|  | Raju, R., Prajith, V., Biatris, P. S., and Sam Johnson Udaya Chander, J. (2021). Therapeutic role of corticosteroids in COVID-19: a systematic review of registered clinical trials. Future Journal of Pharmaceutical Sciences 7, 67. doi: 10.1186/s43094-021-00217-3 | Unrelated |
|  | Relph, S., Patel, T., Delaney, L., Sobhy, S., and Thangaratinam, S. (2021). Adverse pregnancy outcomes in women with diabetes-related microvascular disease and risks of disease progression in pregnancy: A systematic review and meta-analysis. PLoS Medicine 18, e1003856. doi: 10.1371/journal.pmed.1003856 | Unrelated |
|  | Rocha, K. O., Zanuncio, V. V., Freitas, B. A. C. d., and Lima, L. M. (2021). "COVID toes": A meta-analysis of case and observational studies on clinical, histopathological, and laboratory findings. Pediatric dermatology 38, 1143-1149. doi: 10.1111/pde.14805 | Unrelated |
|  | Rostami, A., Sepidarkish, M., Leeflang, M. M. G., Riahi, S. M., Nourollahpour Shiadeh, M., Esfandyari, S., Mokdad, A. H., Hotez, P. J., and Gasser, R. B. (2021). SARS-CoV-2 seroprevalence worldwide: a systematic review and meta-analysis. Clinical microbiology and infection: the official publication of the European Society of Clinical Microbiology and Infectious Diseases 27, 331-340. doi: 10.1016/j.cmi.2020.10.020 | Unrelated |
|  | Runacres, A., Mackintosh, K. A., Knight, R. L., Sheeran, L., Thatcher, R., Shelley, J., and McNarry, M. A. (2021). Impact of the covid-19 pandemic on sedentary time and behaviour in children and adults: A systematic review and meta-analysis. International Journal of Environmental Research and Public Health 18, 11286. doi: 10.3390/ijerph182111286 | Unrelated |
|  | Sah, P., Fitzpatrick, M. C., Zimmer, C. F., Abdollahi, E., Juden-Kelly, L., Moghadas, S. M., Singer, B. H., and Galvani, A. P. (2021). Asymptomatic SARS-CoV-2 infection: A systematic review and meta-analysis. Proceedings of the National Academy of Sciences of the United States of America 118, e2109229118. doi: 10.1073/pnas.2109229118 | Unrelated |
|  | Salem, D., Katranji, F., and Bakdash, T. (2021). COVID-19 infection in pregnant women: Review of maternal and fetal outcomes. International Journal of Gynecology and Obstetrics 152, 291-298. doi: 10.1002/ijgo.13533 | Unrelated |
|  | Sarastry, R., Layarta, C., Aladini, U., and Pramono, B. A. (2021). Delivery routes in pregnancy with covid-19 and the risk of intrapartum vertical transmission: A meta-analysis. Medical Journal of Indonesia 30, 116-122. doi: 10.13181/mji.oa.214779 | Unrelated |
|  | Sathian, B., Asim, M., Banerjee, I., Roy, B., Pizarro, A. B., Mancha, M. A., Van Teijlingen, E. R., Kord-Varkaneh, H., Mekkodathil, A. A., Subramanya, S. H., Do Nascimento, I. J. B., Antony, N., Menezes, R. G., Simkhada, P., and Al Hamad, H. (2021). Development and implementation of a potential coronavirus disease 2019 (COVID-19) vaccine: A systematic review and meta-analysis of vaccine clinical trials. Nepal Journal of Epidemiology 11, 959-982. doi: 10.3126/nje.v11i1.36163 | Unrelated |
|  | Sengupta, P., Leisegang, K., and Agarwal, A. (2021). The impact of COVID-19 on the male reproductive tract and fertility: A systematic review. Arab Journal of Urology 19, 423-436. doi: 10.1080/2090598X.2021.1955554 | Unrelated |
|  | Shao, S., Wang, Y., Kang, H., and Tong, Z. (2021). Effect of convalescent blood products for patients with severe acute respiratory infections of viral etiology: A systematic review and meta-analysis. International Journal of Infectious Diseases 102, 397-411. doi: 10.1016/j.ijid.2020.09.1443 | Unrelated |
|  | Sharif, N., Alzahrani, K. J., Ahmed, S. N., and Dey, S. K. (2021). Efficacy, Immunogenicity and Safety of COVID-19 Vaccines: A Systematic Review and Meta-Analysis. Frontiers in immunology 12, 714170. doi: 10.3389/fimmu.2021.714170 | Unrelated |
|  | Sheikh, J., Lawson, H., Kew, T., Ansari, K., Chatterjee, S., Dixit, A., Zhou, D., Balaji, R., Clave Llavall, A., Yap, M., Debenham, L., Coomar, D., Yuan, M., Qiu, X., Stallings, E., Allotey, J., Bonet, M., Zamora, J., and Thangaratinam, S. (2021). COVID-19 maternal and perinatal outcomes: Living systematic review and meta-analysis. BJOG: An International Journal of Obstetrics and Gynaecology 128, 192-193. doi: 10.1111/1471-0528.15-16715 | Unrelated |
|  | Shi, M., Stelick, A., Licker, S., and Dando, R. (2021). On the validity of longitudinal comparisons of central location consumer testing results prior to COVID-19 versus home use testing data during the pandemic. Journal of food science 86, 4668-4677. doi: 10.1111/1750-3841.15905 | Unrelated |
|  | Shi, S., Wang, F., Li, J., Li, Y., Li, W., Wu, X., Kou, S., Wu, Y., Wang, X., Pei, C., Huang, D., Qiu, H., Wang, P., and Wang, Z. (2021). The effect of Chinese herbal medicine on digestive system and liver functions should not be neglected in COVID-19: An updated systematic review and meta-analysis. IUBMB life 73, 739-760. doi: 10.1002/iub.2467 | Unrelated |
|  | Singh, A., Hussain, S., and Antony, B. (2021). Non-alcoholic fatty liver disease and clinical outcomes in patients with COVID-19: A comprehensive systematic review and meta-analysis. Diabetes & metabolic syndrome 15, 813-822. doi: 10.1016/j.dsx.2021.03.019 | Unrelated |
|  | Singh, H., Kaur, H., Singh, K., and Sen, C. K. (2021). Cutaneous manifestations of COVID-19: A systematic review. Advances in Wound Care 10, 51-80. doi: 10.1089/wound.2020.1309 | Unrelated |
|  | Singh, R. K., Bajpai, R., and Kaswan, P. (2021). COVID-19 pandemic and psychological wellbeing among health care workers and general population: A systematic-review and meta-analysis of the current evidence from India. Clinical Epidemiology and Global Health 11, 100737. doi: 10.1016/j.cegh.2021.100737 | Unrelated |
|  | Siristatidis, H., Papapanou, M., Papaioannou, M., Petta, A., Routsi, E., Farmaki, M., and Nikolaos, V. (2021). Maternal and neonatal characteristics and outcomes of COVID-19 from early pregnancy until labor: an overview of systematic reviews. Human Reproduction 36, i110-i111. doi: 10.1093/humrep/deab128.008 | Unrelated |
|  | Sitek, A., Ade, J., Pitlick, M. M., Chiarella, S., Divekar, R., Iyer, V., Wang, Z., and Joshi, A. (2021). COVID-19-Associated Hospitalization and Outcomes in Patients with Asthma: A Systematic Review and Meta-analysis. Journal of Allergy and Clinical Immunology 147, AB243. doi: 10.1016/j.jaci.2020.12.032 | Unrelated |
|  | Sitek, A. N., Ade, J. M., Chiarella, S. E., Divekar, R. D., Pitlick, M. M., Iyer, V. N., Wang, Z., and Joshi, A. Y. (2021). Outcomes among patients with COVID-19 and asthma: A systematic review and meta-analysis. Allergy and Asthma Proceedings 42, 267-273. doi: 10.2500/aap.2021.42.210041 | Unrelated |
|  | Slamang, W., Abdullahi, L. H., Mustafa, F., Harrison, M. J. T., Butters, C. M., Deakin, C. T., Scott, C., and Webb, K. (2021). A rapid review and meta-analysis of the laboratory phenotype of multisystem inflammatory syndrome in children. Pediatric Rheumatology 19. doi: 10.1186/s12969-021-00632-z | Unrelated |
|  | Song, W. M., Zhao, J. Y., Zhang, Q. Y., Liu, S. Q., Zhu, X. H., An, Q. Q., Xu, T. T., Li, S. J., Liu, J. Y., Tao, N. N., Liu, Y., Li, Y. F., and Li, H. C. (2021). COVID-19 and Tuberculosis Coinfection: An Overview of Case Reports/Case Series and Meta-Analysis. Frontiers in Medicine 8, 657006. doi: 10.3389/fmed.2021.657006 | Unrelated |
|  | Struyf, T., Deeks, J. J., Dinnes, J., Takwoingi, Y., Davenport, C., Leeflang, M. M., Spijker, R., Hooft, L., Emperador, D., Domen, J., Horn, S. R. A., and Van den Bruel, A. (2021). Signs and symptoms to determine if a patient presenting in primary care or hospital outpatient settings has COVID-19. The Cochrane database of systematic reviews 2, CD013665. doi: 10.1002/14651858.CD013665.pub2 | Unrelated |
|  | Sullivan, B. N., and Fischer, T. (2021). Age-Associated Neurological Complications of COVID-19: A Systematic Review and Meta-Analysis. Frontiers in Aging Neuroscience 13, 653694. doi: 10.3389/fnagi.2021.653694 | Unrelated |
|  | Takagi, H., Kuno, T., Yokoyama, Y., Ueyama, H., Matsushiro, T., Hari, Y., and Ando, T. (2021). Meta-regression of COVID-19 prevalence/fatality on socioeconomic characteristics of data from top 50 U.S. large cities. Journal of medical virology 93, 595-598. doi: 10.1002/jmv.26335 | Unrelated |
|  | Teoh, S. E., Masuda, Y., Tan, D. J. H., Liu, N., Morrison, L. J., Ong, M. E. H., Blewer, A. L., and Ho, A. F. W. (2021). Impact of the COVID-19 pandemic on the epidemiology of out-of-hospital cardiac arrest: a systematic review and meta-analysis. Annals of Intensive Care 11, 169. doi: 10.1186/s13613-021-00957-8 | Unrelated |
|  | Thompson, H. A., Mousa, A., Dighe, A., Fu, H., Arnedo-Pena, A., Barrett, P., Bellido-Blasco, J., Bi, Q., Caputi, A., Chaw, L., De Maria, L., Hoffmann, M., Mahapure, K., Ng, K., Raghuram, J., Singh, G., Soman, B., Valent, F., Vimercati, L., Wee, L. E., Wong, J., Ghani, A. C., and Ferguson, N. M. (2021). Severe Acute Respiratory Syndrome Coronavirus 2 (SARS-CoV-2) Setting-specific Transmission Rates: A Systematic Review and Meta-analysis. Clinical Infectious Diseases 73, E754-E764. doi: 10.1093/cid/ciab100 | Unrelated |
|  | Tolu, L. B., Ezeh, A., and Feyissa, G. T. (2021). Vertical transmission of Severe Acute Respiratory Syndrome Coronavirus 2: A scoping review. PloS one 16, e0250196. doi: 10.1371/journal.pone.0250196 | Unrelated |
|  | Topriceanu, C.-C., Wong, A., Moon, J. C., Hughes, A. D., Bann, D., Chaturvedi, N., Patalay, P., Conti, G., and Captur, G. (2021). Evaluating access to health and care services during lockdown by the COVID-19 survey in five UK national longitudinal studies. BMJ open 11, e045813. doi: 10.1136/bmjopen-2020-045813 | Unrelated |
|  | Topriceanu, C.-C., Wong, A., Moon, J. C., Hughes, A. D., Chaturvedi, N., Conti, G., Bann, D., Patalay, P., and Captur, G. (2021). Impact of lockdown on key workers: findings from the COVID-19 survey in four UK national longitudinal studies. Journal of epidemiology and community health 75, 955-962. doi: 10.1136/jech-2020-215889 | Unrelated |
|  | Vaccaro, C., Mahmoud, F., Aboulatta, L., Aloud, B., and Eltonsy, S. (2021). The impact of COVID-19 first wave national lockdowns on perinatal outcomes: a rapid review and meta-analysis. BMC Pregnancy and Childbirth 21, 676. doi: 10.1186/s12884-021-04156-y | Unrelated |
|  | Vainio, P. J., Hietasalo, P., Koivisto, A.-L., Kaariainen, S., Turunen, J., Virtala, M., Vuorinen, J., and Scheinin, M. (2021). Hydroxychloroquine in the treatment of adult patients with Covid-19 infection in a primary care setting (LIBERTY): A structured summary of a study protocol for a randomised controlled trial. Trials 22, 44. doi: 10.1186/s13063-020-04989-6 | Unrelated |
|  | Vaselli, N. M., Hungerford, D., Shenton, B., Khashkhusha, A., Cunliffe, N. A., and French, N. (2021). The seroprevalence of SARS-CoV-2 during the first wave in Europe 2020: A systematic review. PLoS ONE 16, e0250541. doi: 10.1371/journal.pone.0250541 | Unrelated |
|  | Vassilopoulou, E., Feketea, G., Koumbi, L., Mesiari, C., Berghea, E. C., and Konstantinou, G. N. (2021). Breastfeeding and COVID-19: From Nutrition to Immunity. Frontiers in immunology 12, 661806. doi: 10.3389/fimmu.2021.661806 | Unrelated |
|  | Vlieg-Boerstra, B., De Jong, N., Meyer, R., Agostini, C., De Cosmi, V., Grimshaw, K., Paolo Milani, G., Muraro, A., Oude Elberink, H., Pali-Scholl, I., Roduit, C., Sasaki, M., Skypala, I., Solokowska, M., Van Splunter, M., Untersmayer, E., Venter, C., and O'Mahony, L. (2021). Nutrient supplementation for primary prevention of respiratory viral infections in healthy subjects: An EAACI systematic review and meta-analysis. Allergy: European Journal of Allergy and Clinical Immunology 76, 649-650. doi: 10.1111/all.15098 | Unrelated |
|  | Voysey, M., Costa Clemens, S. A., Madhi, S. A., Weckx, L. Y. (2021). Single-dose administration and the influence of the timing of the booster dose on immunogenicity and efficacy of ChAdOx1 nCoV-19 (AZD1222) vaccine: a pooled analysis of four randomised trials. Lancet (London, England) 397, 881-891. doi: 10.1016/S0140-6736(21)00432-3 | Unrelated |
|  | Wang, Y., Ao, G., Qi, X., and Ma, M. (2021). The relationship between severe or dead COVID-19 and asthma: A meta-analysis. Clinical and experimental allergy: journal of the British Society for Allergy and Clinical Immunology 51, 354-359. doi: 10.1111/cea.13773 | Unrelated |
|  | Wang, Y., Huo, P., Dai, R., Lv, X., Yuan, S., Zhang, Y., Guo, Y., Li, R., Yu, Q., and Zhu, K. (2021). Convalescent plasma may be a possible treatment for COVID-19: A systematic review. International Immunopharmacology 91, 107262. doi: 10.1016/j.intimp.2020.107262 | Unrelated |
|  | Wang, Y. H., Wu, C. C., Bai, C. H., Lu, S. C., Yang, Y. P., Lin, Y. Y., Lai, W. Y., Lin, T. W., Jheng, Y. C., Lee, M. C., and Chen, C. C. (2021). Evaluation of the diagnostic accuracy of COVID-19 antigen tests: A systematic review and meta-analysis. Journal of the Chinese Medical Association 84, 1028-1037. doi: 10.1097/JCMA.0000000000000626 | Unrelated |
|  | Wei, S. Q., Bilodeau-Bertrand, M., Liu, S., and Auger, N. (2021). The impact of COVID-19 on pregnancy outcomes: a systematic review and meta-analysis. CMAJ: Canadian Medical Association journal = journal de l'Association medicale canadienne 193, E540-E548. doi: 10.1503/cmaj.202604 | Unrelated |
|  | Wels, J., Booth, C., Wielgoszewska, B., Green, M., Di Gessa, G., Huggins, C. F., Griffith, G. J., Kwong, A. S. F., Bowyer, R. C. E., Maddock, J., Patalay, P., Silverwood, R. J., Fitzsimons, E., Shaw, R., Thompson, E. J., Steptoe, A., Hughes, A., Chaturvedi, N., Steves, C. J., Katikireddi, S. V., and Ploubidis, G. B. (2021). Mental and social wellbeing and the UK Coronavirus Job Retention Scheme: Evidence from nine longitudinal studies. medRxiv. doi: 10.1101/2021.11.15.21266264 | Unrelated |
|  | Wijarnpreecha, K., Ungprasert, P., Panjawatanan, P., Harnois, D. M., Zaver, H. B., Ahmed, A., and Kim, D. (2021). COVID-19 and liver injury: A meta-analysis. European Journal of Gastroenterology and Hepatology 33, 990-995. doi: 10.1097/MEG.0000000000001817 | Unrelated |
|  | Yan, D., Zhang, X., Chen, C., Jiang, D., Liu, X., Huang, C., Zhou, Y., Guan, Z., Ding, C., Chen, L., Lan, L., Fu, X., Wu, J., Li, L., and Yang, S. (2021). Characteristics of Viral Shedding Time in SARS-CoV-2 Infections: A Systematic Review and Meta-Analysis. Frontiers in public health 9, 652842. doi: 10.3389/fpubh.2021.652842 | Unrelated |
|  | Yang, J., D'Souza, R., Kharrat, A., Fell, D. B., Snelgrove, J. W., Murphy, K. E., and Shah, P. S. (2021). COVID-19 pandemic and population-level pregnancy and neonatal outcomes: a living systematic review and meta-analysis. Acta Obstetricia et Gynecologica Scandinavica 100, 1756-1770. doi: 10.1111/aogs.14206 | Unrelated |
|  | Zhang, C., Chu, H., Pei, Y. V., and Zhang, J. (2021). Laboratory Effects of COVID-19 Infection in Pregnant Women and Their Newborns: A Systematic Review and Meta-Analysis. Frontiers in global women's health 2, 647072. doi: 10.3389/fgwh.2021.647072 | Unrelated |
|  | Zhang, Q., Lu, H., Li, F., Li, X., Wang, T., Yang, Q., and Mi, L. (2021). The impact of COVID-19 on sexual behaviors of young women and men: A protocol for systematic review and meta-analysis. Medicine 100, e24415. doi: 10.1097/MD.0000000000024415 | Unrelated |
|  | Zhang, T., Ding, S., Zeng, Z., Cheng, H., Zhang, C., Mao, X., Pan, H., Xia, G., and Che, D. (2021). Estimation of Incubation Period and Serial Interval for SARS-CoV-2 in Jiangxi, China, and an Updated Meta-Analysis. Journal of infection in developing countries 15, 326-332. doi: 10.3855/jidc.14025 | Unrelated |
|  | Zhao, Y. J., Jin, Y., Rao, W. W., Li, W., Zhao, N., Cheung, T., Ng, C. H., Wang, Y. Y., Zhang, Q. E., and Xiang, Y. T. (2021). The prevalence of psychiatric comorbidities during the SARS and COVID-19 epidemics: a systematic review and meta-analysis of observational studies. Journal of Affective Disorders 287, 145-157. doi: 10.1016/j.jad.2021.03.016 | Unrelated |
|  | Zhu, F., Zozaya, C., Zhou, Q., De Castro, C., and Shah, P. S. (2021). SARS-CoV-2 genome and antibodies in breastmilk: A systematic review and meta-analysis. Archives of Disease in Childhood: Fetal and Neonatal Edition 106, F514-F521. doi: 10.1136/archdischild-2020-321074 | Unrelated |
|  | Abara, W. E., Gee, J., Delorey, M., Tun, Y., Mu, Y., Shay, D. K., and Shimabukuro, T. (2022). Expected Rates of Select Adverse Events After Immunization for Coronavirus Disease 2019 Vaccine Safety Monitoring. Journal of Infectious Diseases 225, 1569-1574. doi: 10.1093/infdis/jiab628 | Unrelated |
|  | Abdelmoneim, S. A., Sallam, M., Hafez, D. M., Elrewany, E., Mousli, H. M., Hammad, E. M., Elkhadry, S. W., Adam, M. F., Ghobashy, A. A., Naguib, M., Nour El-Deen, A. E. S., Aji, N., and Ghazy, R. M. (2022). COVID-19 Vaccine Booster Dose Acceptance: Systematic Review and Meta-Analysis. Tropical Medicine and Infectious Disease 7, 298. doi: 10.3390/tropicalmed7100298 | Unrelated |
|  | Abounoori, M., Aghajani, A., Chaibakhsh, S., Babakhanian, M., Pourazizi, M., and Peyman, A. (2022). Paediatric myopia shift during the COVID-19 pandemic home quarantine: a systematic review and meta-analysis. BMJ paediatrics open 6. doi: 10.1136/bmjpo-2022-001755 | Unrelated |
|  | Acuff, S. F., Strickland, J. C., Tucker, J. A., and Murphy, J. G. (2022). Changes in alcohol use during COVID-19 and associations with contextual and individual difference variables: A systematic review and meta-analysis. Psychology of addictive behaviors: journal of the Society of Psychologists in Addictive Behaviors 36, 1-19. doi: 10.1037/adb0000796 | Unrelated |
|  | Ahmed, S. K., Mohamed, M. G., Essa, R. A., Ahmed Rashad, E. A., Ibrahim, P. K., Khdir, A. A., and Wsu, Z. H. (2022). Global reports of myocarditis following COVID-19 vaccination: A systematic review and meta-analysis. Diabetes and Metabolic Syndrome: Clinical Research and Reviews 16, 102513. doi: 10.1016/j.dsx.2022.102513 | Unrelated |
|  | Aho Glele, L. S., Simon, E., Bouit, C., Serrand, M., Filipuzzi, L., Astruc, K., Kadhel, P., and Sagot, P. (2022). Association between SARS-Cov-2 infection during pregnancy and adverse pregnancy outcomes: A re-analysis of the data reported by Wei et al. (2021). Infectious diseases now 52, 123-128. doi: 10.1016/j.idnow.2022.02.009 | Unrelated |
|  | Akbarialiabad, H., Shidhaye, R., Shidhaye, P., Cuijpers, P., Weaver, M. R., Bahrololoom, M., Kiburi, S., Njuguna, I. N., Taghrir, M. H., and Kumar, M. (2022). Impact of major disease outbreaks in the third millennium on adolescent and youth sexual and reproductive health and rights in low and/or middle-income countries: a systematic scoping review protocol. BMJ open 12, e051216. doi: 10.1136/bmjopen-2021-051216 | Unrelated |
|  | Al-Ajlouni, Y. A., Al Ta'ani, O., Shamaileh, G., Mushasha, R., Makarem, N., and Duncan, D. T. (2022). Effects of the COVID-19 pandemic on sleep health among Middle Eastern and North African (MENA) populations: a systematic review of the literature. BMJ open 12, e066964. doi: 10.1136/bmjopen-2022-066964 | Unrelated |
|  | Al-Jammali, S., Al-Zakhari, R., Sheets, N., Mahtani, A., Stefanishina, V., and Isber, N. (2022). Bradyarrhythmia After Remdesivir Administration in SARSCoV-2: A Review of Literature and Meta-Analysis of Observational Studies in Epidemiology. Cardiology Research 13, 135-143. doi: 10.14740/cr1377 | Unrelated |
|  | Alhumaid, S., Al Mutair, A., Alali, J., Al Dossary, N., Albattat, S. H., Al HajjiMohammed, S. M., Almuaiweed, F. S., AlZaid, M. R., Alomran, M. J., Alqurini, Z. S., Alsultan, A. A., Alhajji, T. S., Alshaikhnasir, S. M., Al Motared, A., Al Mutared, K. M., Hajissa, K., and Rabaan, A. A. (2022). Efficacy and Safety of Tixagevimab/Cilgavimab to Prevent COVID-19 (Pre-Exposure Prophylaxis): A Systematic Review and Meta-Analysis. Diseases (Basel, Switzerland) 10. doi: 10.3390/diseases10040118 | Unrelated |
|  | Allotey, J., Chatterjee, S., Kew, T., Gaetano, A., Stallings, E., Fernandez-Garcia, S., Yap, M., Sheikh, J., Lawson, H., Coomar, D., Dixit, A., Zhou, D., Balaji, R., Littmoden, M., King, Y., Debenham, L., Llavall, A. C., Ansari, K., Sandhu, G., Banjoko, A., Walker, K., O'Donoghue, K., van Wely, M., van Leeuwen, E., Kostova, E., Kunst, H., Khalil, A., Brizuela, V., Broutet, N., Kara, E., Kim, C. R., Thorson, A., Oladapo, O. T., Zamora, J., Bonet, M., Mofenson, L., and Thangaratinam, S. (2022). SARS-CoV-2 positivity in offspring and timing of mother-to-child transmission: living systematic review and meta-analysis. BMJ (Clinical research ed.) 376, e067696. doi: 10.1136/bmj-2021-067696 | Unrelated |
|  | Andreas, M., Iannizzi, C., Bohndorf, E., Monsef, I., Piechotta, V., Meerpohl, J. J., and Skoetz, N. (2022). Interventions to increase COVID-19 vaccine uptake: a scoping review. Cochrane Database of Systematic Reviews 2022, CD015270. doi: 10.1002/14651858.CD015270 | Unrelated |
|  | Anonymous (2022). "Changes in alcohol use during COVID-19 and associations with contextual and individual difference variables: A systematic review and meta-analysis." Correction to Acuff et al. (2022). Psychology of addictive behaviors: journal of the Society of Psychologists in Addictive Behaviors 36, 386. doi: 10.1037/adb0000852 | Unrelated |
|  | Bailey, A. J. M., Kirkham, A. M., Monaghan, M., Shorr, R., Buchan, C. A., Bredeson, C., and Allan, D. S. (2022). A Portrait of SARS-CoV-2 Infection in Patients Undergoing Hematopoietic Cell Transplantation: A Systematic Review of the Literature. Current oncology (Toronto, Ont.) 29, 337-349. doi: 10.3390/curroncol29010030 | Unrelated |
|  | Bakaloudi, D. R., Barazzoni, R., Bischoff, S. C., Breda, J., Wickramasinghe, K., and Chourdakis, M. (2022). Impact of the first COVID-19 lockdown on body weight: A combined systematic review and a meta-analysis. Clinical Nutrition 41, 3046-3054. doi: 10.1016/j.clnu.2021.04.015 | Unrelated |
|  | Balachandren, N., Davies, M. C., Hall, J. A., Stephenson, J. M., David, A. L., Barrett, G., O'Neill, H. C., Ploubidis, G. B., Yasmin, E., and Mavrelos, D. (2022). SARS-CoV-2 infection in the first trimester and the risk of early miscarriage: a UK population-based prospective cohort study of 3041 pregnancies conceived during the pandemic. Human reproduction (Oxford, England) 37, 1126-1133. doi: 10.1093/humrep/deac062 | Unrelated |
|  | Banchelli, F., Negro, P., Guido, M., D'Amico, R., Fittipaldo, V. A., Grima, P., and Zizza, A. (2022). The Role of ABO Blood Type in Patients with SARS-CoV-2 Infection: A Systematic Review. Journal of Clinical Medicine 11, 3029. doi: 10.3390/jcm11113029 | Unrelated |
|  | Baysan, C., Col, M., Aydin, S., Gurbuz, S., Ozdemir, C., Bekar, T., and Buyukdemirci, E. (2022). Assessment of Features of MIS-C and Non-MIS-C Patients: Meta-Analysis. Flora 27, 158-176. doi: 10.5578/flora.20229912 | Unrelated |
|  | Bergeri, I., Whelan, M. G., Ware, H., Subissi, L., Nardone, A., Lewis, H. C., Li, Z., Ma, X., Valenciano, M., Cheng, B., Al Ariqi, L., Rashidian, A., Okeibunor, J., Azim, T., Wijesinghe, P., Le, L.-V., Vaughan, A., Pebody, R., Vicari, A., Yan, T., Yanes-Lane, M., Cao, C., Clifton, D. A., Cheng, M. P., Papenburg, J., Buckeridge, D., Bobrovitz, N., Arora, R. K., and Van Kerkhove, M. D. (2022). Global SARS-CoV-2 seroprevalence from January 2020 to April 2022: A systematic review and meta-analysis of standardized population-based studies. PLoS medicine 19, e1004107. doi: 10.1371/journal.pmed.1004107 | Unrelated |
|  | Chang, X., Li, S., Fu, Y., Dang, H., and Liu, C. (2022). Safety and efficacy of corticosteroids in ARDS patients: a systematic review and meta-analysis of RCT data. Respiratory Research 23, 301. doi: 10.1186/s12931-022-02186-4 | Unrelated |
|  | Charide, R., Stallwood, L., Munan, M., Sayfi, S., Hartling, L., Butcher, N. J., Offringa, M., Elliott, S., Richards, D. P., Mathew, J. L., Akl, E. A., Kredo, T., Mbuagbaw, L., Motillal, A., Baba, A., Prebeg, M., Relihan, J., Scott, S. D., Suvada, J., Falavigna, M., Klugar, M., Lotfi, T., Stevens, A., Pottie, K., and Schunemann, H. J. (2022). Knowledge mobilization activities to support decision-making by youth, parents, and adults using a systematic and living map of evidence and recommendations on COVID-19: protocol for three randomized controlled trials and qualitative user-experience studies. medRxiv. doi: 10.1101/2022.05.09.22274842 | Unrelated |
|  | Chen, Q., Li, W., Xiong, J., and Zheng, X. (2022). Prevalence and Risk Factors Associated with Postpartum Depression during the COVID-19 Pandemic: A Literature Review and Meta-Analysis. International Journal of Environmental Research and Public Health 19, 2219. doi: 10.3390/ijerph19042219 | Unrelated |
|  | Chen, Y., Wang, J., Geng, Y., Fang, Z., Zhu, L., Chen, Y., and Yao, Y. (2022). Meta-analysis of the prevalence of anxiety and depression among frontline healthcare workers during the COVID-19 pandemic. Frontiers in public health 10, 984630. doi: 10.3389/fpubh.2022.984630 | Unrelated |
|  | Cheng, Q., Zhao, G., Chen, J., Jia, Q., and Fang, Z. (2022). Comparative efficacy and safety of pharmacological interventions for severe COVID-19 patients: An updated network meta-analysis of 48 randomized controlled trials. Medicine (United States) 101, E30998. doi: 10.1097/MD.0000000000030998 | Unrelated |
|  | Cho, H., Myung, S. K., and Cho, H. E. (2022). Efficacy of Vitamin D Supplements in Treatment of Acute Respiratory Infection: A Meta-Analysis for Randomized Controlled Trials. Nutrients 14, 1144. doi: 10.3390/nu14061144 | Unrelated |
|  | Choi, J. H., Choi, S.-H., and Yun, K. W. (2022). Risk Factors for Severe COVID-19 in Children: A Systematic Review and Meta-Analysis. Journal of Korean medical science doi: 10.3346/jkms.2022.37.e35 | Unrelated |
|  | Christensen, J., O'Callaghan, K., Sinclair, H., Hawke, K., Love, A., Hajkowicz, K., and Stewart, A. G. (2022). Risk factors, treatment and outcomes of subacute thyroiditis secondary to COVID-19: a systematic review. Internal medicine journal 52, 522-529. doi: 10.1111/imj.15432 | Unrelated |
|  | Chu, D. K., Brignardello-Petersen, R., Guyatt, G. H., Ricci, C., and Genuneit, J. (2022). Method's corner: Allergist's guide to network meta-analysis. Pediatric allergy and immunology: official publication of the European Society of Pediatric Allergy and Immunology 33, e13609. doi: 10.1111/pai.13609 | Unrelated |
|  | Claussen, A. H., Holbrook, J. R., Hutchins, H. J., Robinson, L. R., Bloomfield, J., Meng, L., Bitsko, R. H., O'Masta, B., Cerles, A., Maher, B., Rush, M., and Kaminski, J. W. (2022). All in the Family? A Systematic Review and Meta-analysis of Parenting and Family Environment as Risk Factors for Attention-Deficit/Hyperactivity Disorder (ADHD) in Children. Prevention science: the official journal of the Society for Prevention Research. doi: 10.1007/s11121-022-01358-4 | Unrelated |
|  | Collatuzzo, G., Visci, G., Violante, F. S., Porru, S., Spiteri, G., Monaco, M. G. L., Larese Fillon, F., Negro, C., Janke, C., Castelletti, N., De Palma, G., Sansone, E., Mates, D., Teodorescu, S., Fabianova, E., Beresova, J., Vimercati, L., Tafuri, S., Abedini, M., Ditano, G., Asafo, S. S., and Boffetta, P. (2022). Determinants of anti-S immune response at 6 months after COVID-19 vaccination in a multicentric European cohort of healthcare workers - ORCHESTRA project. Frontiers in immunology 13, 986085. doi: 10.3389/fimmu.2022.986085 | Unrelated |
|  | Currie, C. L., Larouche, R., Lauren Voss, M., Trottier, M., Spiwak, R., Higa, E., Scott, D. R., and Tallow, T. (2022). Effectiveness of Live Health Professional-Led Group eHealth Interventions for Adult Mental Health: Systematic Review of Randomized Controlled Trials. Journal of Medical Internet Research 24, e27939. doi: 10.2196/27939 | Unrelated |
|  | De Bruin, O., Engjom, H., Vousden, N., Knight, M., Donati, S., Aabakke, A., Ayras, O., Overtoom, E., Vercoutere, A., Jonasdottir, E., and Bloemenkamp, K. (2022). COVID-19 infection and medications in pregnancy: pooled analysis of INOSS population-based studies. BJOG: An International Journal of Obstetrics and Gynaecology 129, 143-144. doi: 10.1111/1471-0528.18_17178 | Unrelated |
|  | De Matteis, S., Forastiere, F., Baldacci, S., Maio, S., Tagliaferro, S., Fasola, S., Cilluffo, G., La Grutta, S., and Viegi, G. (2022). Issue 1 - "Update on adverse respiratory effects of outdoor air pollution". Part 1): Outdoor air pollution and respiratory diseases: A general update and an Italian perspective. Pulmonology 28, 284-296. doi: 10.1016/j.pulmoe.2021.12.008 | Unrelated |
|  | de Souza Santos, D., Bittencourt, E. A., de Moraes Malinverni, A. C., Kisberi, J. B., de Franca Vilaca, S., and Iwamura, E. S. M. (2022). Domestic violence against women during the Covid-19 pandemic: A scoping review. Forensic Science International: Reports 5, 100276. doi: 10.1016/j.fsir.2022.100276 | Unrelated |
|  | Delanerolle, G., McCauley, M., Hirsch, M., Zheng, Y., Cong, X., Cavalini, H., Shetty, A., Rathod, S., Shi, J. Q., Hapangama, D. K., and Phiri, P. (2022). The prevalence of mental ill-health in women during pregnancy and after childbirth during the Covid-19 pandemic: a Systematic review and Meta-analysis. medRxiv. doi: 10.1101/2022.06.13.22276327 | Unrelated |
|  | Deng, J., Ma, Y., Liu, Q., Du, M., Liu, M., and Liu, J. (2022). Association of Infection with Different SARS-CoV-2 Variants during Pregnancy with Maternal and Perinatal Outcomes: A Systematic Review and Meta-Analysis. International Journal of Environmental Research and Public Health 19, 15932. doi: 10.3390/ijerph192315932 | Unrelated |
|  | Dhar, D., Dey, T., Samim, M. M., Padmanabha, H., Chatterjee, A., Naznin, P., Chandra, S. R., Mallesh, K., Shah, R., Siddiqui, S., Pratik, K., Ameya, P., and Abhishek, G. (2022). Systemic inflammatory syndrome in COVID-19-SISCoV study: systematic review and meta-analysis. Pediatric Research 91, 1334-1349. doi: 10.1038/s41390-021-01545-z | Unrelated |
|  | El-Qushayri, A. E., Mahmoud, M. A., Salman, S., Sarsik, S., and Nardone, B. (2022). Dupilumab therapy in atopic dermatitis is safe during COVID-19 infection era: A systematic review and meta-analysis of 1611 patients. Dermatologic Therapy 35, e15476. doi: 10.1111/dth.15476 | Unrelated |
|  | Emerson, E., Aitken, Z., Totsika, V., King, T., Stancliffe, R. J., Hatton, C., Llewellyn, G., Hastings, R. P., and Kavanagh, A. (2022). The impact of the COVID pandemic on working age adults with disability: Meta-analysis of evidence from four national surveys. Health & social care in the community 30, e4758-e4769. doi: 10.1111/hsc.13882 | Unrelated |
|  | Endalew, M., Belay, D. G., Tsega, N. T., Aragaw, F. M., Gashaw, M., and Asratie, M. H. (2022). Limited handwashing facility and associated factors in sub-Saharan Africa: pooled prevalence and multilevel analysis of 29 sub-Saharan Africa countries from demographic health survey data. BMC public health 22, 1969. doi: 10.1186/s12889-022-14390-4 | Unrelated |
|  | Fajar, J. K., Sallam, M., Soegiarto, G., Sugiri, Y. J., Anshory, M., Wulandari, L., Kosasih, S. A. P., Ilmawan, M., Kusnaeni, K., Fikri, M., Putri, F., Hamdi, B., Pranatasari, I. D., Aina, L., Maghfiroh, L., Ikhriandanti, F. S., Endiaverni, W. O., Nugraha, K. W., Wiranudirja, O., Edinov, S., Hamdani, U., Rosyidah, L., Lubaba, H., Ariwibowo, R., Andistyani, R., Fitriani, R., Hasanah, M., Nafis, F. A. D., Tamara, F., Latamu, F. O., Kusuma, H. I., Rabaan, A. A., Alhumaid, S., Mutair, A. A., Garout, M., Halwani, M. A., Alfaresi, M., Al Azmi, R., Alasiri, N. A., Alshukairi, A. N., Dhama, K., and Harapan, H. (2022). Global Prevalence and Potential Influencing Factors of COVID-19 Vaccination Hesitancy: A Meta-Analysis. Vaccines 10, 1356. doi: 10.3390/vaccines10081356 | Unrelated |
|  | Feduniw, S., Modzelewski, J., Kajdy, A., Sys, D., Kwiatkowski, S., Makomaska-Szaroszyk, E., and Rabijewski, M. (2022). Anxiety of pregnant women in time of catastrophic events, including COVID-19 pandemic: a systematic review and meta-analysis. Journal of psychosomatic obstetrics and gynaecology 43, 400-410. doi: 10.1080/0167482X.2021.1985453 | Unrelated |
|  | Fekih-Romdhane, F., Pandi-Perumal, S. R., Conus, P., Krebs, M.-O., Cheour, M., Seeman, M. V., and Jahrami, H. A. (2022). Prevalence and risk factors of self-reported psychotic experiences among high school and college students: A systematic review, meta-analysis, and meta-regression. Acta psychiatrica Scandinavica 146, 492-514. doi: 10.1111/acps.13494 | Unrelated |
|  | Freeman, V., Hughes, S., Carle, C., Campbell, D., Egger, S., Hui, H., Yap, S., Deandrea, S., Caruana, M., Onyeka, T. C., Ijzerman, M. J., Ginsburg, O., Bray, F., Sullivan, R., Aggarwal, A., Peacock, S. J., Chan, K. K. W., Hanna, T. P., Soerjomataram, I., O'Connell, D. L., Steinberg, J., and Canfell, K. (2022). Are patients with cancer at higher risk of COVID-19-related death? A systematic review and critical appraisal of the early evidence. Journal of cancer policy 33, 100340. doi: 10.1016/j.jcpo.2022.100340 | Unrelated |
|  | Fujita-Rohwerder, N., Beckmann, L., Zens, Y., and Verma, A. (2022). Diagnostic accuracy of rapid point-of-care tests for diagnosis of current SARS-CoV-2 infections in children: A systematic review and meta-analysis. BMJ Evidence-Based Medicine, 111828. doi: 10.1136/bmjebm-2021-111828 | Unrelated |
|  | Glessner, J. T., Chang, X., Mentch, F., Qu, H., Abrams, D. J., Thomas, A., Sleiman, P. M. A., and Hakonarson, H. (2022). COVID-19 in pediatrics: Genetic susceptibility. Frontiers in Genetics 13, 928466. doi: 10.3389/fgene.2022.928466 | Unrelated |
|  | Goncalves Cerejeira, J., Vallecillo Adame, C., Uribe, S., Santos Carrasco, I., Jimenez Aparicio, T., De Andres Lobo, C., Queipo De Llano De La Viuda, M., Gonzaga Ramirez, A., and Guerra Valera, G. (2022). Covid-19 pandemic and suicidal risk among adolescents. European Psychiatry 65, S436. doi: 10.1192/j.eurpsy.2022.1107 | Unrelated |
|  | Gudipudi, R., Jungbauer, W. N., Brennan, E., and Pecha, P. P. (2022). Parent and provider satisfaction of telehealth in pediatric surgical subspecialty care: A systematic review. Journal of telemedicine and telecare, 1357633X221110368. doi: 10.1177/1357633X221110368 | Unrelated |
|  | Harwood, R., Yan, H., Talawila Da Camara, N., Smith, C., Ward, J., Tudur-Smith, C., Linney, M., Clark, M., Whittaker, E., Saatci, D., Davis, P. J., Luyt, K., Draper, E. S., Kenny, S. E., Fraser, L. K., and Viner, R. M. (2022). Which children and young people are at higher risk of severe disease and death after hospitalisation with SARS-CoV-2 infection in children and young people: A systematic review and individual patient meta-analysis. E Clinical Medicine 44, 101287. doi: 10.1016/j.eclinm.2022.101287 | Unrelated |
|  | Hatakeyama, K., and Seposo, X. (2022). Heatstroke-related ambulance dispatch risk before and during COVID-19 pandemic: Subgroup analysis by age, severity, and incident place. Science of the Total Environment 821, 153310. doi: 10.1016/j.scitotenv.2022.153310 | Unrelated |
|  | Hsu, C. H., Huang, H. T., Chen, C. H., Fu, Y. C., Chou, P. H., and Hsu, N. C. (2022). Review Global Impact of the COVID-19 Pandemic on Orthopedics and the Implications of Telemedicine: A Systematic Review of the Literature. Journal of Clinical Medicine 11, 2983. doi: 10.3390/jcm11112983 | Unrelated |
|  | Jacques, W., Booth, C., Wielgoszewska, B., Green, M. J., Di Gessa, G., Huggins, C. F., Griffith, G. J., Kwong, A. S. F., Bowyer, R. C. E., Maddock, J., Patalay, P., Silverwood, R. J., Fitzsimons, E., Shaw, R., Thompson, E. J., Steptoe, A., Hughes, A., Chaturvedi, N., Steves, C. J., Katikireddi, S. V., and Ploubidis, G. B. (2022). Mental and social wellbeing and the UK coronavirus job retention scheme: Evidence from nine longitudinal studies. Social Science and Medicine 308, 115226. doi: 10.1016/j.socscimed.2022.115226 | Unrelated |
|  | Jahrami, H. A., Alhaj, O. A., Humood, A. M., Alenezi, A. F., Fekih-Romdhane, F., AlRasheed, M. M., Saif, Z. Q., Bragazzi, N. L., Pandi-Perumal, S. R., BaHammam, A. S., and Vitiello, M. V. (2022). Sleep disturbances during the COVID-19 pandemic: A systematic review, meta-analysis, and meta-regression. Sleep Medicine Reviews 62, 101591. doi: 10.1016/j.smrv.2022.101591 | Unrelated |
|  | Jenabi, E., Masoumi, S. Z., Refaei, M., Ladoni, N., and Shafiei, S. (2022). The Association between Maternal COVID-19 and the Rates of Cesarean: A Systematic Review. Current Women's Health Reviews 18, e271021197411. doi: 10.2174/1573404817666211027093445 | Unrelated |
|  | Jimenez, W. P., Katz, I. M., and Liguori, E. A. (2022). Fear and Trembling While Working in a Pandemic: an Exploratory Meta-Analysis of Workers' COVID-19 Distress. Occupational health science, 1-31. doi: 10.1007/s41542-022-00131-x | Unrelated |
|  | Kannapadi, N. V., Jami, M., Premraj, L., Etchill, E. W., Giuliano, K., Bush, E. L., Kim, B. S., Seal, S., Whitman, G., and Cho, S.-M. (2022). Neurological Complications in COVID-19 Patients With ECMO Support: A Systematic Review and Meta-Analysis. Heart, lung & circulation 31, 292-298. doi: 10.1016/j.hlc.2021.10.007 | Unrelated |
|  | Karacam, Z., Kizilca-Cakaloz, D., Gunes-Ozturk, G., and Coban, A. (2022). Maternal and perinatal outcomes of pregnancy associated with COVID-19: Systematic review and meta-analysis. European journal of midwifery 6, 42. doi: 10.18332/ejm/149485 | Unrelated |
|  | Karlstad, O., Hovi, P., Husby, A., Harkanen, T., Selmer, R. M., Pihlstrom, N., Hansen Jo, V., Nohynek, H., Gunnes, N., Sundstrom, A., Wohlfahrt, J., Nieminen, T. A., Grunewald, M., Gulseth, H. L., Hviid, A., and Ljung, R. (2022). SARS-CoV-2 Vaccination and Myocarditis in a Nordic Cohort Study of 23 Million Residents. JAMA Cardiology 7, 600-612. doi: 10.1001/jamacardio.2022.0583 | Unrelated |
|  | Kasraeian, M., Zare, M., Vafaei, H., Asadi, N., Faraji, A., Bazrafshan, K., and Roozmeh, S. (2022). COVID-19 pneumonia and pregnancy; a systematic review and meta-analysis. Journal of Maternal-Fetal and Neonatal Medicine 35, 1652-1659. doi: 10.1080/14767058.2020.1763952 | Unrelated |
|  | Kazeminia, M., Afshar, Z. M., Rajati, M., Saeedi, A., and Rajati, F. (2022). Evaluation of the Acceptance Rate of Covid-19 Vaccine and its Associated Factors: A Systematic Review and Meta-analysis. Journal of prevention (2022) 43, 421-467. doi: 10.1007/s10935-022-00684-1 | Unrelated |
|  | Kuhne, L., Brussermann, S., De Santis, K. K., Jackle, S., Grimm, S., Ha, T. H., and Zeeb, H. (2022). EsteR - Decision support for German health departments by risk modelling in order to contain the COVID-19 pandemic. A rapid living-review. Gesundheitswesen, Supplement 84, 812-813. doi: 10.1055/s-0042-1753829 | Unrelated |
|  | Lai, H., Yang, M., Sun, M., Pan, B., Wang, Q., Wang, J., Tian, J., Ding, G., Yang, K., Song, X., and Ge, L. (2022). Risk of incident diabetes after COVID-19 infection: A systematic review and meta-analysis. Metabolism: Clinical and Experimental 137, 155330. doi: 10.1016/j.metabol.2022.155330 | Unrelated |
|  | Leazer, S., Collen, J., Alcover, K., Tompkins, E., Ambardar, S., Allard, R. J., Foster, B., McNutt, R., Leon, M., Haynes, Z., Bascome, M., Williams, M., Bunin, J., O'Malley, P. G., Moores, L. K., and Chung, K. K. (2022). Outcomes Associated With Intensive Care and Organ Support Among Patients With COVID-19: A Systematic Review and Meta-Analysis. Military medicine. doi: 10.1093/milmed/usac143 | Unrelated |
|  | Lee, B., Lewis, G., Agyei-Manu, E., Atkins, N., Bhattacharyya, U., Dozier, M., Rostron, J., Sheikh, A., McQuillan, R., and Theodoratou, E. (2022). Risk of serious COVID-19 outcomes among adults and children with moderate-to-severe asthma: a systematic review and meta-analysis. European Respiratory Review 31, 220066. doi: 10.1183/16000617.0066-2022 | Unrelated |
|  | Lee, J., Lee, M. Y., Jung, E., Lee, J. N., Bae, S., Jung, J., Kim, M. J., Chong, Y. P., Lee, S. O., Choi, S. H., Kim, Y. S., and Kim, S. H. (2022). Clinical Characteristics and Vertical Transmission of Pregnant Women with SARS-CoV-2 Infection and Their Neonates. Open Forum Infectious Diseases 9, S459. doi: 10.1093/ofid/ofac492.923 | Unrelated |
|  | Lee, T. C., Murthy, S., Del Corpo, O., Senecal, J., Butler-Laporte, G., Sohani, Z. N., Brophy, J. M., and McDonald, E. G. (2022). Remdesivir for the treatment of COVID-19: a systematic review and meta-analysis. Clinical Microbiology and Infection 28, 1203-1210. doi: 10.1016/j.cmi.2022.04.018 | Unrelated |
|  | Lewis, L., Sinha, I., and Losty, P. D. (2022). Respiratory syncytial virus bronchiolitis in congenital diaphragmatic hernia: A systematic review of prevalence rates and palivizumab prophylaxis. Pediatric Pulmonology 57, 239-244. doi: 10.1002/ppul.25717 | Unrelated |
|  | Li, H., Yuan, K., Sun, Y. K., Zheng, Y. B., Xu, Y. Y., Su, S. Z., Zhang, Y. X., Zhong, Y., Wang, Y. J., Tian, S. S., Gong, Y. M., Fan, T. T., Lin, X., Gobat, N., Wong, S. Y. S., Chan, E. Y. Y., Yan, W., Sun, S. W., Ran, M. S., Bao, Y. P., Shi, J., and Lu, L. (2022). Efficacy and practice of facemask use in general population: a systematic review and meta-analysis. Translational Psychiatry 12, 49. doi: 10.1038/s41398-022-01814-3 | Unrelated |
|  | Li, J., Luo, J., Pavlov, I., Perez, Y., Tan, W., Roca, O., Tavernier, E., Kharat, A., McNicholas, B., Ibarra-Estrada, M., Vines, D. L., Bosch, N. A., Rampon, G., Simpson, S. Q., Walkey, A. J., Fralick, M., Verma, A., Razak, F., Harris, T., Laffey, J. G., Guerin, C., and Ehrmann, S. (2022). Awake prone positioning for non-intubated patients with COVID-19-related acute hypoxaemic respiratory failure: a systematic review and meta-analysis. The Lancet. Respiratory medicine 10, 573-583. doi: 10.1016/S2213-2600%2822%2900043-1 | Unrelated |
|  | Liu, K., Yu, J., and Song, G. (2022). Global Myocardial Strain in Multisystem Inflammatory Syndrome in Children, Kawasaki Disease, and Healthy Children: A Network Meta-Analysis. Frontiers in Pediatrics 10, 848306. doi: 10.3389/fped.2022.848306 | Unrelated |
|  | Llinas-Caballero, K., Rodriguez, Y., Fernandez-Sarmiento, J., Rodriguez-Jimenez, M., and Anaya, J. M. (2022). Kawasaki disease in Colombia: A systematic review and contrast with multisystem inflammatory syndrome in children associated with COVID-19. Revista Colombiana de Reumatologia 29, S66-S76. doi: 10.1016/j.rcreu.2020.11.004 | Unrelated |
|  | Ma, Y., Ren, J., Zheng, Y., Cai, D., Li, S., and Li, Y. (2022). Chinese parents' willingness to vaccinate their children against COVID-19: A systematic review and meta-analysis. Frontiers in public health 10, 1087295. doi: 10.3389/fpubh.2022.1087295 | Unrelated |
|  | Magnus, M. C., Ortqvist, A. K., Dahlqwist, E., Ljung, R., Skar, F., Oakley, L., Macsali, F., Pasternak, B., Gjessing, H. K., Haberg, S. E., and Stephansson, O. (2022). Association of SARS-CoV-2 Vaccination During Pregnancy With Pregnancy Outcomes. JAMA 327, 1469-1477. doi: 10.1001/jama.2022.3271 | Unrelated |
|  | Manning, M., Khalil, H., Gupta, A., Littmoden, M., Banjoko, A., Ansari, K., Fraser, H., Ramkumar, A., Kew, T., Zhou, D., Coomar, D., Stallings, E., Allotey, J., Bonet, M., Zamora, J., Fernandez-Garcia, S., Thangaratinam, S., Yap, M., Sheikh, J., and Lawson, H. (2022). COVID-19 maternal and perinatal outcomes: living systematic review and meta-analysis (Update). BJOG: An International Journal of Obstetrics and Gynaecology 129, 144-145. doi: 10.1111/1471-0528.18_17178 | Unrelated |
|  | Marcec, R., Dodig, V. M., Radanovic, I., and Likic, R. (2022). Intravenous immunoglobulin (IVIg) therapy in hospitalised adult COVID-19 patients: A systematic review and meta-analysis. Reviews in medical virology 32, e2397. doi: 10.1002/rmv.2397 | Unrelated |
|  | Marino, S., Pavone, P., Marino, L., Nunnari, G., Ceccarelli, M., Coppola, C., Distefano, C., and Falsaperla, R. (2022). SARS-CoV-2: The Impact of Co-Infections with Particular Reference to Mycoplasma pneumonia-A Clinical Review. Microorganisms 10. doi: 10.3390/microorganisms10101936 | Unrelated |
|  | McKenzie, L., Shoukat, A., Wong, K. O., Itahashi, K., Yasuda, E., Demarsh, A., and Khan, K. (2022). Inferring the true number of SARS-CoV-2 infections in Japan. Journal of Infection and Chemotherapy 28, 1519-1522. doi: 10.1016/j.jiac.2022.08.002 | Unrelated |
|  | McQuaid, C. F., Henrion, M. Y. R., Burke, R. M., MacPherson, P., Nzawa-Soko, R., and Horton, K. C. (2022). Inequalities in the impact of COVID-19-associated disruptions on tuberculosis diagnosis by age and sex in 45 high TB burden countries. BMC Medicine 20, 432. doi: 10.1186/s12916-022-02624-6 | Unrelated |
|  | McQuire, C., Zuccolo, L., Frennesson, F., Butcher, S., Carel, H., Cook, P., Dvorak, T., Gilham, E., Hurt, L., Langford, R., Misell, A., Mukherjee, R., Porter, A., Susoy, O., Taylor-Robinson, D., Troy, D., and de Vocht, F. (2022). Prenatal alcohol prevention in the UK: mapping the landscape through systematic collaborative review. Lancet (London, England) 400, S64. doi: 10.1016/S0140-6736%2822%2902274-7 | Unrelated |
|  | Meng, S. Q., Cheng, J. L., Li, Y. Y., Yang, X. Q., Zheng, J. W., Chang, X. W., Shi, Y., Chen, Y., Lu, L., Sun, Y., Bao, Y. P., and Shi, J. (2022). Global prevalence of digital addiction in general population: A systematic review and meta-analysis. Clinical Psychology Review 92, 102128. doi: 10.1016/j.cpr.2022.102128 | Unrelated |
|  | Miao, M., Xu, Y., Yang, Y., Li, P., Jia, M., Wen, Z., Yu, M., Zhang, J., and Gu, J. (2022). Post-COVID pain and quality of life in COVID-19 patients: protocol for a meta-analysis and systematic review. BMJ Open 12, e057394. doi: 10.1136/bmjopen-2021-057394 | Unrelated |
|  | Moradi, G., Baradaran, H. R., Soheili, M., Mokhtari, M. M., and Moradi, Y. (2022). Clinical manifestation and maternal complications and neonatal outcomes in pregnant women with COVID-19: a comprehensive evidence synthesis and meta-analysis. Journal of Maternal-Fetal and Neonatal Medicine 35, 5672-5685. doi: 10.1080/14767058.2021.1888923 | Unrelated |
|  | Mose, A., Zewdie, A., and Sahle, T. (2022). Pregnant women's knowledge, attitude, and practice towards COVID-19 infection prevention in Ethiopia: A systematic review and meta-analysis. PLoS ONE 17, e0276692. doi: 10.1371/journal.pone.0276692 | Unrelated |
|  | Ng, T. K. Y., Kwok, C. K. C., Ngan, G. Y. K., Wong, H. K. H., Zoubi, F. A., Tomkins-Lane, C. C., Yau, S. K., Samartzis, D., Pinto, S. M., Fu, S. N., Li, H., and Wong, A. Y. L. (2022). Differential Effects of the COVID-19 Pandemic on Physical Activity Involvements and Exercise Habits in People With and Without Chronic Diseases: A Systematic Review and Meta-analysis. Archives of Physical Medicine and Rehabilitation 103, 1448-1465.e6. doi: 10.1016/j.apmr.2022.03.011 | Unrelated |
|  | Ng'andu, M., Mesic, A., Pry, J., Mwamba, C., Roff, F., Chipungu, J., Azgad, Y., and Sharma, A. (2022). Sexual and reproductive health services during outbreaks, epidemics, and pandemics in sub-Saharan Africa: a literature scoping review. Systematic Reviews 11, 161. doi: 10.1186/s13643-022-02035-x | Unrelated |
|  | Nguyen, P., Ananthapavan, J., Tan, E. J., Crosland, P., Bowe, S. J., Gao, L., Dunstan, D. W., and Moodie, M. (2022). Modelling the potential health and economic benefits of reducing population sitting time in Australia. The international journal of behavioral nutrition and physical activity 19, 28. doi: 10.1186/s12966-022-01276-2 | Unrelated |
|  | Nijakowski, K., Wyzga, S., Singh, N., Podgorski, F., and Surdacka, A. (2022). Oral Manifestations in SARS-CoV-2 Positive Patients: A Systematic Review. Journal of Clinical Medicine 11, 2202. doi: 10.1186/s12966-022-01276-2 | Unrelated |
|  | Oh, C., Carducci, B., Vaivada, T., and Bhutta, Z. A. (2022). Interventions to Promote Physical Activity and Healthy Digital Media Use in Children and Adolescents: A Systematic Review. Pediatrics 149. doi: 10.1542/peds.2021-053852I | Unrelated |
|  | Okita, Y., Morita, T., and Kumanogoh, A. (2022). Duration of SARS-CoV-2 RNA positivity from various specimens and clinical characteristics in patients with COVID-19: a systematic review and meta-analysis. Inflammation and Regeneration 42, 16. doi: 10.1186/s41232-022-00205-x | Unrelated |
|  | Omar, M., Youssef, M. R., Trinh, L. N., Attia, A. S., Elshazli, R. M., Jardak, C. L., Farhoud, A. S., Hussein, M. H., Shihabi, A., Elnahla, A., Zora, G., Abdelgawad, M., Munshi, R., Aboueisha, M., Toraih, E. A., Fawzy, M. S., and Kandil, E. (2022). Excess of cesarean births in pregnant women with COVID-19: A meta-analysis. Birth (Berkeley, Calif.) 49, 179-193. doi: 10.1111/birt.12609 | Unrelated |
|  | Otiende, M. (2022). All-cause and cause-specific mortality in Kilifi Health and Demographic Surveillance System, in Kenya in the period of the COVID-19 pandemic. Journal of Public Health in Africa 13, 9-10. | Unrelated |
|  | Pallavicini, F., Pepe, A., and Mantovani, F. (2022). The Effects of Playing Video Games on Stress, Anxiety, Depression, Loneliness, and Gaming Disorder During the Early Stages of the COVID-19 Pandemic: PRISMA Systematic Review. Cyberpsychology, behavior and social networking 25, 334-354. doi: 10.1089/cyber.2021.0252 | Unrelated |
|  | Pastorino, R., Pezzullo, A. M., Villani, L., Causio, F. A., Axfors, C., Contopoulos-Ioannidis, D. G., Boccia, S., and Ioannidis, J. P. A. (2022). Change in age distribution of COVID-19 deaths with the introduction of COVID-19 vaccination. Environmental research 204, 112342. doi: 10.1016/j.envres.2021.112342 | Unrelated |
|  | Patoulias, D., Katsimardou, A., Stavropoulos, K., Imprialos, K., Aidinis, A., Sviggou, A., Kordalis, V., Pyrpasopoulou, A., Papadopoulos, C., and Doumas, M. (2022). COVID-19 IS ASSOCIATED WITH ENDOTHELIAL DYSFUNCTION: A META-ANALYSIS OF OBSERVATIONAL STUDIES. Journal of Hypertension 40, e169. doi: 10.1097/01.hjh.0000837084.91831.2a | Unrelated |
|  | Patra, P. K., Das, R. R., Banday, A. Z., Singh, M., Goyal, K., Jindal, A. K., and Singh, S. (2022). Non-SARS, non-MERS human coronavirus infections and risk of Kawasaki disease: A meta-analysis. Future Virology 17, 37-47. doi: 10.2217/fvl-2021-0176 | Unrelated |
|  | Pecoraro, V., Negro, A., Pirotti, T., and Trenti, T. (2022). Estimate false-negative RT-PCR rates for SARS-CoV-2. A systematic review and meta-analysis. European Journal of Clinical Investigation 52, e13706. doi: 10.1111/eci.13706 | Unrelated |
|  | Peiris, S., Ordunez, P., DiPette, D., Padwal, R., Ambrosi, P., Toledo, J., Stanford, V., Lisboa, T., Aldighieri, S., and Reveiz, L. (2022). Cardiac Manifestations in Patients with COVID-19: A Scoping Review. Global Heart 17, 2. doi: 10.5334/GH.1037 | Unrelated |
|  | Perez-Lopez, F. R., Saviron-Cornudella, R., Chedraui, P., Lopez-Baena, M. T., Perez-Roncero, G., Sanz-Arenal, A., Narvaez-Salazar, M., Dieste-Perez, P., and Tajada, M. (2022). Obstetric and perinatal outcomes of pregnancies with COVID 19: a systematic review and meta-analysis. Journal of Maternal-Fetal and Neonatal Medicine 35, 9742-9758. doi: 10.1080/14767058.2022.2051008 | Unrelated |
|  | Pham, B., Rios, P., Radhakrishnan, A., Darvesh, N., Antony, J., Williams, C., Ramkissoon, N., Cormack, G. V., Grossman, M. R., Kampman, M., Patel, M., Yazdi, F., Robson, R., Ghassemi, M., MacDonald, E., Warren, R., Muller, M. P., Straus, S. E., and Tricco, A. C. (2022). Comparative-effectiveness research of COVID-19 treatment: a rapid scoping review. BMJ Open 12, e045115. doi: 10.1136/bmjopen-2020-045115 | Unrelated |
|  | Pillay, J., Rahman, S., Guitard, S., Wingert, A., and Hartling, L. (2022). Risk factors and preventive interventions for post Covid-19 condition: systematic reviews. medRxiv. doi: 10.1101/2022.03.25.22272949 | Unrelated |
|  | Pischel, L., Patel, K. M., Goshua, G., and Omer, S. B. (2022). Adenovirus-Based Vaccines and Thrombosis in Pregnancy: A Systematic Review and Meta-analysis. Clinical infectious diseases: an official publication of the Infectious Diseases Society of America 75, 1179-1186. doi: 10.1093/cid/ciac080 | Unrelated |
|  | Pizzol, D., Shin, J. I., Trott, M., Ilie, P. C., Ippoliti, S., Carrie, A. M., Ghayda, R. A., Lozano, J. M. O., Muyor, J. M., Butler, L., McDermott, D. T., Barnett, Y., Markovic, L., Grabovac, I., Koyanagi, A., Soysal, P., Tully, M. A., Veronese, N., and Smith, L. (2022). Social environmental impact of COVID-19 and erectile dysfunction: an explorative review. Journal of endocrinological investigation 45, 483-487. doi: 10.1007/s40618-021-01679-1 | Unrelated |
|  | Prasad, C., Varalakshmi, S., and Mukkadan, J. K. (2022). Impact of covid 19 on parturition and neonates in India by assessing selected maternal factors and neonatal parameters - A meta-analysis and systematic review. NeuroQuantology 20, 1620-1628. doi: 10.14704/NQ.2022.20.15.NQ88148 | Unrelated |
|  | Prasad, S., Kalafat, E., Blakeway, H., Townsend, R., O'Brien, P., Morris, E., Draycott, T., Thangaratinam, S., Le Doare, K., Ladhani, S., Von Dadelszen, P., Magee, L. A., Heath, P., and Khalil, A. (2022). Effectiveness and perinatal outcomes of COVID-19 vaccination in pregnancy: SystematicReview and meta-analysis. BJOG: An International Journal of Obstetrics and Gynaecology 129, 144. doi: 10.1111/1471-0528.18_17178 | Unrelated |
|  | Prasad, S., Kalafat, E., Blakeway, H., Townsend, R., O'Brien, P., Morris, E., Draycott, T., Thangaratinam, S., Le Doare, K., Ladhani, S., von Dadelszen, P., Magee, L. A., Heath, P., and Khalil, A. (2022). Systematic review and meta-analysis of the effectiveness and perinatal outcomes of COVID-19 vaccination in pregnancy. Nature communications 13, 2414. doi: 10.1038/s41467-022-30052-w | Unrelated |
|  | Quintas, J., Guerreiro, A., de Carvalho, M. J. L., Duarte, V., Pedro, A. R., Gama, A. F., Keygnaert, I., and Dias, S. (2022). The Implication of the First Wave of COVID-19 on Mental Health: Results from a Portuguese Sample. International journal of environmental research and public health 19. doi: 10.3390/ijerph19116489 | Unrelated |
|  | Ramirez-Soto, M. C., Ortega-Caceres, G., and Arroyo-Hernandez, H. (2022). Excess all-cause deaths stratified by sex and age in Peru: A time series analysis during the COVID-19 pandemic. BMJ Open 12, e057056. doi: 10.1136/bmjopen-2021-057056 | Unrelated |
|  | Ravindra, K., Malik, V. S., Padhi, B. K., Goel, S., and Gupta, M. (2022). Asymptomatic infection and transmission of COVID-19 among clusters: systematic review and meta-analysis. Public Health 203, 100-109. doi: 10.1016/j.puhe.2021.12.003 | Unrelated |
|  | Rawal, S., Tackett, R. L., Stone, R. H., and Young, H. N. (2022). EPH103 COVID-19 Vaccination Among Pregnant Women in the US: A Systematic Review. Value in Health 25, S453-S454. doi: 10.1016/j.jval.2022.04.855 | Unrelated |
|  | Robinson, E., Sutin, A. R., Daly, M., and Jones, A. (2022). A systematic review and meta-analysis of longitudinal cohort studies comparing mental health before versus during the COVID-19 pandemic in 2020. Journal of Affective Disorders 296, 567-576. doi: 10.1016/j.jad.2021.09.098 | Unrelated |
|  | Rusconi, F., Puglia, M., Pacifici, M., Brescianini, S., Gagliardi, L., Nannavecchia, A. M., Buono, P., Cantoira, S., Farchi, S., Gobbato, M., Pellegrini, E., Perrone, E., Pertile, R., Tavormina, E. E., Visona Dalla Pozza, L., and Zambri, F. (2022). Pregnancy outcomes in Italy during COVID-19 pandemic: a population-based cohort study. BJOG: an international journal of obstetrics and gynaecology. doi: 10.1111/1471-0528.17315 | Unrelated |
|  | Sabeena, S., Ravishankar, N., Robin, S., and Pillai, S. S. (2022). The impact of COVID-19 pandemic on bronchiolitis (lower respiratory tract infection) due to respiratory syncytial virus: A systematic review and meta-analysis. medRxiv. doi: 10.1101/2022.04.26.22274244 | Unrelated |
|  | Sadeghi, S., Kalantari, Y., Shokri, S., Fallahpour, M., Nafissi, N., Goodarzi, A., and Valizadeh, R. (2022). Immunologic response, Efficacy, and Safety of Vaccines against COVID-19 Infection in Healthy and immunosuppressed Children and Adolescents Aged 2 - 21 years old: A Systematic Review and Meta-analysis. Journal of Clinical Virology 153, 105196. doi: 10.1016/j.jcv.2022.105196 | Unrelated |
|  | Saha, S. (2022). Death and invasive mechanical ventilation risk in hospitalized COVID-19 patients treated with anti-SARS-CoV-2 monoclonal antibodies and/or antiviral agents: A systematic review and network meta-analysis protocol. PloS one 17, e0270196. doi: 10.1371/journal.pone.0270196 | Unrelated |
|  | Sakuraba, A., Luna, A., and Micic, D. (2022). Serologic Response to Coronavirus Disease 2019 (COVID-19) Vaccination in Patients With Immune-Mediated Inflammatory Diseases: A Systematic Review and Meta-analysis. Gastroenterology 162, 88-108.e9. doi: 10.1053/j.gastro.2021.09.055 | Unrelated |
|  | Santos, J. N. V., Mendonca, V. A., Fernandes, A. C., Maia, L. B., Henschke, N., de Souza, M. B., da Silva Lage, V. K., Oliveira, M. X., de Fatima Silva, A., Rodrigues Lacerda, A. C., Sartorio, A., Rapin, A., de Oliveira, V. C., and Taiar, R. (2022). Recent Advance Analysis of Recovery in Hospitalized People with COVID-19: A Systematic Review. International journal of environmental research and public health 19. doi: 10.3390/ijerph192114609 | Unrelated |
|  | Santos, M. O., Goncalves, L. C., Silva, P. A. N., Moreira, A. L. E., Ito, C. R. M., Peixoto, F. A. O., Wastowski, I. J., Carneiro, L. C., and Avelino, M. A. G. (2022). Multisystem inflammatory syndrome (MIS-C): a systematic review and meta-analysis of clinical characteristics, treatment, and outcomes. Jornal de Pediatria 98, 338-349. doi: 10.1016/j.jped.2021.08.006 | Unrelated |
|  | Saville, O., Tera, Y., Deng, Y., Elbatarny, M., and Othman, M. (2022). The impact of sex on D-Dimer levels and disease outcomes in hospitalized COVID-19 patients. Research and Practice in Thrombosis and Haemostasis 6. doi: 10.1002/rth2.12788 | Unrelated |
|  | Shafiee, A., Teymouri Athar, M. M., Nassar, M., Seighali, N., Aminzade, D., Fattahi, P., Rahmannia, M., and Ahmadi, Z. (2022). Comparison of COVID-19 outcomes in patients with Type 1 and Type 2 diabetes: A systematic review and meta-analysis. Diabetes and Metabolic Syndrome: Clinical Research and Reviews 16, 102512. doi: 10.1016/j.dsx.2022.102512 | Unrelated |
|  | Shahzad, M., Chaudhary, S. G., Zafar, M. U., Hassan, M. A., Hussain, A., Ali, F., Anwar, I., Ahmed, M., Ahmed, N., Khurana, S., Rauf, M. A., Anwar, F., Hematti, P., Callander, N. S., Abhyankar, S. H., McGuirk, J. P., and Mushtaq, M. U. (2022). Impact of COVID-19 in hematopoietic stem cell transplant recipients: A systematic review and meta-analysis. Transplant Infectious Disease 24, e13792. doi: 10.1111/tid.13792 | Unrelated |
|  | Shaikh, C. F., Palmer Kelly, E., Paro, A., Cloyd, J., Ejaz, A., Beal, E. W., and Pawlik, T. M. (2022). Burnout Assessment Among Surgeons and Surgical Trainees During the COVID-19 Pandemic: A Systematic Review. Journal of Surgical Education 79, 1206-1220. doi: 10.1016/j.jsurg.2022.04.015 | Unrelated |
|  | Sheikh, J., Lawson, H., Allotey, J., Yap, M., Balaji, R., Kew, T., Stallings, E., Coomar, D., Gaetano-Gil, A., Zamora, J., and Thangaratinam, S. (2022). Global variations in the burden of SARS-CoV-2 infection and its outcomes in pregnant women by geographical region and country's income status: a meta-analysis. BMJ global health 7. doi: 10.1136/bmjgh-2022-010060 | Unrelated |
|  | Shi, N., Huang, J., Ai, J., Wang, Q., Cui, T., Yang, L., Ji, H., Bao, C., and Jin, H. (2022). Transmissibility and pathogenicity of the severe acute respiratory syndrome coronavirus 2: A systematic review and meta-analysis of secondary attack rate and asymptomatic infection. Journal of Infection and Public Health 15, 297-306. doi: 10.1016/j.jiph.2022.01.015 | Unrelated |
|  | Shi, S., Wang, F., Yao, H., Kou, S., Li, W., Chen, B., Wu, Y., Wang, X., Pei, C., Huang, D., Wang, Y., Zhang, P., He, Y., and Wang, Z. (2022). Oral Chinese Herbal Medicine on Immune Responses During Coronavirus Disease 2019: A Systematic Review and Meta-Analysis. Frontiers in Medicine 8, 685734. doi: 10.3389/fmed.2021.685734 | Unrelated |
|  | Spencer, N., Markham, W., Johnson, S., Arpin, E., Nathawad, R., Gunnlaugsson, G., Homaira, N., Rubio, M. L. M., and Trujillo, C. J. (2022). The Impact of COVID-19 Pandemic on Inequity in Routine Childhood Vaccination Coverage: A Systematic Review. Vaccines 10, 1013. doi: 10.3390/vaccines10071013 | Unrelated |
|  | Ssentongo, P., Ssentongo, A. E., Voleti, N., Groff, D., Sun, A., Ba, D. M., Nunez, J., Parent, L. J., Chinchilli, V. M., and Paules, C. I. (2022). SARS-CoV-2 vaccine effectiveness against infection, symptomatic and severe COVID-19: a systematic review and meta-analysis. BMC infectious diseases 22, 439. doi: 10.1186/s12879-022-07418-y | Unrelated |
|  | Struyf, T., Deeks, J. J., Dinnes, J., Takwoingi, Y., Davenport, C., Leeflang, M. M. G., Spijker, R., Hooft, L., Emperador, D., Domen, J., Tans, A., Janssens, S., Wickramasinghe, D., Lannoy, V., Horn, S. R. A., and Van den Bruel, A. (2022). Signs and symptoms to determine if a patient presenting in primary care or hospital outpatient settings has COVID-19. Cochrane Database of Systematic Reviews 2022, CD013665. doi: 10.1002/14651858.CD013665.pub3 | Unrelated |
|  | Subramaniam, A., Shekar, K., Afroz, A., Ashwin, S., Billah, B., Brown, H., Kundi, H., Lim, Z. J., Ponnapa Reddy, M., and Curtis, J. R. (2022). Frailty and mortality associations in patients with COVID-19: a systematic review and meta-analysis. Internal medicine journal 52, 724-739. doi: 10.1111/imj.15698 | Unrelated |
|  | Sunderraj, A., Otero, S., Miller, E., Shanes, E. D., Goldstein, J. A., and Mithal, L. B. (2022). Cord blood and maternal cytokine levels following SARS-CoV-2 infection during pregnancy. Open Forum Infectious Diseases 9, S927-S928. doi: 10.1093/ofid/ofac492.1880 | Unrelated |
|  | Sutton, N., San Francisco Ramos, A., Beales, E., Smith, D., Ikram, S., Galiza, E., Hsia, Y., and Heath, P. T. (2022). Comparing reactogenicity of COVID-19 vaccines: a systematic review and meta-analysis. Expert Review of Vaccines 21, 1301-1318. doi: 10.1080/14760584.2022.2098719 | Unrelated |
|  | Swarnakar, R., Jenifa, S., and Wadhwa, S. (2022). Musculoskeletal complications in long COVID-19: A systematic review. World journal of virology 11, 485-495. doi: 10.5501/wjv.v11.i6.485 | Unrelated |
|  | Tan, C. J.-W., Tan, B. K. J., Tan, X. Y., Liu, H. T., Teo, C. B., See, A., Xu, S., Toh, S. T., Kheok, S. W., Charn, T. C., and Teo, N. W. Y. (2022). Neuroradiological Basis of COVID-19 Olfactory Dysfunction: A Systematic Review and Meta-Analysis. The Laryngoscope 132, 1260-1274. doi: 10.1002/lary.30078 | Unrelated |
|  | Tan, R. S. J., Ng, K. T., Xin, C. E., Atan, R., Yunos, N. a. M., and Hasan, M. S. (2022). High-Dose versus Low-Dose Corticosteroids in COVID-19 Patients: a Systematic Review and Meta-analysis. Journal of cardiothoracic and vascular anesthesia 36, 3576-3586. doi: 10.1053/j.jvca.2022.05.011 | Unrelated |
|  | Tang, H. J., Weng, T. S., Chen, Y. H., and Chao, C. M. (2022). Oral Janus kinase inhibitors for treating hospitalized patients with COVID-19: An updated systematic review and meta-analysis of randomized controlled trials. Journal of Microbiology, Immunology and Infection 55, 1025-1035. doi: 10.1016/j.jmii.2022.08.005 | Unrelated |
|  | Teismann, T., Lewitzka, U., and Brieger, P. (2022). Suicidal ideation and behavior during COVID-19 pandemic. Nervenheilkunde 41, 215-221. doi: 10.1055/a-1755-1648 | Unrelated |
|  | Trott, M., Driscoll, R., Irlado, E., and Pardhan, S. (2022). Changes and correlates of screen time in adults and children during the COVID-19 pandemic: A systematic review and meta-analysis. eClinicalMedicine 48, 101452. doi: 10.1016/j.eclinm.2022.101452 | Unrelated |
|  | Van Baar, J., Kostova, E., and Van Wely, M. (2022). COVID-19 in pregnant women: a living systematic review and meta-analysis on the risk and prevalence of pregnancy loss. Human Reproduction 37, i80. doi: 10.1093/humrep/deac106.O-139 | Unrelated |
|  | Vaseghi, N., Sharifisooraki, J., Khodadadi, H., Nami, S., Safari, F., Ahangarkani, F., Meis, J. F., Badali, H., and Morovati, H. (2022). Global prevalence and subgroup analyses of coronavirus disease (COVID -19) associated Candida auris infections (CACa): A systematic review and meta-analysis. Mycoses 65, 683-703. doi: 10.1111/myc.13471 | Unrelated |
|  | Vlieg-Boerstra, B., de Jong, N., Meyer, R., Agostoni, C., De Cosmi, V., Grimshaw, K., Milani, G. P., Muraro, A., Oude Elberink, H., Pali-Scholl, I., Roduit, C., Sasaki, M., Skypala, I., Sokolowska, M., van Splunter, M., Untersmayr, E., Venter, C., O'Mahony, L., and Nwaru, B. I. (2022). Nutrient supplementation for prevention of viral respiratory tract infections in healthy subjects: A systematic review and meta-analysis. Allergy 77, 1373-1388. doi: 10.1111/all.15136 | Unrelated |
|  | Vos, T., Hanson, S. W., Abbafati, C., Aerts, J. G., Al-Aly, Z., et al. (2022). Estimated Global Proportions of Individuals with Persistent Fatigue, Cognitive, and Respiratory Symptom Clusters Following Symptomatic COVID-19 in 2020 and 2021. JAMA 328, 1604-1615. doi: 10.1001/jama.2022.18931 | Unrelated |
|  | Wafa, I. A., Pratama, N. R., Sofia, N. F., Anastasia, E. S., Konstantin, T., Wijaya, M. A., Rifqi Wiyono, M., Djuari, L., and Novida, H. (2022). Impact of COVID-19 Lockdown on the Metabolic Control Parameters in Patients with Diabetes Mellitus: A Systematic Review and Meta-Analysis. Diabetes and Metabolism Journal 46, 260-272. doi: 10.4093/dmj.2021.0125 | Unrelated |
|  | Wang, B., Andraweera, P., Elliott, S., Mohammed, H., Lassi, Z., Twigger, A., Borgas, C., Gunasekera, S., Ladhani, S., and Marshall, H. S. (2022). Asymptomatic SARS-CoV-2 infection by age: A systematic review and meta-analysis. medRxiv doi: 10.1101/2022.05.05.22274697 | Unrelated |
|  | Wang, J., Geng, Y., Fang, Z., Zhu, L., Chen, Y., and Yao, Y. (2022). Meta-analysis of the prevalence of anxiety and depression among frontline healthcare workers during the COVID-19 pandemic. Frontiers in public health 10, 984630. doi: 10.3389/fpubh.2022.984630 | Unrelated |
|  | Wang, X., Chen, X., and Zhang, K. (2022). Maternal infection with COVID-19 and increased risk of adverse pregnancy outcomes: a meta-analysis. The journal of maternal-fetal & neonatal medicine: the official journal of the European Association of Perinatal Medicine, the Federation of Asia and Oceania Perinatal Societies, the International Society of Perinatal Obstetricians 35, 9368-9375. doi: 10.1080/14767058.2022.2033722 | Unrelated |
|  | Whited, N., and Cervantes, J. (2022). Antibodies Against SARS-CoV-2 in Human Breast Milk after Vaccination: A Systematic Review and Meta-Analysis. Breastfeeding Medicine 17, 475-483. doi: 10.1089/bfm.2021.0353 | Unrelated |
|  | Wielgoszewska, B., Maddock, J., Green, M. J., Di Gessa, G., Parsons, S., Griffith, G. J., Croft, J., Stevenson, A. J., Booth, C., Silverwood, R. J., Bann, D., Patalay, P., Hughes, A. D., Chaturvedi, N., Howe, L. D., Fitzsimons, E., Katikireddi, S. V., and Ploubidis, G. B. (2022). The UK Coronavirus Job Retention Scheme and diet, physical activity, and sleep during the COVID-19 pandemic: evidence from eight longitudinal population surveys. BMC medicine 20, 147. doi: 10.1186/s12916-022-02343-y | Unrelated |
|  | Wong, H.-L., Hu, M., Zhou, C. K., Lloyd, P. C., Amend, K. L., Beachler, D. C., Secora, A., McMahill-Walraven, C. N., Lu, Y., Wu, Y., Ogilvie, R. P., Reich, C., Djibo, D. A., Wan, Z., Seeger, J. D., Akhtar, S., Jiao, Y., Chillarige, Y., Do, R., Hornberger, J., Obidi, J., Forshee, R., Shoaibi, A., and Anderson, S. A. (2022). Risk of myocarditis and pericarditis after the COVID-19 mRNA vaccination in the USA: a cohort study in claims databases. Lancet (London, England) 399, 2191-2199. doi: 10.1016/S0140-6736(22)00791-7 | Unrelated |
|  | Wu, Q., Luo, S., and Xie, X. (2022). The impact of anti-tumor approaches on the outcomes of cancer patients with COVID-19: a meta-analysis based on 52 cohorts incorporating 9231 participants. BMC cancer 22, 241. doi: 10.1186/s12885-022-09320-x | Unrelated |
|  | Wu, X. X., Yao, J. J., Qian, J., Huang, Q. F., Deng, T., Xu, S. Q., Wang, H. F., Li, Q., Peng, J. C., Yi, Y., Li, N., Huang, Y., and Liu, X. R. (2022). Incidence of adverse reactions to COVID-19 vaccination: A meta-analysis of randomized controlled trials. Journal of Acute Disease 11, 1-11. doi: 10.4103/2221-6189.336575 | Unrelated |
|  | Wu, Y., Kang, L., Guo, Z., Liu, J., Liu, M., and Liang, W. (2022). Incubation Period of COVID-19 Caused by Unique SARS-CoV-2 Strains: A Systematic Review and Meta-analysis. JAMA network open 5, e2228008. doi: 10.1001/jamanetworkopen.2022.28008 | Unrelated |
|  | Wulf Hanson, S., Abbafati, C., Aerts, J. G., Al-Aly, Z., Ashbaugh, C., Ballouz, T., Blyuss, O., Bobkova, P., Bonsel, G., Borzakova, S., Buonsenso, D., Butnaru, D., Carter, A., Chu, H., De Rose, C., Diab, M. M., Ekbom, E., El Tantawi, M., Fomin, V., Frithiof, R., Gamirova, A., Glybochko, P. V., Haagsma, J. A., Javanmard, S. H., Hamilton, E. B., Harris, G., Heijenbrok-Kal, M. H., Helbok, R., Hellemons, M. E., Hillus, D., Huijts, S. M., Hultstrom, M., Jassat, W., Kurth, F., Larsson, I.-M., Lipcsey, M., Liu, C., Loflin, C. D., Malinovschi, A., Mao, W., Mazankova, L., McCulloch, D., Menges, D., Mohammadifard, N., Munblit, D., Nekliudov, N. A., Ogbuoji, O., Osmanov, I. M., Penalvo, J. L., Petersen, M. S., Puhan, M. A., Rahman, M., Rass, V., Reinig, N., Ribbers, G. M., Ricchiuto, A., Rubertsson, S., Samitova, E., Sarrafzadegan, N., Shikhaleva, A., Simpson, K. E., Sinatti, D., Soriano, J. B., Spiridonova, E., Steinbeis, F., Svistunov, A. A., Valentini, P., van de Water, B. J., van den Berg-Emons, R., Wallin, E., Witzenrath, M., Wu, Y., Xu, H., Zoller, T., Adolph, C., Albright, J., Amlag, J. O., Aravkin, A. Y., Bang-Jensen, B. L., Bisignano, C., Castellano, R., Castro, E., Chakrabarti, S., Collins, J. K., Dai, X., Daoud, F., Dapper, C., Deen, A., Duncan, B. B., Erickson, M., Ewald, S. B., Ferrari, A. J., Flaxman, A. D., Fullman, N., Gamkrelidze, A., Giles, J. R., Guo, G., Hay, S. I., He, J., Helak, M., et al. (2022). A global systematic analysis of the occurrence, severity, and recovery pattern of long COVID in 2020 and 2021. medRxiv : the preprint server for health sciences. doi: 10.1101/2022.05.26.22275532 | Unrelated |
|  | Wulf Hanson, S., Abbafati, C., Aerts, J. G., Al-Aly, Z., Ashbaugh, C., Ballouz, T., Blyuss, O., Bobkova, P., Bonsel, G., Borzakova, S., Buonsenso, D., Butnaru, D., Carter, A., Chu, H., De Rose, C., Diab, M. M., Ekbom, E., El Tantawi, M., Fomin, V., Frithiof, R., Gamirova, A., Glybochko, P. V., Haagsma, J. A., Haghjooy Javanmard, S., Hamilton, E. B., Harris, G., Heijenbrok-Kal, M. H., Helbok, R., Hellemons, M. E., Hillus, D., Huijts, S. M., Hultstrom, M., Jassat, W., Kurth, F., Larsson, I.-M., Lipcsey, M., Liu, C., Loflin, C. D., Malinovschi, A., Mao, W., Mazankova, L., McCulloch, D., Menges, D., Mohammadifard, N., Munblit, D., Nekliudov, N. A., Ogbuoji, O., Osmanov, I. M., Penalvo, J. L., Petersen, M. S., Puhan, M. A., Rahman, M., Rass, V., Reinig, N., Ribbers, G. M., Ricchiuto, A., Rubertsson, S., Samitova, E., Sarrafzadegan, N., Shikhaleva, A., Simpson, K. E., Sinatti, D., Soriano, J. B., Spiridonova, E., Steinbeis, F., Svistunov, A. A., Valentini, P., van de Water, B. J., van den Berg-Emons, R., Wallin, E., Witzenrath, M., Wu, Y., Xu, H., Zoller, T., Adolph, C., Albright, J., Amlag, J. O., Aravkin, A. Y., Bang-Jensen, B. L., Bisignano, C., Castellano, R., Castro, E., Chakrabarti, S., Collins, J. K., Dai, X., Daoud, F., Dapper, C., Deen, A., Duncan, B. B., Erickson, M., Ewald, S. B., Ferrari, A. J., Flaxman, A. D., Fullman, N., Gamkrelidze, A., Giles, J. R., Guo, G., Hay, S. I., He, J., Helak, M., et al. (2022). Estimated Global Proportions of Individuals With Persistent Fatigue, Cognitive, and Respiratory Symptom Clusters Following Symptomatic COVID-19 in 2020 and 2021. JAMA 328, 1604-1615. doi: 10.1001/jama.2022.18931 | Unrelated |
|  | Wunsch, K., Kienberger, K., and Niessner, C. (2022). Changes in Physical Activity Patterns Due to the COVID-19 Pandemic: A Systematic Review and Meta-Analysis. International Journal of Environmental Research and Public Health 19, 2250. doi: 10.3390/ijerph19042250 | Unrelated |
|  | Yang, J., D'Souza, R., Kharrat, A., Fell, D. B., Snelgrove, J. W., Murphy, K. E., and Shah, P. S. (2022). Coronavirus disease 2019 pandemic and pregnancy and neonatal outcomes in general population: A living systematic review and meta-analysis (updated Aug 14, 2021). Acta obstetricia et gynecologica Scandinavica 101, 7-24. doi: 10.1111/aogs.14277 | Unrelated |
|  | Yang, J., D'Souza, R., Kharrat, A., Fell, D. B., Snelgrove, J. W., and Shah, P. S. (2022). COVID-19 pandemic and population-level pregnancy and neonatal outcomes in general population: A living systematic review and meta-analysis (Update#2: November 20, 2021). Acta Obstetricia et Gynecologica Scandinavica 101, 273-292. doi: 10.1111/aogs.14318 | Unrelated |
|  | Yang, Z., Wang, X., Zhang, S., Ye, H., Chen, Y., and Xia, Y. (2022). Pediatric Myopia Progression During the COVID-19 Pandemic Home Quarantine and the Risk Factors: A Systematic Review and Meta-Analysis. Frontiers in public health 10, 835449. doi: 10.3389/fpubh.2022.835449 | Unrelated |
|  | Yao, X. D., Zhu, L. J., Yin, J., and Wen, J. (2022). Impacts of COVID-19 pandemic on preterm birth: a systematic review and meta-analysis. Public health 213, 127-134. doi: 10.1016/j.puhe.2022.10.015 | Unrelated |
|  | Yosep, I., Prayogo, S. A., Kohar, K., Andrew, H., Mardhiyah, A., Amirah, S., and Maulana, S. (2022). Managing Autism Spectrum Disorder in the Face of Pandemic Using Internet-Based Parent-Mediated Interventions: A Systematic Review of Randomized Controlled Trials. Children (Basel, Switzerland) 9. doi: 10.3390/children9101483 | Unrelated |
|  | Yu, W., Guo, Y., Zhang, S., Kong, Y., Shen, Z., and Zhang, J. (2022). Proportion of asymptomatic infection and nonsevere disease caused by SARS-CoV-2 Omicron variant: A systematic review and analysis. Journal of Medical Virology 94, 5790-5801. doi: 10.1002/jmv.28066 | Unrelated |
|  | Zewdie, A., Mose, A., Yimer, A., Melis, T., Muhamed, A. N., and Jemal, A. K. (2022). Essential maternal health service disruptions in Ethiopia during COVID 19 pandemic: a systematic review. BMC Women's Health 22, 496. doi: 10.1186/s12905-022-02091-4 | Unrelated |
|  | Zhang, J., Cao, J., and Ye, Q. (2022). Renal Side Effects of COVID-19 Vaccination. Vaccines 10, 1783. doi: 10.3390/vaccines10111783 | Unrelated |
|  | Zheng, C., Shao, W., Chen, X., Zhang, B., Wang, G., and Zhang, W. (2022). Real-world effectiveness of COVID-19 vaccines: a literature review and meta-analysis. International Journal of Infectious Diseases 114, 252-260. doi: 10.1016/j.ijid.2021.11.009 | Unrelated |
|  | Zhou, X., Pu, Y., Zhang, D., Xia, Y., Guan, Y., Liu, S., and Fan, L. (2022). CT findings and dynamic imaging changes of COVID-19 in 2908 patients: a systematic review and meta-analysis. Acta radiologica (Stockholm, Sweden: 1987) 63, 291-310. doi: 10.1177/0284185121992655 | Unrelated |
|  | Anderson, L. N., Yoshida-Montezuma, Y., Dewart, N., Jalil, E., Khattar, J., De Rubeis, V., Carsley, S., Griffith, L. E., and Mbuagbaw, L. (2023). Obesity and weight change during the COVID-19 pandemic in children and adults: A systematic review and meta-analysis. Obesity Reviews. doi: 10.1111/obr.13550 | Unrelated |
|  | Axfors, C., Pezzullo, A. M., Contopoulos-Ioannidis, D. G., Apostolatos, A., and Ioannidis, J. P. (2023). Differential COVID-19 infection rates in children, adults, and elderly: Systematic review and meta-analysis of 38 pre-vaccination national seroprevalence studies. Journal of global health 13, 06004. doi: 10.7189/jogh.13.06004 | Unrelated |
|  | Azevedo, F. M., de Morais, N. d. S., Silva, D. L. F., Candido, A. C., Morais, D. d. C., Priore, S. E., and Franceschini, S. d. C. C. (2023). Food insecurity and its socioeconomic and health determinants in pregnant women and mothers of children under 2 years of age, during the COVID-19 pandemic: A systematic review and meta-analysis. Frontiers in public health 11, 1087955. doi: 10.3389/fpubh.2023.1087955 | Unrelated |
|  | Bailey, A. J. M., Maganti, H. B., Cheng, W., Shorr, R., Arianne Buchan, C., and Allan, D. S. (2023). Humoral and Cellular Response of Transplant Recipients to a Third Dose of mRNA SARS-CoV-2 Vaccine: A Systematic Review and Meta-analysis. Transplantation 107, 204-215. doi: 10.1097/TP.0000000000004386 | Unrelated |
|  | Betthauser, B. A., Bach-Mortensen, A. M., and Engzell, P. (2023). A systematic review and meta-analysis of the evidence on learning during the COVID-19 pandemic. Nature human behaviour. doi: 10.1038/s41562-022-01506-4 | Unrelated |
|  | Bianchi, F. P., Stefanizzi, P., Cuscianna, E., Riformato, G., Di Lorenzo, A., Giordano, P., Germinario, C. A., and Tafuri, S. (2023). COVID-19 vaccination hesitancy among Italian parents: A systematic review and meta-analysis. Human Vaccines and Immunotherapeutics, 2171185. doi: 10.1080/21645515.2023.2171185 | Unrelated |
|  | Delanerolle, G., McCauley, M., Hirsch, M., Zeng, Y., Cong, X., Cavalini, H., Sajid, S., Shetty, A., Rathod, S., Shi, J. Q., Hapangama, D. K., and Phiri, P. (2023). The prevalence of mental ill-health in women during pregnancy and after childbirth during the Covid-19 pandemic: a systematic review and Meta-analysis. BMC Pregnancy and Childbirth 23, 76. doi: 10.1186/s12884-022-05243-4 | Unrelated |
|  | Ding, C., Liu, Y., Pang, W., Zhang, D., Wang, K., and Chen, Y. (2023). Associations of COVID-19 vaccination during pregnancy with adverse neonatal and maternal outcomes: A systematic review and meta-analysis. Frontiers in public health 11, 1044031. doi: 10.3389/fpubh.2023.1044031 | Unrelated |
|  | Geleto, A., Taylor, J., and Beyene, T. (2023). Interruptions in contraception and unintended pregnancy during the COVID-19 pandemic: A protocol for systematic review and meta-analysis. Women's Health 19. doi: 10.1177/17455057221147382 | Unrelated |
|  | Jackson, D., Katwan, E., Boehm, C., and Diaz, T. (2023). Use of routine health information systems to monitor disruptions of coverage of maternal, newborn, and child health services during COVID-19: A scoping review. Journal of global health 13, 06002 doi: 10.7189/jogh.13.06002 | Unrelated |
|  | Jones, K., Mallon, S., and Schnitzler, K. (2023). A Scoping Review of the Psychological and Emotional Impact of the COVID-19 Pandemic on Children and Young People. Illness, crises, and loss 31, 175-199. doi: 10.1177/10541373211047191 | Unrelated |
|  | Kearns, C., Houghton, C., Dickinson, E., Hatter, L., Bruce, P., Krishnamoorthy, S., Weatherall, M., Hills, T., Doppen, M., Ali Mirjalili, S., and Beasley, R. (2023). What variables should inform needle length choice for deltoid intramuscular injection? A systematic review. BMJ Open 13, e063530. doi: 10.1136/bmjopen-2022-063530 | Unrelated |
|  | Krumbein, H., Kummel, L. S., Fragkou, P. C., Tholken, C., Hunerbein, B. L., Reiter, R., Papathanasiou, K. A., Renz, H., and Skevaki, C. (2023). Respiratory viral co-infections in patients with COVID-19 and associated outcomes: A systematic review and meta-analysis. Reviews in medical virology 33, e2365. doi: 10.1002/rmv.2365 | Unrelated |
|  | Li, Y., Liang, H., Ding, X., Cao, Y., Yang, D., and Duan, Y. (2023). Effectiveness of COVID-19 vaccine in children and adolescents with the Omicron variant: A systematic review and meta-analysis. The Journal of infection. doi: 10.1016/j.jinf.2023.01.001 | Unrelated |
|  | Lin, C., Chen, B., Yang, Y., Li, Q., Wang, Q., Wang, M., Guo, S., and Tao, S. (2023). Association between depressive symptoms in the postpartum period and COVID-19: A meta-analysis. Journal of Affective Disorders 320, 247-253. doi: 10.1016/j.jad.2022.09.129 | Unrelated |
|  | Naeimi, R., Sepidarkish, M., Mollalo, A., Parsa, H., Mahjour, S., Safarpour, F., Almukhtar, M., Mechaal, A., Chemaitelly, H., Sartip, B., Marhoommirzabak, E., Ardekani, A., Hotez, P. J., Gasser, R. B., and Rostami, A. (2023). SARS-CoV-2 seroprevalence in children worldwide: A systematic review and meta-analysis. eClinicalMedicine 56, 101786. doi: 10.1016/j.eclinm.2022.101786 | Unrelated |
|  | Pezzullo, A. M., Axfors, C., Contopoulos-Ioannidis, D. G., Apostolatos, A., and Ioannidis, J. P. A. (2023). Age-stratified infection fatality rate of COVID-19 in the non-elderly population. Environmental research 216, 114655. doi: 10.1016/j.envres.2022.114655 | Unrelated |
|  | Pilarska, I., Bizon, M., and Sawicki, W. (2023). Influence of COVID-19 infection on placental function. Ginekologia polska 94, 79-83. doi: 10.5603/GP.a2022.0139 | Unrelated |
|  | Pradnyaandara, I. G. B. M. A., Winata, I. G. S., and Putra, I. W. A. (2023). IMPACT OF SARS-COV-2 INFECTION TO RISK AND SEVERITY OF HYPERTENSION IN PREGNANCY AND ITS OUTCOME TO THE NEW BORN. Journal of Hypertension 41, e466-e467. | Unrelated |
|  | Shafiee, A., Kohandel Gargari, O., Teymouri Athar, M. M., Fathi, H., Ghaemi, M., and Mozhgani, S.-H. (2023). COVID-19 vaccination during pregnancy: a systematic review and meta-analysis. BMC pregnancy and childbirth 23, 45. doi: 10.1186/s12884-023-05374-2 | Unrelated |
|  | Simbar, M., Nazarpour, S., and Sheidaei, A. (2023). Evaluation of pregnancy outcomes in mothers with COVID-19 infection: a systematic review and meta-analysis. Journal of Obstetrics and Gynaecology 43, 2162867. doi: 10.1080/01443615.2022.2162867 | Unrelated |
|  | Smith, E. R., Oakley, E., Grandner, G. W., Rukundo, G., Farooq, F., Ferguson, K., Baumann, S., Adams Waldorf, K. M., Afshar, Y., Ahlberg, M., Ahmadzia, H., Akelo, V., Aldrovandi, G., Bevilacqua, E., Bracero, N., Brandt, J. S., Broutet, N., Carrillo, J., Conry, J., Cosmi, E., Crispi, F., Crovetto, F., Del Mar Gil, M., Delgado-Lopez, C., Divakar, H., Driscoll, A. J., Favre, G., Fernandez Buhigas, I., Flaherman, V., Gale, C., Godwin, C. L., Gottlieb, S., Gratacos, E., He, S., Hernandez, O., Jones, S., Joshi, S., Kalafat, E., Khagayi, S., Knight, M., Kotloff, K. L., Lanzone, A., Laurita Longo, V., Le Doare, K., Lees, C., Litman, E., Lokken, E. M., Madhi, S. A., Magee, L. A., Martinez-Portilla, R. J., Metz, T. D., Miller, E. S., Money, D., Moungmaithong, S., Mullins, E., Nachega, J. B., Nunes, M. C., Onyango, D., Panchaud, A., Poon, L. C., Raiten, D., Regan, L., Sahota, D., Sakowicz, A., Sanin-Blair, J., Stephansson, O., Temmerman, M., Thorson, A., Thwin, S. S., Tippett Barr, B. A., Tolosa, J. E., Tug, N., Valencia-Prado, M., Visentin, S., von Dadelszen, P., Whitehead, C., Wood, M., Yang, H., Zavala, R., and Tielsch, J. M. (2023). Clinical risk factors of adverse outcomes among women with COVID-19 in the pregnancy and postpartum period: a sequential, prospective meta-analysis. American journal of obstetrics and gynecology 228, 161-177. doi: 10.1016/j.ajog.2022.08.038 | Unrelated |
|  | Vitoratou, D.-I., Milas, G.-P., Korovessi, P., Kostaridou, S., and Koletsi, P. (2023). Obesity as a risk factor for severe influenza infection in children and adolescents: a systematic review and meta-analysis. European journal of pediatrics 182, 363-374. doi: 10.1007/s00431-022-04689-0 | Unrelated |
|  | Wang, B., Andraweera, P., Elliott, S., Mohammed, H., Lassi, Z., Twigger, A., Borgas, C., Gunasekera, S., Ladhani, S., and Marshall, H. S. (2023). Asymptomatic SARS-CoV-2 Infection by Age: A Global Systematic Review and Meta-analysis. The Pediatric infectious disease journal 42, 232-239. doi: 10.1097/INF.0000000000003791 | Unrelated |
|  | Wu, S., Wang, L., Dong, J., Bao, Y., Li, Y., Liu, X., Xie, H., and Ying, H. (2023). The Dose- and Time-Dependent Effectiveness and Safety Associated with COVID-19 Vaccination During Pregnancy: A Systematic Review and Meta-analysis. International journal of infectious diseases : IJID : official publication of the International Society for Infectious Diseases. doi: 10.1016/j.ijid.2023.01.018 | Unrelated |
|  | Yao, X., Xu, X., Chan, K. L., Chen, S., Assink, M., and Gao, S. (2023). Associations between psychological inflexibility and mental health problems during the COVID-19 pandemic: A three-level meta-analytic review. Journal of Affective Disorders 320, 148-160. doi: 10.1016/j.jad.2022.09.116 | Unrelated |
|  | Yasuhara, J., Masuda, K., Aikawa, T., Shirasu, T., Takagi, H., Lee, S., and Kuno, T. (2023). Myopericarditis after COVID-19 mRNA Vaccination among Adolescents and Young Adults: A Systematic Review and Meta-analysis. JAMA Pediatrics 177, 42-52. doi: 10.1001/jamapediatrics.2022.4768 | Unrelated |
|  | Yonemoto, N., and Kawashima, Y. (2023). Help-seeking behaviors for mental health problems during the COVID-19 pandemic: A systematic review. Journal of Affective Disorders 323, 85-100. doi: 10.1016/j.jad.2022.11.043 | Unrelated |
|  | Yoo, L., Tsai, C. S., Heitkemper, M., and Kamp, K. (2023). Systematic review: Individual-level factors and social determinants of health impacting sleep health in individuals with inflammatory bowel disease. Journal of advanced nursing. doi: 10.1111/jan.15576 | Unrelated |
|  | Liu, J. P., Manheimer, E., and Shi, Y. (2005). Systematic review and meta-analysis on the integrative traditional Chinese and Western medicine in treating SARS. Zhongguo Zhong xi yi jie he za zhi Zhongguo Zhongxiyi jiehe zazhi = Chinese journal of integrated traditional and Western medicine / Zhongguo Zhong xi yi jie he xue hui, Zhongguo Zhong yi yan jiu yuan zhu ban 25, 1082-1088. | Unrelated |
|  | Patick, A. K. (2006). Rhinovirus chemotherapy. Antiviral Research 71, 391-396. doi: 10.1016/j.antiviral.2006.03.011 | Unrelated |
|  | Jefferson, T., Del Mar, C., Dooley, L., Ferroni, E., Al-Ansary, L. A., Bawazeer, G. A., Van Driel, M. L., Foxlee, R., and Rivetti, A. (2009). Physical interventions to interrupt or reduce the spread of respiratory viruses: Systematic review. BMJ (Online) 339, 792. doi: 10.1136/bmj.b3675 | Unrelated |
|  | Jung, K. (2010). The role of antibiotics in acute bronchitis. Respirology 15, 11. doi: 10.1111/j.1400-1843.2010.01863.x | Unrelated |
|  | Luksic, I., Kearns, P. K., Scott, F., Rudan, I., Campbell, H., and Nair, H. (2013). Viral etiology of hospitalized acute lower respiratory infections in children under 5 years of age -- a systematic review and meta-analysis. Croatian medical journal 54, 122-34. | Unrelated |
|  | Pinto, C. S., Nunes, B., Branco, M. J., and Falcao, J. M. (2013). Trends in influenza vaccination coverage in Portugal from 1998 to 2010: effect of major pandemic threats. BMC public health 13, 1130. | Unrelated |
|  | Piedra, P. A. (2015). The role of respiratory viruses and the impact of newer diagnostic testing in cystic fibrosis. Pediatric Pulmonology 50, 117. doi: 10.1002/ppul.23296 | Unrelated |
|  | Shi, T., McLean, K., Campbell, H., and Nair, H. (2015). Aetiological role of common respiratory viruses in acute lower respiratory infections in children under five years: A systematic review and meta-analysis. Journal of global health 5, 010408. doi: 10.7189/jogh.05.010408 | Unrelated |
|  | Jin, J., Zhou, Y. Z., Gan, Y. C., Song, J., and Li, W. M. (2017). Early-life respiratory infections are pivotal in the progression of asthma-a systematic review and meta-analysis. International Journal of Clinical and Experimental Medicine 10, 4256-4266. | Unrelated |
|  | The Lancet-CAMS Health Summit, 2018. The Lancet 392. | Unrelated |
|  | Ma, X., Conrad, T., Alchikh, M., Reiche, J., Schweiger, B., and Rath, B. (2018). Can we distinguish respiratory viral infections based on clinical features? A prospective pediatric cohort compared to systematic literature review. Reviews in Medical Virology 28, e1997. doi: 10.1002/rmv.1997 | Unrelated |
|  | Amsalu, R. (2019). Viral hemorrhagic fevers in pregnancy: A systematic review and meta-analysis. American Journal of Tropical Medicine and Hygiene 101, 72. doi: 10.4269/ajtmh.abstract2019 | Unrelated |
|  | Hasegawa, K., Goto, T., Hirayama, A., Laham, F. R., Mansbach, J. M., Piedra, P. A., and Camargo, C. A., Jr. (2019). Respiratory Virus Epidemiology Among US Infants With Severe Bronchiolitis: Analysis of 2 Multicenter, Multiyear Cohort Studies. The Pediatric infectious disease journal 38, e180-e183. doi: 10.1097/INF.0000000000002279 | Unrelated |
|  | Kenmoe, S., Bigna, J. J., Fatawou Modiyingi, A., Ndangang, M. S., Ngoupo, P. A., Simo, F. B. N., Tchatchouang, S., Temfack, E., and Njouom, R. (2019). Case fatality rate and viral aetiologies of acute respiratory tract infections in HIV positive and negative people in Africa: The VARIAFRICA-HIV systematic review and meta-analysis. Journal of Clinical Virology 117, 96-102. doi: 10.1016/j.jcv.2019.06.006 | Unrelated |
|  | Ahlawat, R., Kataria, A., Goyal, R., and Sethi, A. (2020). PMU48 EFFECT OF MOBILE HEALTH INTERVENTIONS ON MATERNAL AND CHILD HEALTH: A SYSTEMATIC REVIEW OF RANDOMIZED CONTROLLED TRIALS. Value in Health 23, S242. doi: 10.1016/j.jval.2020.04.822 | Unrelated |
|  | Antonio, A. C., and Pires Ferreira, M. A. (2020). PIH61 ASSEMBLING EVIDENCE ABOUT THE USEFULNESS OF HARMONIC SCALPEL IN PEDIATRIC LAPAROSCOPIC FUNDOPLICATION. Value in Health 23, S163. doi: 10.1016/j.jval.2020.04.455 | Unrelated |
|  | Cheng, X., Chen, Z., Xu, P. P., Qin, F., Jiao, X. F., Wang, Y. Y., Lin, M., Zeng, L. N., Huang, L., Yu, D., and Zhang, L. L. (2020). PIH2 EFFICACY AND SAFETY OF MIDAZOLAM ORAL SOLUTION FOR SEDATIVE-HYPNOSIS AND ANTI-ANXIETY IN CHILDREN: A SYSTEMATIC REVIEW AND META-ANALYSIS. Value in Health 23, S151. doi: 10.1016/j.jval.2020.04.398 | Unrelated |
|  | Dahine, J., Hebert, P. C., Ziegler, D., Chenail, N., Ferrari, N., and Hebert, R. (2020). Practices in Triage and Transfer of Critically Ill Patients: A Qualitative Systematic Review of Selection Criteria. Critical care medicine. doi: 10.1097/CCM.0000000000004624 | Unrelated |
|  | De Corso, E., Lucidi, D., Cantone, E., Ottaviano, G., Di Cesare, T., Seccia, V., Paludetti, G., and Galli, J. (2020). Clinical Evidence and Biomarkers Linking Allergy and Acute or Chronic Rhinosinusitis in Children: a Systematic Review. Current Allergy and Asthma Reports 20, 68. doi: 10.1007/s11882-020-00967-9 | Unrelated |
|  | Diriba, K., Awulachew, E., and Getu, E. (2020). The effect of coronavirus infection (SARS-CoV-2, MERS-CoV, and SARS-CoV) during pregnancy and the possibility of vertical maternal-fetal transmission: a systematic review and meta-analysis. European journal of medical research 25, 39. doi: 10.1186/s40001-020-00439-w | Unrelated |
|  | Mailhot, G., and White, J. H. (2020). Vitamin D and immunity in infants and children. Nutrients 12, 1233. doi: 10.3390/nu12051233 | Unrelated |
|  | Mair, M., Singhavi, H., Pai, A., Singhavi, J., Gandhi, P., Conboy, P., Baker, A., and Das, S. (2020). A meta-analysis of 67 studies with presenting symptoms & laboratory tests of COVID-19 patients. The Laryngoscope. doi: 10.1002/lary.29207 | Unrelated |
|  | Mohammadi, M., Rajabnia, M., and Abdehagh, M. (2020). Gastrointestinal manifestations of COVID-19 in adults: A review article. Scientific Journal of Kurdistan University of Medical Sciences 25, 42-55. | Unrelated |
|  | Sodhi, M., Khosrow-Khavar, F., FitzGerald, J. M., and Etminan, M. (2020). Non-steroidal Anti-inflammatory Drugs and the Risk of Pneumonia Complications: A Systematic Review. Pharmacotherapy 40, 970-977. doi: 10.1002/phar.2451 | Unrelated |
|  | Vatturi, M., Dovari, A., Inuganti, B., Soni, P., Hyderboini, R., Veeranki, P., Manne, M., Rajpal, S., Chidirala, S., and Goyal, R. (2020). PND11 EFFICACY AND SAFETY OF BREXANOLONE IN THE TREATMENT OF POSTPARTUM DEPRESSION: A SYSTEMATIC REVIEW AND META-ANALYSIS. Value in Health 23, S260. doi: 10.1016/j.jval.2020.04.904 | Unrelated |
|  | Zhang, L., Peres, T. G., Silva, M. V. F., and Camargos, P. (2020). What we know so far about Coronavirus Disease 2019 in children: A meta-analysis of 551 laboratory-confirmed cases. Pediatric Pulmonology 55, 2115-2127. doi: 10.1002/ppul.24869 | Unrelated |
|  | Ali, A. S., Hasan, S. S., Kow, C. S., and Merchant, H. A. (2021). Lactoferrin reduces the risk of respiratory tract infections: A meta-analysis of randomized controlled trials. Clinical nutrition ESPEN 45, 26-32. doi: 10.1016/j.clnesp.2021.08.019 | Unrelated |
|  | Fernandes, A. K., Wilson, S., Nalin, A. P., Philip, A., Gruber, L., Kwizera, E., Sydelko, B. S., Forbis, S. G., and Lauden, S. (2021). Pediatric Family-Centered Rounds and Humanism: A Systematic Review and Qualitative Meta-analysis. Hospital pediatrics. doi: 10.1542/hpeds.2020-000240 | Unrelated |
|  | Fyfe-Johnson, A. L., Marcuse, E. K., Tandon, P., Hazlehurst, M., Bratman, G. N., Thomas, R., Perrins, S. P., and Garrett, K. (2021). Nature contact and children's health: A systematic review. Pediatrics 147, 50-52. doi: 10.1542/peds.147.3-MeetingAbstract.50 | Unrelated |
|  | Gunnar, M. R., Reid, B. M., Donzella, B., Miller, Z. R., Gardow, S., Tsakonas, N. C., Thomas, K. M., DeJoseph, M., and Bendezu, J. J. (2021). Validation of an online version of the Trier Social Stress Test in a study of adolescents. Psychoneuroendocrinology 125, 105111. doi: 10.1016/j.psyneuen.2020.105111 | Unrelated |
|  | Hughes, K., Ford, K., Bellis, M. A., Glendinning, F., Harrison, E., and Passmore, J. (2021). Health and financial costs of adverse childhood experiences in 28 European countries: a systematic review and meta-analysis. The Lancet Public Health 6, e848-e857. doi: 10.1016/S2468-2667%2821%2900232-2 | Unrelated |
|  | Khan, A., Feulefack, J., and Sergi, C. (2021). Parental Exposure to Pesticides and Childhood Brain Cancer. A Systematic Review and Meta-Analysis. Pediatric Blood and Cancer 68. doi: 10.1002/pbc.29349 | Unrelated |
|  | Khan, A., Sergi, C., and Feulefack, J. (2021). Pesticides Exposure and Wilms Tumor in Childhood. A Prisma-Based Systematic Review with Meta-Analysis. Pediatric Blood and Cancer 68. doi: 10.1002/pbc.29349 | Unrelated |
|  | Lau, N., Colt, S. F., Waldbaum, S., O'Daffer, A., Fladeboe, K., Yi-Frazier, J. P., McCauley, E., and Rosenberg, A. R. (2021). Telemental health for youth with chronic illnesses: Systematic review. JMIR Mental Health 8, e30098. doi: 10.2196/30098 | Unrelated |
|  | Morelius, E., Robinson, S., Arabiat, D., and Whitehead, L. (2021). Digital Interventions to Improve Health Literacy among Parents of Children Aged 0 to 12 Years with a Health Condition: Systematic Review. Journal of Medical Internet Research 23, e31665. doi: 10.2196/31665 | Unrelated |
|  | Munyuzangabo, M., Gaffey, M. F., Khalifa, D. S., Als, D., Ataullahjan, A., Kamali, M., Jain, R. P., Meteke, S., Radhakrishnan, A., Shah, S., Siddiqui, F. J., and Bhutta, Z. A. (2021). Delivering maternal and neonatal health interventions in conflict settings: a systematic review. BMJ global health 5. doi: 10.1136/bmjgh-2020-003750 | Unrelated |
|  | Rankine-Mullings, A. E., and Owusu-Ofori, S. (2021). Prophylactic antibiotics for preventing pneumococcal infection in children with sickle cell disease. The Cochrane database of systematic reviews 3, CD003427. doi: 10.1002/14651858.CD003427.pub5 | Unrelated |
|  | Russo, G., Jesus, T. S., Deane, K., Osman, A. Y., and McCoy, D. (2021). Epidemics, Lockdown Measures and Vulnerable Populations: A Mixed-Methods Systematic Review of the Evidence of Impacts on Mother and Child Health in Low-and Lower-Middle-Income Countries. International journal of health policy and management. doi: 10.34172/ijhpm.2021.155 | Unrelated |
|  | Sjostrand, A., Kefalianos, E., Hofslundsengen, H., Guttormsen, L. S., Kirmess, M., Lervag, A., Hulme, C., and Bottegaard Naess, K. A. (2021). Non-pharmacological interventions for stuttering in children six years and younger. Cochrane Database of Systematic Reviews 2021, CD013489. doi: 10.1002/14651858.CD013489.pub2 | Unrelated |
|  | Alexander, E. C., and Deep, A. (2022). Characterization of a Hepatitis Outbreak in Children, 2021 to 2022. JAMA network open 5, e2237091. doi: 10.1001/jamanetworkopen.2022.37091 | Unrelated |
|  | Barisic, N., Turudic, D., Maric, L. S., and Tesovic, G. (2022). Vaccination in pediatric acquired inflammatory immune-mediated neuromuscular disorders. European Journal of Paediatric Neurology 36, 159-176. doi: 10.1016/j.ejpn.2021.12.014 | Unrelated |
|  | Campisi, S. C., Zasowski, C., Bradley-Ridout, G., Szatmari, P., and Korczak, D. J. (2022). 1.25 Omega-3 Fatty Acid Supplementation for Depression in Children and Adolescents: A Systematic Review and Meta-Analysis. Journal of the American Academy of Child and Adolescent Psychiatry 61, S149. doi: 10.1016/j.ejpn.2021.12.014 | Unrelated |
|  | Chen, X., and Yang, L. (2022). A systematic review and meta-analysis of the relationship between T-lymphocytes and respiratory tract infection in children. Advances in clinical and experimental medicine: official organ Wroclaw Medical University. doi: 10.17219/acem/154881 | Unrelated |
|  | Dunn, C., and Sicouri, G. (2022). The Relationship Between Loneliness and Depressive Symptoms in Children and Adolescents: A Meta-Analysis. Behaviour Change 39, 134-145. doi: 10.1017/bec.2022.13 | Unrelated |
|  | Eirich, R., McArthur, B. A., Anhorn, C., McGuinness, C., Christakis, D. A., and Madigan, S. (2022). Association of Screen Time with Internalizing and Externalizing Behavior Problems in Children 12 Years or Younger: A Systematic Review and Meta-analysis. JAMA Psychiatry 79, 393-405. doi: 10.1001/jamapsychiatry.2022.0155 | Unrelated |
|  | Fischer, R., and Hartle, L. (2022). Effective interventions to reduce loneliness in big cities. Current opinion in psychiatry. doi: 10.1097/YCO.0000000000000844 | Unrelated |
|  | Hodder, R. K., O'Brien, K. M., Lorien, S., Wolfenden, L., Moore, T. H. M., Hall, A., Yoong, S. L., and Summerbell, C. (2022). Interventions to prevent obesity in school-aged children 6-18 years: An update of a Cochrane systematic review and meta-analysis including studies from 2015-2021. eClinicalMedicine 54, 101635. doi: 10.1016/j.eclinm.2022.101635 | Unrelated |
|  | Khan, A., Feulefack, J., and Sergi, C. M. (2022). Pre-conceptional and prenatal exposure to pesticides and pediatric neuroblastoma. A meta-analysis of nine studies. Environmental Toxicology and Pharmacology 90, 103790. doi: 10.1016/j.etap.2021.103790 | Unrelated |
|  | Minotti, C., Barbieri, E., Doni, D., Impieri, C., Giaquinto, C., and Dona, D. (2022). Anti-infective Medicines Use in Children and Neonates With Pre-existing Kidney Dysfunction: A Systematic Review. Frontiers in Pediatrics 10, 868513. doi: 10.3389/fped.2022.868513 | Unrelated |
|  | Oliveira da Silva Kist, M. L., Hanzen Andrades, G. R., Drumond Costa, C. A., Crestani, F., and Ramos Garcia, P. C. (2022). Weight excess association with severity in children and adolescents with COVID-19: A systematic review. Clinical Nutrition ESPEN 49, 114-120. doi: 10.1016/j.clnesp.2022.04.005 | Unrelated |
|  | O'Connor, A., Dennett, E., Hasan, M., Stovold, E., and Carson-Chahhoud, K. (2022). Home-based educational interventions for children with asthma, a Cochrane review. Respirology 27, 39. doi: 10.1111/resp.14216 | Unrelated |
|  | Pang, L., Liu, H., Liu, Z., Tan, J., Zhou, L. Y., Qiu, Y., Lin, X., He, J., Li, X., Lin, S., Ghosh, S., Mao, R., and Chen, M. (2022). Role of Telemedicine in Inflammatory Bowel Disease: Systematic Review and Meta-analysis of Randomized Controlled Trials. Journal of Medical Internet Research 24, e28978. doi: 10.2196/28978 | Unrelated |
|  | Park, J. J., Narayanan, S., Tiefenbach, J., Luksic, I., Ale, B. M., Adeloye, D., and Rudan, I. (2022). Estimating the global and regional burden of meningitis in children caused by Haemophilus influenzae type b: A systematic review and meta-analysis. Journal of global health 12, 04014. doi: 10.7189/jogh.12.04014 | Unrelated |
|  | Pratt, M. T. G., Abdalla, T., Richmond, P. C., Moore, H. C., Snelling, T. L., Blyth, C. C., and Bhuiyan, M. U. (2022). Prevalence of respiratory viruses in community-acquired pneumonia in children: a systematic review and meta-analysis. The Lancet Child and Adolescent Health 6, 555-570. doi: 10.1016/S2352-4642%2822%2900092-X | Unrelated |
|  | Rahman, A. E., Hossain, A. T., Nair, H., Chisti, M. J., Dockrell, D., Arifeen, S. E., and Campbell, H. (2022). Prevalence of hypoxaemia in children with pneumonia in low-income and middle-income countries: a systematic review and meta-analysis. The Lancet Global Health 10, e348-e359. doi: 10.1016/S2214-109X%2821%2900586-6 | Unrelated |
|  | Ringden, O., Moll, G., Gustafsson, B., and Sadeghi, B. (2022). Mesenchymal Stromal Cells for Enhancing Hematopoietic Engraftment and Treatment of Graft-Versus-Host Disease, Hemorrhages and Acute Respiratory Distress Syndrome. Frontiers in immunology 13, 839844. doi: 10.3389/fimmu.2022.839844 | Unrelated |
|  | Shou, S., Xiu, S., Li, Y., Zhang, N., Yu, J., Ding, J., and Wang, J. (2022). Efficacy of Online Intervention for ADHD: A Meta-Analysis and Systematic Review. Frontiers in psychology 13, 854810. doi: 10.3389/fpsyg.2022.854810 | Unrelated |
|  | Song, P., Adeloye, D., Salim, H., Dos Santos, J. P., Campbell, H., Sheikh, A., and Rudan, I. (2022). Global, regional, and national prevalence of asthma in 2019: a systematic analysis and modelling study. Journal of global health 12, 04052. doi: 10.7189/jogh.12.04052 | Unrelated |
|  | Tavormina, M. G. M., and Tavormina, R. (2022). DEPRESSION IN EARLY CHILDHOOD. Psychiatria Danubina 34, 64-70. doi: 10.24869/psyd.2022.64 | Unrelated |
|  | Tong, T., Yao, X., Lin, Z., Tao, Y., Xu, J., Xu, X., Fang, Z., Geng, Z., Fu, S., Wang, W., Xie, C., Zhang, Y., Wang, Y., and Gong, F. (2022). Similarities and differences between MIS-C and KD: a systematic review and meta-analysis. Pediatric Rheumatology 20, 112. doi: 10.1186/s12969-022-00771-x | Unrelated |
|  | Xiao, W., Wu, J., Yip, J., Shi, Q., Peng, L., Lei, Q. E., and Ren, Z. (2022). The Relationship Between Physical Activity and Mobile Phone Addiction Among Adolescents and Young Adults: Systematic Review and Meta-analysis of Observational Studies. JMIR public health and surveillance 8, e41606. doi: 10.2196/41606 | Unrelated |
|  | Yambao, M. L. (2022). Diagnostic Accuracy of C-Reactive Protein and Procalcitonin in the Diagnosis of Pediatric Community-Acquired Pneumonia in Children: A Meta-Analysis. Pediatric Pulmonology 57, S115. doi: 10.1002/ppul.25963 | Unrelated |
|  | Yasuhara, J., Masuda, K., Watanabe, K., Shirasu, T., Takagi, H., Sumitomo, N., Lee, S., and Kuno, T. (2022). Longitudinal Cardiac Outcomes of Multisystem Inflammatory Syndrome in Children: A Systematic Review and Meta-Analysis. Pediatric Cardiology. doi: 10.1007/s00246-022-03052-2 | Unrelated |
|  | Zhu, Y., Xia, Y., Pickering, J., Bowen, A. C., and Short, K. R. (2022). The Role of Children in SARS-CoV-2 Variant of Concerns Transmission within Households: A Meta-analysis. medRxiv. doi: 10.1101/2022.07.21.22277914 | Unrelated |
|  | Hon, K. L., Leung, A. K. C., Wong, A. H. C., Dudi, A., and Leung, K. K. Y. (2023). Respiratory Syncytial Virus is the Most Common Causative Agent of Viral Bronchiolitis in Young Children: An Updated Review. Current Pediatric Reviews 19, 139-149. doi: 10.2174/1573396318666220810161945 | Unrelated |
|  | Idoiaga Mondragon, N., Fernandez, I. L., Ozamiz-Etxebarria, N., Villagrasa, B., and Santabarbara, J. (2023). PTSD (posttraumatic stress disorder) in Teachers: A Mini Meta-Analysis during COVID-19. International Journal of Environmental Research and Public Health 20, 1802. doi: 10.3390/ijerph20031802 | Unrelated |
|  | Kandiah, T., Li, X., Macmillan, Y., and Malvankar-Mehta, M. S. (2023). Access to Pediatric Eye Care During a Pandemic: Systematic Review and Meta-Analysis. Pediatric Annals 52, e68-e75. doi: 10.3928/19382359-20230130-01 | Unrelated |
|  | Larouche, R., Kleinfeld, M., Charles Rodriguez, U., Hatten, C., Hecker, V., Scott, D. R., Brown, L. M., Onyeso, O. K., Sadia, F., and Shimamura, H. (2023). Determinants of Outdoor Time in Children and Youth: A Systematic Review of Longitudinal and Intervention Studies. International Journal of Environmental Research and Public Health 20, 1328. doi: 10.3390/ijerph20021328 | Unrelated |
|  | Lopez-Gil, J. F., Garcia-Hermoso, A., Smith, L., Firth, J., Trott, M., Mesas, A. E., Jimenez-Lopez, E., Gutierrez-Espinoza, H., Tarraga-Lopez, P. J., and Victoria-Montesinos, D. (2023). Global Proportion of Disordered Eating in Children and Adolescents: A Systematic Review and Meta-analysis. JAMA pediatrics. doi: 10.1001/jamapediatrics.2022.5848 | Unrelated |
|  | Aggarwal, N., Garg, M., Dwarakanathan, V., Gautam, N., Kumar, S. S., Jadon, R. S., Gupta, M., and Ray, A. (2020). Diagnostic accuracy of non-contact infrared thermometers and thermal scanners: A systematic review and meta-analysis. Journal of Travel Medicine 27, 1-17. doi: 10.1093/JTM/TAAA193 | Unrelated |
|  | Anonymous (2020). The Catalogue of Journal of Sport and Health Science 2020. Journal of Sport and Health Science 9, 705-708. doi: 10.1016/j.jshs.2020.10.003 | Unrelated |
|  | Beaudry, G., Zhong, S., Whiting, D., Javid, B., Frater, J., and Fazel, S. (2020). Managing outbreaks of highly contagious diseases in prisons: a systematic review. BMJ global health 5. doi: 10.1136/bmjgh-2020-003201 | Unrelated |
|  | Dorantes-Acosta, E., Avila-Montiel, D., Klunder-Klunder, M., Juarez-Villegas, L., and Marquez-Gonzalez, H. (2020). Survival and Complications in Pediatric Patients With Cancer and COVID-19: A Meta-Analysis. Frontiers in Oncology 10, 608282. doi: 10.3389/fonc.2020.608282 | Unrelated |
|  | Gavriilidis, P., and Pai, M. (2020). The Impact of COVID-19 Global Pandemic on Morbidity and Mortality of Liver Transplant Recipients Children and Adults: A Systematic Review of Case Series. Journal of clinical medicine research 12, 404-408. doi: 10.14740/jocmr4223 | Unrelated |
|  | Goel, R., Cohn, C. S., and Gorham, J. D. (2020). Development of a database of registered clinical trials using convalescent plasma for treatment of COVID-19: Is data harmonization possible? Transfusion 60, 288A-289A. doi: 10.1111/trf.16084 | Unrelated |
|  | He, Z., Wu, J., Pan, J., Ma, H., Zhang, C., and Ming, W. K. (2020). PIH63 THE EFFICACY OF TREATMENTS FOR ATOPIC DERMATITIS IN CHILDREN: A NETWORK META-ANALYSIS. Value in Health 23, S163. doi: 10.1016/j.jval.2020.04.457 | Unrelated |
|  | Hennegan, K., Silber, A., Dehipawala, S., Chithran, K., and Lockhart, D. (2020). PIH67 EVALUATING THE BURDEN OF ILLNESS OF PEDIATRIC PLATINUM-INDUCED HEARING LOSS: A SYSTEMATIC LITERATURE REVIEW. Value in Health 23, S164. doi: 10.1016/j.jval.2020.04.461 | Unrelated |
|  | Hernandez, N., Laignelet, H., Reyes Sanchez, J. M., Rincon Martinez, L. M., Castano Gamboa, N., Ruiz, F., and Gutierrez Ardila, M. V. (2020). PMS52 EPIDEMIOLOGY OF DERMATITIS ATOPIC IN AMERICA: SYSTEMATIC REVIEW. Value in Health 23, S224. doi: 10.1016/j.jval.2020.04.738 | Unrelated |
|  | Kenmoe, S., Kengne-Nde, C., Ebogo-Belobo, J. T., Mbaga, D. S., Fatawou Modiyinji, A., and Njouom, R. (2020). Systematic review and meta-analysis of the prevalence of common respiratory viruses in children < 2 years with bronchiolitis in the pre-COVID-19 pandemic era. PloS one 15, e0242302. doi: 10.1371/journal.pone.0242302 | Unrelated |
|  | Khatami, F., Saatchi, M., Zadeh, S. S. T., Aghamir, Z. S., Shabestari, A. N., Reis, L. O., and Aghamir, S. M. K. (2020). A meta-analysis of accuracy and sensitivity of chest CT and RT-PCR in COVID-19 diagnosis. Scientific reports 10, 22402. doi: 10.1038/s41598-020-80061-2 | Unrelated |
|  | Koh, W. C., Naing, L., Chaw, L., Rosledzana, M. A., Alikhan, M. F., Jamaludin, S. A., Amin, F., Omar, A., Shazli, A., Griffith, M., Pastore, R., and Wong, J. (2020). What do we know about SARS-CoV-2 transmission? A systematic review and meta-analysis of the secondary attack rate and associated risk factors. PLoS ONE 15, e0240205. doi: 10.1371/journal.pone.0240205 | Unrelated |
|  | Komolafe, T. E., Agbo, J., Olaniyi, E. O., Komolafe, K., and Yang, X. (2020). Prevalence of covid-19 diagnostic output with chest computed tomography: A systematic review and meta-analysis. Diagnostics 10, 1023. doi: 10.3390/diagnostics10121023 | Unrelated |
|  | Kotlyar, A., Grechukhina, O., Chen, A., Popkhadze, S., Grimshaw, A., Tal, O., Taylor, H. S., and Tal, R. (2020). Vertical Transmission of COVID-19: A Systematic Review and Meta-analysis. American journal of obstetrics and gynecology. doi: 10.1016/j.ajog.2020.07.049 | Unrelated |
|  | Kumbargere Nagraj, S., Eachempati, P., Paisi, M., Nasser, M., Sivaramakrishnan, G., and Verbeek, J. H. (2020). Interventions to reduce contaminated aerosols produced during dental procedures for preventing infectious diseases. The Cochrane database of systematic reviews 10, CD013686. doi: 10.1002/14651858.CD013686.pub2 | Unrelated |
|  | Liu, C., He, Y., Liu, L., Li, F., and Shi, Y. (2020). Children with COVID-19 behaving milder may challenge the public policies: A systematic review and meta-analysis. BMC Pediatrics 20, 410. doi: 10.1186/s12887-020-02316-1 | Unrelated |
|  | Lu, S., Zhou, Q., Huang, L., Shi, Q., Zhao, S., Wang, Z., Li, W., Tang, Y., Ma, Y., Luo, X., Fukuoka, T., Ahn, H. S., Lee, M. S., Luo, Z., Liu, E., Chen, Y., Zhou, C., and Peng, D. (2020). Effectiveness and safety of glucocorticoids to treat COVID-19: A rapid review and meta-analysis. Annals of Translational Medicine 8, 627. doi: 10.21037/atm-20-3307 | Unrelated |
|  | Medeiros, G. C. B. S. D., Nunes, A. C. D. F., Azevedo, K. P. M. D., De Oliveira Segundo, V. H., Santos, G. M., Mata, A. N. D. S., Pimenta, I. D., Bezerra, I. N. M., Braga, L. P., Capucho, H. C., Piuvezam, M. R., Barbosa Filho, V. C., Leitao, J. C., Martinez, D. G., and Piuvezam, G. (2020). The Control and Prevention of COVID-19 Transmission in Children: A Protocol for Systematic Review and Meta-analysis. Medicine (United States) 99, E21393. doi: 10.1097/MD.0000000000021393 | Unrelated |
|  | Monette, A., and Mouland, A. J. (2020). Zinc and copper ions differentially regulate prion-like phase separation dynamics of pan-virus nucleocapsid biomolecular condensates. Viruses 12, 1179. doi: 10.3390/v12101179 | Unrelated |
|  | Moutchia, J., Pokharel, P., Kerri, A., McGaw, K., Uchai, S., Nji, M., and Goodman, M. (2020). Clinical laboratory parameters associated with severe or critical novel coronavirus disease 2019 (COVID-19): A systematic review and meta-analysis. PLoS ONE 15, e0239802. doi: 10.1371/journal.pone.0239802 | Unrelated |
|  | Murphy, E. P., Fenelon, C., Murphy, R. P., O'Sullivan, M. D., Pomeroy, E., Sheehan, E., and Moore, D. P. (2020). Are Virtual Fracture Clinics during the COVID-19 Pandemic a Potential Alternative for Delivering Fracture Care? A Systematic Review. Clinical Orthopaedics and Related Research 478, 2610-2621. doi: 10.1097/CORR.0000000000001388 | Unrelated |
|  | Mutiawati, E., Syahrul, S., Fahriani, M., Fajar, J. K., Mamada, S. S., Maliga, H. A., Samsu, N., Ilmawan, M., Purnamasari, Y., Asmiragani, A. A., Ichsan, I., Emran, T. B., Rabaan, A. A., Masyeni, S., Nainu, F., and Harapan, H. (2020). Global prevalence and pathogenesis of headache in COVID-19: A systematic review and meta-analysis. F1000Research 9, 1316. doi: 10.12688/f1000research.27334.2 | Unrelated |
|  | Nelson, A., and Anderson, M. (2020). A Systematic Review Exploring Pre-COVID-19 Telehealthcare Models Used in the Management of Patients with Rheumatological Disease. Arthritis and Rheumatology 72, 1220-1221. doi: 10.1002/art.41538 | Unrelated |
|  | Nussbaumer-Streit, B., Mayr, V., Dobrescu, A. I., Chapman, A., Persad, E., Klerings, I., Wagner, G., Siebert, U., Ledinger, D., Zachariah, C., and Gartlehner, G. (2020). Quarantine alone or in combination with other public health measures to control COVID-19: a rapid review. The Cochrane database of systematic reviews 9, CD013574. doi: 10.1002/14651858.CD013574.pub2 | Unrelated |
|  | Paludan-Muller, A. S., Boesen, K., Klerings, I., Jorgensen, K. J., and Munkholm, K. (2020). Hand cleaning with ash for reducing the spread of viral and bacterial infections: a rapid review. Cochrane Database of Systematic Reviews 2020, CD013597. doi: 10.1002/14651858.CD013597 | Unrelated |
|  | Pande, A., Lamba, N., Mammi, M., Shui, C., Gebrehiwet, P., Trenary, A., Doucette, J., Papatheodorou, S., Bunevicius, A., Smith, T. R., and Mekary, R. (2020). PND7 ENDOSCOPIC THIRD VENTRICULOSTOMY VERSUS VENTRICULOPERITONEAL SHUNT IN PEDIATRIC AND ADULT POPULATION: A SYSTEMATIC REVIEW AND META-ANALYSIS. Value in Health 23, S259. doi: 10.1016/j.jval.2020.04.900 | Unrelated |
|  | Pettirosso, E., Giles, M., Cole, S., and Rees, M. (2020). COVID-19 and pregnancy: A review of clinical characteristics, obstetric outcomes and vertical transmission. Australian and New Zealand Journal of Obstetrics and Gynaecology 60, 640-659. doi: 10.1111/ajo.13204 | Unrelated |
|  | Rodrigo, C., Fernando, S. D., and Rajapakse, S. (2020). Clinical evidence for repurposing chloroquine and hydroxychloroquine as antiviral agents: a systematic review. Clinical microbiology and infection: the official publication of the European Society of Clinical Microbiology and Infectious Diseases. doi: 10.1016/j.cmi.2020.05.016 | Unrelated |
|  | Salameh, J.-P., Leeflang, M. M., Hooft, L., Islam, N., McGrath, T. A., van der Pol, C. B., Frank, R. A., Prager, R., Hare, S. S., Dennie, C., Spijker, R., Deeks, J. J., Dinnes, J., Jenniskens, K., Korevaar, D. A., Cohen, J. F., Van den Bruel, A., Takwoingi, Y., van de Wijgert, J., Damen, J. A., Wang, J., and McInnes, M. D. (2020). Thoracic imaging tests for the diagnosis of COVID-19. The Cochrane database of systematic reviews 9, CD013639. doi: 10.1002/14651858.CD013639.pub2 | Unrelated |
|  | Sankova, M. V., Kytko, O. V., Meylanova, R. D., Vasil'ev, Y. L., and Nelipa, M. V. (2020). Possible prospects for using modern magnesium preparations for increasing stress resistance during COVID-19 pandemic. Research Results in Pharmacology 6, 65-76. doi: 10.3897/RRPHARMACOLOGY.6.59407 | Unrelated |
|  | Smith, L., Butler, L., Tully, M. A., Jacob, L., Barnett, Y., Lopez-Sanchez, G. F., Lopez-Bueno, R., Shin, J. I., McDermott, D., Pfeifer, B. A., Pizzol, D., and Koyanagi, A. (2020). Hand-Washing Practices among Adolescents Aged 12-15 Years from 80 Countries. International journal of environmental research and public health 18. doi: 10.3390/ijerph18010138 | Unrelated |
|  | Stegeman, I., Ochodo, E. A., Guleid, F., Holtman, G. A., Yang, B., Davenport, C., Deeks, J. J., Dinnes, J., Dittrich, S., Emperador, D., and et al. (2020). Routine laboratory testing to determine if a patient has COVID‐19. Cochrane Database of Systematic Reviews. doi: 10.1002/14651858.CD013787 | Unrelated |
|  | Struyf, T., Deeks, J. J., Dinnes, J., Takwoingi, Y., Davenport, C., Leeflang, M. M. G., Spijker, R., Hooft, L., Emperador, D., Dittrich, S., Domen, J., Horn, S. R. A., and Van den Bruel, A. (2020). Signs and symptoms to determine if a patient presenting in primary care or hospital outpatient settings has COVID-19 disease. Cochrane Database of Systematic Reviews 2020, CD013665. doi: 10.1002/14651858.CD013665 | Unrelated |
|  | Syangtan, G., Bista, S., Dawadi, P., Rayamajhee, B., Shrestha, L. B., Tuladhar, R., and Joshi, D. R. (2020). Asymptomatic SARS-CoV-2 Carriers: A Systematic Review and Meta-Analysis. Frontiers in public health 8, 587374. doi: 10.3389/fpubh.2020.587374 | Unrelated |
|  | Utami, R., Iswati, S., Waloejo, C. S., and Pandin, M. G. R. (2020). Management of parenting preparedness at home in COVID-2019 pandemic based on individual and family self-management theory (IFSMT): A Systematic Review. Systematic Reviews in Pharmacy 11, 826-835. doi: 10.31838/SRP.2020.7.88 | Unrelated |
|  | Varrassi, G., Pergolizzi, J., Nalamasu, R., Quang, J. A. L., Colucci, R., Breve, F., Magnusson, P., Mariano, D., and Christo, P. J. (2020). Ibuprofen safety: A look back on 50 years. Postgraduate Medicine 132, 32-33. doi: 10.1080/00325481.2020.1824967 | Unrelated |
|  | Vazquez-Cornejo, E. (2020). Considerations on the use of antihypertensive blockers of the renin-angiotensin system in adults and children in the face of the covid-19 pandemic. Boletin Medico del Hospital Infantil de Mexico 77, 274-281. doi: 10.24875/BMHIM.20000158 | Unrelated |
|  | Viner, R. M., Mytton, O. T., Bonell, C., Melendez-Torres, G. J., Ward, J., Hudson, L., Waddington, C., Thomas, J., Russell, S., van der Klis, F., Koirala, A., Ladhani, S., Panovska-Griffiths, J., Davies, N. G., Booy, R., and Eggo, R. M. (2020). Susceptibility to SARS-CoV-2 Infection Among Children and Adolescents Compared With Adults: A Systematic Review and Meta-analysis. JAMA pediatrics. doi: 10.1001/jamapediatrics.2020.4573 | Unrelated |
|  | Wang, J.-G., Cui, H.-R., Tang, H.-B., and Deng, X.-L. (2020). Gastrointestinal symptoms and fecal nucleic acid testing of children with 2019 coronavirus disease: a systematic review and meta-analysis. Scientific reports 10, 17846. doi: 10.1038/s41598-020-74913-0 | Unrelated |
|  | Wang, Y., Zhang, Y., Chen, X., Xue, K., Zhang, T., and Ren, X. (2020). Evaluating the efficacy and safety of bromhexine hydrochloride tablets in treating pediatric COVID-19: A protocol for meta-analysis and systematic review. Medicine 99, e22114. doi: 10.1097/MD.0000000000022114 | Unrelated |
|  | Wong, M. C., Huang, J., Lai, C., Ng, R., Chan, F. K. L., and Chan, P. K. S. (2020). Detection of SARS-CoV-2 RNA in fecal specimens of patients with confirmed COVID-19: a meta-analysis. The Journal of infection. doi: 10.1016/j.jinf.2020.06.012 | Unrelated |
|  | Xu, W., Li, X., Dozier, M., He, Y., Kirolos, A., Lang, Z., Mathews, C., Siegfried, N., and Theodoratou, E. (2020). What is the evidence for transmission of COVID-19 by children in schools? A living systematic review. Journal of global health 10, 021104. doi: 10.7189/jogh.10.021104 | Unrelated |
|  | Zhou, K.-L., Dong, S., Wang, K., Fu, G.-B., Niu, Y., Xue, X.-N., and Guo, S. (2020). Pediatric massage therapy for restoring pediatric lung function from COVID-19: A protocol for systematic review and meta-analysis. Medicine 99, e21581. doi: 10.1097/MD.0000000000021581 | Unrelated |
|  | Al-Hadidi, S. H., Alhussain, H., Abdel Hadi, H., Johar, A., Yassine, H. M., Al Thani, A. A., and Eltai, N. O. (2021). The Spectrum of Antibiotic Prescribing during COVID-19 Pandemic: A Systematic Literature Review. Microbial Drug Resistance 27, 1705-1725. doi: 10.1089/mdr.2020.0619 | Unrelated |
|  | Bedi, G., Vyas, K. S., Chung, M. T., Morrison, S. D., Asaad, M., and Mardini, S. (2021). Telemedicine in International Cleft Care: A Systematic Review. Cleft Palate-Craniofacial Journal 58, 1547-1555. doi: 10.1177/1055665621989140 | Unrelated |
|  | Cai, C., Peng, Y., Shen, E., Huang, Q., Chen, Y., Liu, P., Guo, C., Feng, Z., Gao, L., Zhang, X., Gao, Y., Liu, Y., Han, Y., Zeng, S., and Shen, H. (2021). A comprehensive analysis of the efficacy and safety of COVID-19 vaccines. Molecular therapy: the journal of the American Society of Gene Therapy 29, 2794-2805. doi: 10.1016/j.ymthe.2021.08.001 | Unrelated |
|  | Chan, K. W., Yu, K. Y., Lee, P. W., Lai, K. N., and Tang, S. C. W. (2021). Global REnal Involvement of CORonavirus Disease 2019 (RECORD): A Systematic Review and Meta-Analysis of Incidence, Risk Factors, and Clinical Outcomes. Frontiers in Medicine 8, 678200. doi: 10.3389/fmed.2021.678200 | Unrelated |
|  | Fahim, S. M., Hossain, M. S., Sen, S., Das, S., Hosssain, M., Ahmed, T., Mustafizur Rahman, S. M., Rahman, M. K., and Alam, S. (2021). Nutrition and Food Security in Bangladesh: Achievements, Challenges, and Impact of the COVID-19 Pandemic. Journal of Infectious Diseases 224, S901-S909. doi: 10.1093/infdis/jiab473 | Unrelated |
|  | Gomez-Carballa, A., Barral-Arca, R., Cebey-Lopez, M., Bello, X., Pardo-Seco, J., Martinon-Torres, F., and Salas, A. (2021). Identification of a minimal 3-transcript signature to differentiate viral from bacterial infection from best genome-wide host rna biomarkers: A multi-cohort analysis. International Journal of Molecular Sciences 22, 1-11. doi: 10.3390/ijms22063148 | Unrelated |
|  | Gonzalez Rodriguez, P., Perez-Moneo Agapito, B., Albi Rodriguez, M. S., Aizpurua Galdeano, P., Aparicio Rodrigo, M., Fernandez Rodriguez, M. M., Esparza Olcina, M. J., and Ochoa Sangrador, C. (2021). COVID-19: Critical appraisal of the evidence. Anales de pediatria 95, 207.e1-207.e13. doi: 10.1016/j.anpede.2021.05.003 | Unrelated |
|  | Grudlewska-Buda, K., Wiktorczyk-Kapischke, N., Walecka-Zacharska, E., Kwiecinska-Pirog, J., Buszko, K., Leis, K., Juszczuk, K., Gospodarek-Komkowska, E., and Skowron, K. (2021). Sars-cov-2-morphology, transmission and diagnosis during pandemic, review with element of meta-analysis. Journal of Clinical Medicine 10, 1962. doi: 10.3390/jcm10091962 | Unrelated |
|  | Hemila, H., and Chalker, E. (2021). Carrageenan nasal spray may double the rate of recovery from coronavirus and influenza virus infections: Re-analysis of randomized trial data. Pharmacology Research and Perspectives 9, e00810. doi: 10.1002/prp2.810 | Unrelated |
|  | Hoffmann, T., Bakhit, M., Krzyzaniak, N., Del Mar, C., Scott, A. M., and Glasziou, P. (2021). Soap versus sanitiser for preventing the transmission of acute respiratory infections in the community: A systematic review with meta-analysis and dose-response analysis. BMJ Open 11, e046175. doi: 10.1136/bmjopen-2020-046175 | Unrelated |
|  | Hyde, Z. (2021). COVID-19, children and schools: overlooked and at risk. Medical Journal of Australia 214, 190-191.e1. doi: 10.5694/mja2.50934 | Unrelated |
|  | Islam, N., Ebrahimzadeh, S., Salameh, J. P., Kazi, S., Fabiano, N., Treanor, L., Absi, M., Hallgrimson, Z., Leeflang, M. M. G., Hooft, L., van der Pol, C. B., Prager, R., Hare, S. S., Dennie, C., Spijker, R., Deeks, J. J., Dinnes, J., Jenniskens, K., Korevaar, D. A., Cohen, J. F., Van den Bruel, A., Takwoingi, Y., van de Wijgert, J., Damen, J. A. A. G., Wang, J., McInnes, M. D. F., Davenport, C., Emperador, D., Dittrich, S., Adriano, A., Beese, S., Dretzke, J., Ferrante di RuQano, L., Harris, I., Price, M., Taylor-Phillips, S., Stuyf, T., Domen, J., Horn, S., Yang, B., Langendam, M., Ochodo, E., Guleid, F., Holtman, G., Verbakel, J., Stegeman, I., Agarwal, R., Baldwin, S., Berhane, S., Herd, C., Kristunas, C., Quinn, L., and Scholefield, B. (2021). Thoracic imaging tests for the diagnosis of COVID-19. Cochrane Database of Systematic Reviews 2021, CD013639. doi: 10.1002/14651858.CD013639.pub4 | Unrelated |
|  | Joshi, N., Bassiony, S., Mathyalakan, A., and Sugai, T. (2021). Re-audit of cell free foetal DNA (cffDNA) screen to avoid administration of anti-D immunoglobulin in RHD-negative pregnant women with RHD-negative foetus. British Journal of Haematology 193, 14. doi: 10.1111/bjh.17490 | Unrelated |
|  | Joy, M., Malavika, B., Asirvatham, E. S., Sudarsanam, T. D., and Jeyaseelan, L. (2021). Is BCG associated with reduced incidence of COVID-19? A meta-regression of global data from 160 countries. Clinical Epidemiology and Global Health 9, 202-203. doi: 10.1016/j.cegh.2020.08.015 | Unrelated |
|  | Kong, Z., Zheng, J., Wu, J., Ou, J., Zhou, X., and Huang, H. (2021). Efficacy and safety of Xijiao Dihuang decoction in treating Henoch-Schonlein purpura: Study protocol for systematic review. Medicine (United States) 100, E28291. doi: 10.1097/MD.0000000000028291 | Unrelated |
|  | Lamrani, L., Manlhiot, C., Elias, M. D., Choueiter, N. F., Dionne, A., Harahsheh, A. S., Portman, M. A., McCrindle, B. W., and Dahdah, N. (2021). Kawasaki Disease Shock Syndrome vs Classical Kawasaki Disease: A Meta-analysis and Comparison With SARS-CoV-2 Multisystem Inflammatory Syndrome. Canadian Journal of Cardiology 37, 1619-1628. doi: 10.1016/j.cjca.2021.05.014 | Unrelated |
|  | Lane, J. C. E., Weaver, J., Kostka, K., Duarte-Salles, T., Abrahao, M. T. F., Alghoul, H., Alser, O., Alshammari, T. M., Areia, C., Biedermann, P., Banda, J. M., Burn, E., Casajust, P., Fister, K., Hardin, J., Hester, L., Hripcsak, G., Kaas-Hansen, B. S., Khosla, S., Kolovos, S., Lynch, K. E., Makadia, R., Mehta, P. P., Morales, D. R., Morgan-Stewart, H., Mosseveld, M., Newby, D., Nyberg, F., Ostropolets, A., Woong Park, R., Prats-Uribe, A., Rao, G. A., Reich, C., Rijnbeek, P., Sena, A. G., Shoaibi, A., Spotnitz, M., Subbian, V., Suchard, M. A., Vizcaya, D., Wen, H., Wilde, M. D., Xie, J., You, S. C., Zhang, L., Lovestone, S., Ryan, P., and Prieto-Alhambra, D. (2021). Risk of depression, suicide and psychosis with hydroxychloroquine treatment for rheumatoid arthritis: A multinational network cohort study. Rheumatology (United Kingdom) 60, 3222-3234. doi: 10.1093/rheumatology/keaa771 | Unrelated |
|  | Laurenzi, C. A., du Toit, S., Ameyan, W., Melendez-Torres, G. J., Kara, T., Brand, A., Chideya, Y., Abrahams, N., Bradshaw, M., Page, D. T., Ford, N., Sam-Agudu, N. A., Mark, D., Vitoria, M., Penazzato, M., Willis, N., Armstrong, A., and Skeen, S. (2021). Psychosocial interventions for improving engagement in care and health and behavioural outcomes for adolescents and young people living with HIV: a systematic review and meta-analysis. Journal of the International AIDS Society 24, e25741. doi: 10.1002/jia2.25741 | Unrelated |
|  | Li, P., Wang, Y., Peppelenbosch, M. P., Ma, Z., and Pan, Q. (2021). Systematically comparing COVID-19 with the 2009 influenza pandemic for hospitalized patients. International Journal of Infectious Diseases 102, 375-380. doi: 10.1016/j.ijid.2020.11.127 | Unrelated |
|  | Li, Y., Cao, L., Zhang, Z., Hou, L., Qin, Y., Hui, X., Li, J., Zhao, H., Cui, G., Cui, X., Li, R., Lin, Q., Li, X., and Yang, K. (2021). Reporting and methodological quality of COVID-19 systematic reviews needs to be improved: evidence mapping. Journal of Clinical Epidemiology 135, 17-28. doi: 10.1016/j.jclinepi.2021.02.021 | Unrelated |
|  | Lopes, J., Grimwood, A., Ngorima-Mabhena, N., Tiam, A., Tukei, B. B., Kasu, T., Mahachi, N., Mothibi, E., Tukei, V., Chasela, C., Lombard, C., and Fatti, G. (2021). Out-of-Facility Multimonth Dispensing of Antiretroviral Treatment: A Pooled Analysis Using Individual Patient Data From Cluster-Randomized Trials in Southern Africa. Journal of acquired immune deficiency syndromes (1999) 88, 477-486. doi: 10.1097/QAI.0000000000002797 | Unrelated |
|  | Lopes-Junior, L. C., Siqueira, P. C., and Maciel, E. L. N. (2021). School reopening and risks accelerating the COVID-19 pandemic: A systematic review and meta-analysis protocol. PLoS ONE 16, e0260189. doi: 10.1371/journal.pone.0260189 | Unrelated |
|  | Lopez-Leon, S., Wegman-Ostrosky, T., Perelman, C., Sepulveda, R., Rebolledo, P. A., Cuapio, A., and Villapol, S. (2021). More than 50 long-term effects of COVID-19: a systematic review and meta-analysis. Scientific reports 11, 16144. doi: 10.1038/s41598-021-95565-8 | Unrelated |
|  | Morgado-Carrasco, D., Ibaceta-Ayala, J., and Piquero-Casals, J. (2021). Hydroxychloroquine: An Essential Drug in Dermatology and Its Controversial Use in COVID-19. Actas dermo-sifiliograficas. doi: 10.1016/j.adengl.2021.11.020 | Unrelated |
|  | Mousa, A., Winskill, P., Watson, O. J., Ratmann, O., Monod, M., Ajelli, M., Diallo, A., Dodd, P. J., Grijalva, C. G., Kiti, M. C., Krishnan, A., Kumar, R., Kumar, S., Kwok, K. O., Lanata, C. F., de Waroux, O. L. P., Leung, K., Mahikul, W., Melegaro, A., Morrow, C. D., Mossong, J., Neal, E. F., Nokes, D. J., Pan-Ngum, W., Potter, G. E., Russell, F. M., Saha, S., Sugimoto, J. D., Wei, W. I., Wood, R. R., Wu, J., Zhang, J., Walker, P., and Whittaker, C. (2021). Social contact patterns and implications for infectious disease transmission - a systematic review and meta-analysis of contact surveys. eLife 10. doi: 10.7554/eLife.70294 | Unrelated |
|  | Mulder, I. A., Huntley, B., Di Mascio, D., Berghella, V., and Chauhan, S. P. (2021). 797 Adverse outcomes among individuals with and without SARS-CoV-2 infection: a systematic review and meta-analysis. American Journal of Obstetrics and Gynecology 224, S496-S497. doi: 10.1016/j.ajog.2020.12.820 | Unrelated |
|  | Mustafa Hellou, M., Gorska, A., Mazzaferri, F., Cremonini, E., Gentilotti, E., De Nardo, P., Poran, I., Leeflang, M. M., Tacconelli, E., and Paul, M. (2021). Nucleic acid amplification tests on respiratory samples for the diagnosis of coronavirus infections: a systematic review and meta-analysis. Clinical Microbiology and Infection 27, 341-351. doi: 10.1016/j.cmi.2020.11.002 | Unrelated |
|  | Ng, S. M., and Moore, H. S. (2021). Drug therapies for reducing gastric acidity in people with cystic fibrosis. Cochrane Database of Systematic Reviews 2021, CD003424. doi: 10.1002/14651858.CD003424.pub5 | Unrelated |
|  | Ong, T. G., Haider, A., Gottstein, R., and Nedungadi, S. (2021). Vertical transmission of covid 19. Archives of Disease in Childhood 106, A424. doi: 10.1136/archdischild-2021-rcpch.737 | Unrelated |
|  | Renosa, M. D. C., Landicho, J., Wachinger, J., Dalglish, S. L., Barnighausen, K., Barnighausen, T., and McMahon, S. A. (2021). Nudging toward vaccination: A systematic review. BMJ Global Health 6, e006237. doi: 10.1136/bmjgh-2021-006237 | Unrelated |
|  | Rogers, J. P., Chesney, E., Oliver, D., Begum, N., Saini, A., Wang, S., McGuire, P., Fusar-Poli, P., Lewis, G., and David, A. S. (2021). Suicide, self-harm and thoughts of suicide or self-harm in infectious disease epidemics: A systematic review and meta-analysis. Epidemiology and Psychiatric Sciences, e32. doi: 10.1017/S2045796021000214 | Unrelated |
|  | Schreiner, T. G., and Genes, T. M. (2021). Obesity and multiple sclerosis-a multifaceted association. Journal of Clinical Medicine 10, 2689. doi: 10.3390/jcm10122689 | Unrelated |
|  | Shah, K., Varna, V. P., Pandya, A., and Saxena, D. (2021). Low vitamin D levels and prognosis in a COVID-19 pediatric population: A systematic review. QJM 114, 447-453. doi: 10.1093/qjmed/hcab202 | Unrelated |
|  | Siddiquea, B. N., Shetty, A., Bhattacharya, O., Afroz, A., and Billah, B. (2021). Global epidemiology of COVID-19 knowledge, attitude and practice: A systematic review and meta-analysis. BMJ Open 11, e051447. doi: 10.1136/bmjopen-2021-051447 | Unrelated |
|  | Sikakulya, F. K., Ssebuufu, R., Soria, J., Kiyaka, S. M., Molen, S. F., and Kyamanywa, P. (2021). Surgery of COVID-19-infected Patients in Africa: A Scoping Review. Annals of African Surgery 18, 200-207. doi: 10.4314/AAS.V18I4.3 | Unrelated |
|  | Smith, L., Butler, L., Tully, M. A., Jacob, L., Barnett, Y., Lopez-Sanchez, G. F., Lopez-Bueno, R., Shin, J. I., McDermott, D., Pfeifer, B. A., Pizzol, D., and Koyanagi, A. (2021). Hand-washing practices among adolescents aged 12-15 years from 80 countries. International Journal of Environmental Research and Public Health 18, 1-15. doi: 10.3390/ijerph18010138 | Unrelated |
|  | Spielberger, B. D., Goerne, T., Geweniger, A., Henneke, P., and Elling, R. (2021). Intra-Household and Close-Contact SARS-CoV-2 Transmission Among Children - a Systematic Review. Frontiers in Pediatrics 9, 613292. doi: 10.3389/fped.2021.613292 | Unrelated |
|  | Stoicescu, E. R., Ciuca, I. M., Iacob, R., Iacob, E. R., Marc, M. S., Birsasteanu, F., Manolescu, D. L., and Iacob, D. (2021). Is lung ultrasound helpful in COVID-19 neonates? - A systematic review. Diagnostics 11, 2296. doi: 10.3390/diagnostics11122296 | Unrelated |
|  | Stolakis, K., Marneras, C., Tosounidis, T., and Panagiotopoulos, E. (2021). Investigating depression during the quarantine due to the pandemic in a sample of elderly people in Patras. Journal of Musculoskeletal Neuronal Interactions 21, 170-171**.** | Unrelated |
|  | Stuurman, A. L., Biccler, J., Carmona, A., Descamps, A., Diez-Domingo, J., Munoz Quiles, C., Nohynek, H., Rizzo, C., and Riera-Montes, M. (2021). Brand-specific influenza vaccine effectiveness estimates during 2019/20 season in Europe - Results from the DRIVE EU study platform. Vaccine. doi: 10.1016/j.vaccine.2021.05.059 | Unrelated |
|  | Takagi, H. (2021). Risk and protective factors of SARS-CoV-2 infection. Journal of medical virology 93, 649-651. doi: 10.1002/jmv.26427 | Unrelated |
|  | Tan, W., and Eseadi, C. (2021). School closures were over-weighted against the mitigation of COVID-19 transmission: A literature review on the impact of school closures in the United States. Medicine (United States) 100, E26709. doi: 10.1097/MD.0000000000026709 | Unrelated |
|  | Viner, R., Waddington, C., Mytton, O., Booy, R., Cruz, J., Ward, J., Ladhani, S., Panovska-Griffiths, J., Bonell, C., and Melendez-Torres, G. J. (2021). Transmission of SARS-CoV-2 by children and young people in households and schools: a meta-analysis of population-based and contact-tracing studies. medRxiv. doi: 10.1101/2021.12.14.21267713 | Unrelated |
|  | Wu, Q., Dudley, M. Z., Chen, X., Bai, X., Dong, K., Zhuang, T., Salmon, D., and Yu, H. (2021). Evaluation of the safety profile of COVID-19 vaccines: a rapid review. BMC medicine 19, 173. doi: 10.1186/s12916-021-02059-5 | Unrelated |
|  | Xiao, S., Qi, H., Ward, M. P., Wang, W., Zhang, J., Chen, Y., Bergquist, R., Tu, W., Shi, R., Hong, J., Su, Q., Zhao, Z., Ba, J., Qin, Y., and Zhang, Z. (2021). Meteorological conditions are heterogeneous factors for COVID-19 risk in China. Environmental Research 198, 111182. doi: 10.1016/j.envres.2021.111182 | Unrelated |
|  | Xun, Y., Yang, N., Li, Y., Si, W., Shi, Q., Wang, Z., Liu, X., Yu, X., Zhou, Q., Yang, M., and Chen, Y. (2021). Associations of hand washing frequency with the incidence of illness: A systematic review and meta-analysis. Annals of Translational Medicine 9, 395. doi: 10.21037/atm-20-6005 | Unrelated |
|  | Yokota, I., Sakurazawa, T., Sugita, J., Iwasaki, S., Yasuda, K., Yamashita, N., Fujisawa, S., Nishida, M., Konno, S., and Teshima, T. (2021). Performance of qualitative and quantitative antigen tests for SARS-CoV-2 using Saliva. Infectious Disease Reports 13, 742-747. doi: 10.3390/IDR13030069 | Unrelated |
|  | Zhou, B., Yuan, Y., Wang, S., Zhang, Z., Yang, M., Deng, X., and Niu, W. (2021). Risk profiles of severe illness in children with COVID-19: a meta-analysis of individual patients. Pediatric Research 90, 347-352. doi: 10.1038/s41390-021-01429-2 | Unrelated |
|  | Zhu, Y., Bloxham, C. J., Hulme, K. D., Sinclair, J. E., Tong, Z. W. M., Steele, L. E., Noye, E. C., Lu, J., Xia, Y., Chew, K. Y., Pickering, J., Gilks, C., Bowen, A. C., and Short, K. R. (2021). A Meta-analysis on the Role of Children in Severe Acute Respiratory Syndrome Coronavirus 2 in Household Transmission Clusters. Clinical Infectious Diseases 72, E1146-E1153. doi: 10.1093/cid/ciaa1825 | Unrelated |
|  | Zizza, A., Banchelli, F., Guido, M., Marotta, C., Di Gennaro, F., Mazzucco, W., Pistotti, V., and D'Amico, R. (2021). Efficacy and safety of human papillomavirus vaccination in HIV-infected patients: a systematic review and meta-analysis. Scientific reports 11, 4954. doi: 10.1038/s41598-021-83727-7 | Unrelated |
|  | Al Janabi, T., Petrillo, G., Chung, S., and Pino, M. (2022). Predictors of Vaccine Uptake among Migrants in the United States: A Rapid Systematic Review. Epidemiologia (Basel, Switzerland) 3, 465-481. doi: 10.3390/epidemiologia3040035 | Unrelated |
|  | Ali, H. A., Hartner, A.-M., Echeverria-Londono, S., Roth, J., Li, X., Abbas, K., Portnoy, A., Vynnycky, E., Woodruff, K., Ferguson, N. M., Toor, J., and Gaythorpe, K. A. (2022). Vaccine equity in low- and middle-income countries: a systematic review and meta-analysis. International journal for equity in health 21, 82. doi: 10.1186/s12939-022-01678-5 | Unrelated |
|  | Alimoradi, Z., Ohayon, M. M., Griffiths, M. D., Lin, C. Y., and Pakpour, A. H. (2022). Fear of COVID-19 and its association with mental health-related factors: Systematic review and meta-analysis. BJPsych Open 8, e73. doi: 10.1192/bjo.2022.26 | Unrelated |
|  | Ambrosino, P., Sanduzzi Zamparelli, S., Mosella, M., Formisano, R., Molino, A., Spedicato, G. A., Papa, A., Motta, A., Di Minno, M. N. D., and Maniscalco, M. (2022). Clinical assessment of endothelial function in convalescent COVID-19 patients: a meta-analysis with meta-regressions. Annals of Medicine 54, 3234-3249. doi: 10.1080/07853890.2022.2136403 | Unrelated |
|  | Canadian Neurological Sciences Federation 2022 Congress, CNSF. Canadian Journal of Neurological Sciences 49. | Unrelated |
|  | Critical Care Canada Forum 2021 Abstracts. Canadian Journal of Anesthesia 69. | Unrelated |
|  | 17th Congress of the Italian Society of Experimental Hematology. Haematologica 107. | Unrelated |
|  | Proceedings of the Canadian Society of Allergy and Clinical Immunology Annual Scientifc Meeting 2021. Allergy, Asthma and Clinical Immunology 18. | Unrelated |
|  | Variation in the COVID-19 infection-fatality ratio by age, time, and geography during the pre-vaccine era: a systematic analysis. Lancet (London, England) 399, 1469-1488. | Unrelated |
|  | Azh, N., Barzkar, F., Motamed-Gorji, N., Pourvali-Talatappeh, P., Moradi, Y., Vesal Azad, R., Ranjbar, M., and Baradaran, H. (2022). Nonsteroidal anti-inflammatory drugs in acute viral respiratory tract infections: An updated systematic review. Pharmacology Research and Perspectives 10, e00925. doi: 10.1002/prp2.925 | Unrelated |
|  | Barican, J. L., Yung, D., Schwartz, C., Zheng, Y., Georgiades, K., and Waddell, C. (2022). Prevalence of childhood mental disorders in high-income countries: a systematic review and meta-analysis to inform policymaking. Evidence-based mental health 25, 36-44. doi: 10.1136/ebmental-2021-300277 | Unrelated |
|  | Bersia, M., Koumantakis, E., Berchialla, P., Charrier, L., Ricotti, A., Grimaldi, P., Dalmasso, P., and Comoretto, R. I. (2022). Suicide spectrum among young people during the COVID-19 pandemic: A systematic review and meta-analysis. eClinicalMedicine 54, 101705. doi: 10.1016/j.eclinm.2022.101705 | Unrelated |
|  | Borchering, R. K., Mullany, L. C., Howerton, E., Chinazzi, M., Smith, C. P., Qin, M., Reich, N. G., Contamin, L., Levander, J., Kerr, J., Espino, J., Hochheiser, H., Lovett, K., Kinsey, M., Tallaksen, K., Wilson, S., Shin, L., Lemaitre, J. C., Hulse, J. D., Kaminsky, J., Lee, E. C., Davis, J. T., Mu, K., Xiong, X., Pastore y Piontti, A., Vespignani, A., Srivastava, A., Porebski, P., Venkatramanan, S., Adiga, A., Lewis, B., Klahn, B., Outten, J., Hurt, B., Chen, J., Mortveit, H., Wilson, A., Marathe, M., Hoops, S., Bhattacharya, P., Machi, D., Chen, S., Paul, R., Janies, D., Thill, J. C., Galanti, M., Yamana, T., Pei, S., Shaman, J., Espana, G., Cavany, S., Moore, S., Perkins, A., Healy, J. M., Slayton, R. B., Johansson, M. A., Biggerstaff, M., Shea, K., Truelove, S. A., Runge, M. C., Viboud, C., and Lessler, J. (2022). Impact of SARS-CoV-2 vaccination of children ages 5-11 years on COVID-19 disease burden and resilience to new variants in the United States, November 2021-March 2022: a multi-model study. medRxiv. doi: 10.1101/2022.03.08.22271905 | Unrelated |
|  | Bortolani, S., Brusa, C., Rolle, E., Monforte, M., De Arcangelis, V., Ricci, E., Mongini, T. E., and Tasca, G. (2022). Technology outcome measures in neuromuscular disorders: A systematic review. European Journal of Neurology 29, 1266-1278. doi: 10.1111/ene.15235 | Unrelated |
|  | Bots, S. H., Riera-Arnau, J., Belitser, S. V., Messina, D., Aragon, M., Alsina, E., Douglas, I. J., Duran, C. E., Garcia-Poza, P., Gini, R., Herings, R. M. C., Huerta, C., Sisay, M. M., Martin-Perez, M., Martin, I., Overbeek, J. A., Paoletti, O., Palleja-Millan, M., Schultze, A., Souverein, P., Swart, K. M. A., Villalobos, F., Klungel, O. H., and Sturkenboom, M. C. J. M. (2022). Myocarditis and pericarditis associated with SARS-CoV-2 vaccines: A population-based descriptive cohort and a nested self-controlled risk interval study using electronic health care data from four European countries. Frontiers in Pharmacology 13, 1038043. doi: 10.3389/fphar.2022.1038043 | Unrelated |
|  | Braun, A. S., Feil, K., Reiser, E., Weiss, G., Von Steuben, T., Pinggera, G. M., Kohn, F. M., and Toth, B. (2022). Corona and Reproduction, or Why the Corona Vaccination Does Not Result in Infertility. Geburtshilfe und Frauenheilkunde 82, 490-500. doi: 10.1055/a-1750-9284 | Unrelated |
|  | Caini, S., Martinoli, C., Vecchia, C. L., Raimondi, S., Bellerba, F., D'Ecclesiis, O., Sasso, C., Basso, A., Cammarata, G., and Gandini, S. (2022). SARS-CoV-2 Circulation in the School Setting: A Systematic Review and Meta-Analysis. International Journal of Environmental Research and Public Health 19, 5384. doi: 10.3390/ijerph19095384 | Unrelated |
|  | Caranti, A., Bianchini, C., Corazzi, V., Pelucchi, S., and Ciorba, A. (2022). Tapia's Syndrome: keep it in mind! Minerva Anestesiologica 88, 293-299. doi: 10.23736/S0375-9393.21.16037-7 | Unrelated |
|  | Charron, C. M., and Gorey, K. M. (2022). Virtual versus Face-to-Face Cognitive Behavioral Treatment of Depression: Meta-Analytic Test of a Noninferiority Hypothesis and Men's Mental Health Inequities. Depression research and treatment 2022, 2972219. doi: 10.1155/2022/2972219 | Unrelated |
|  | Chaudhari, H. G., Patil, R. U., Jathar, P. N., and Jain, C. A. (2022). A systematic review of randomized controlled trials on survival rate of atraumatic restorative treatment compared with conventional treatment on primary dentition. Journal of the Indian Society of Pedodontics and Preventive Dentistry 40, 112-117. doi: 10.1155/2022/2972219 | Unrelated |
|  | Chen, C.-C., Chen, S.-Y., Fang, S.-B., Lu, S.-C., Bai, C.-H., and Wang, Y.-H. (2022). Diagnostic accuracy of SARS-CoV-2 antigen test in the pediatric population: A systematic review and meta-analysis. Pediatrics and neonatology. doi: 10.1016/j.pedneo.2022.07.012. doi: 10.1016/j.pedneo.2022.07.012 | Unrelated |
|  | Chen, C. C., Hsiao, K. Y., Bai, C. H., and Wang, Y. H. (2022). Investigation of the diagnostic performance of the SARS-CoV-2 saliva antigen test: A meta-analysis. Journal of Microbiology, Immunology and Infection 55, 1084-1093. doi: 10.1016/j.jmii.2022.07.003 | Unrelated |
|  | Chen, C. C., Yang, Y. P., Tsai, H. L., and Tung, T. H. (2022). Effects of Tocilizumab on Adults With COVID-19 Pneumonia: A Meta-Analysis. Frontiers in Medicine 9, 838904. doi: 10.3389/fmed.2022.838904 | Unrelated |
|  | Chen, F., He, Y., and Shi, Y. (2022). Parents' and Guardians' Willingness to Vaccinate Their Children against COVID-19: A Systematic Review and Meta-Analysis. Vaccines 10, 179. doi: 10.3390/vaccines10020179 | Unrelated |
|  | Chen, F., Tian, Y., Zhang, L., and Shi, Y. (2022). The role of children in household transmission of COVID-19: a systematic review and meta-analysis. International Journal of Infectious Diseases 122, 266-275. doi: 10.1016/j.ijid.2022.05.016 | Unrelated |
|  | Chen, Z., Luo, J., Li, S., Xu, P., Zeng, L., Yu, Q., and Zhang, L. (2022). Characteristics of Living Systematic Review for COVID-19. Clinical Epidemiology 14, 925-935. doi: 10.2147/CLEP.S367339 | Unrelated |
|  | Cordero, A., Cazorla, D., Escribano, D., Quintanilla, M. A., Lopez-Ayala, J. M., Berbel, P. P., and Bertomeu-Gonzalez, V. (2022). Myocarditis after RNA-based vaccines for coronavirus. International journal of cardiology 353, 131-134. doi: 10.1016/j.ijcard.2022.01.037 | Unrelated |
|  | Cortes-Albornoz, M. C., Ramirez-Guerrero, S., Rojas-Carabali, W., De-La-Torre, A., and Talero-Gutierrez, C. (2022). Effects of remote learning during the COVID-19 lockdown on children's visual health: a systematic review. BMJ Open 12, e062388. doi: 10.1136/bmjopen-2022-062388 | Unrelated |
|  | Craiu, D., Rener Primec, Z., Lagae, L., Vigevano, F., Trinka, E., Specchio, N., Bakhtadze, S., Cazacu, C., Golli, T., and Zuberi, S. M. (2022). Vaccination and childhood epilepsies. European Journal of Paediatric Neurology 36, 57-68. doi: 10.1016/j.ejpn.2021.11.014 | Unrelated |
|  | Crist, K. D., Schor, S., Levochkina, O., Borba, C., and McMahon, J. (2022). (204) The Role of Telehealth in an Obstetric-Psychiatry Clinic: Patient Perceptions. Journal of the Academy of Consultation-Liaison Psychiatry 63, S212. doi: 10.1016/j.jaclp.2022.10.206 | Unrelated |
|  | Crivelli, L., Palmer, K., Calandri, I., Guekht, A., Beghi, E., Carroll, W., Frontera, J., Garcia-Azorin, D., Westenberg, E., Winkler, A. S., Mangialasche, F., Allegri, R. F., and Kivipelto, M. (2022). Changes in cognitive functioning after COVID-19: A systematic review and meta-analysis. Alzheimer's & dementia: the journal of the Alzheimer's Association 18, 1047-1066. doi: 10.1002/alz.12644 | Unrelated |
|  | De Simone, S., Franco, M., Servillo, G., and Vargas, M. (2022). Implementations and strategies of telehealth during COVID-19 outbreak: a systematic review. BMC health services research 22, 833. doi: 10.1186/s12913-022-08235-4 | Unrelated |
|  | Deng, X., He, M., Zhang, J., Huang, J., Luo, M., Zhang, Z., and Niu, W. (2022). SARS-CoV-Related Pandemic Outbreaks and Mental Disorder Risk. Journal of Nervous and Mental Disease 210, 900-911. doi: 10.1097/NMD.0000000000001543 | Unrelated |
|  | Diaz, L. A., Garcia-Salum, T., Fuentes-Lopez, E., Reyes, D., Ortiz, J., Chahuan, J., Levican, J., Almonacid, L. I., Valenzuela, G. H., Serrano, E., Budnik, S., Gandara, V., Gallardo, A., Seydewitz, M. F., Ferres, M., Cofre, C., Alvarez, M., Pavez, C., Candia, R., Monrroy, H., Espino, A., Rada, G., Ortiz, L., Valderrama, S., Salinas, E., Toro, A., Ortega, M., Pizarro, M., Medina, R. A., and Riquelme, A. (2022). High prevalence of SARS-CoV-2 detection and prolonged viral shedding in stools: A systematic review and cohort study. Gastroenterologia y Hepatologia 45, 593-604. doi: 10.1016/j.gastrohep.2021.12.009 | Unrelated |
|  | Farooq, H., Aemaz Ur Rehman, M., Asmar, A., Asif, S., Mushtaq, A., and Qureshi, M. A. (2022). The pathogenesis of COVID-19-induced IgA nephropathy and IgA vasculitis: A systematic review. Journal of Taibah University Medical Sciences 17, 1-13. doi: 10.1016/j.jtumed.2021.08.012 | Unrelated |
|  | Fayon, M. (2022). Cigarette Smoke Exposure and Lung Health. Pediatric Pulmonology 57, S36-S38. doi: 10.1002/ppul.25961 | Unrelated |
|  | Fazel, N., Shahrooyan, S., and Shahrouyan, S. (2022). Pregnancy Outcomes with Coronavirus Infection (COVID-19). Acta Facultatis Medicae Naissensis 39, 117-140. doi: 10.5937/afmnai39-32704 | Unrelated |
|  | Flacco, M. E., Acuti Martellucci, C., Baccolini, V., De Vito, C., Renzi, E., Villari, P., and Manzoli, L. (2022). Risk of reinfection and disease after SARS-CoV-2 primary infection: Meta-analysis. European Journal of Clinical Investigation 52, e13845. doi: 10.1111/eci.13845 | Unrelated |
|  | Fonfria, E. S., Vigo, M. I., Garcia-Garcia, D., Herrador, Z., Navarro, M., and Bordehore, C. (2022). Scoping review and meta-analysis of COVID-19 epidemiological parameters for modeling from early Asian studies. medRxiv. doi: 10.1101/2022.10.23.22281408 | Unrelated |
|  | Galanis, P., Vraka, I., Siskou, O., Konstantakopoulou, O., Katsiroumpa, A., and Kaitelidou, D. (2022). Willingness, refusal and influential factors of parents to vaccinate their children against the COVID-19: A systematic review and meta-analysis. Preventive Medicine 157, 106994. doi: 10.1016/j.ypmed.2022.106994 | Unrelated |
|  | Gibb, F. (2022). The year in benign thyroid disease research. Thyroid Research 15. doi: 10.1186/s13044-022-00129-1 | Unrelated |
|  | Gonidakis, F. (2022). Eating disorders in the era of the COVID-19 pandemic. Psychiatrike 33, 267-270. doi: 10.22365/jpsych.2022.096 | Unrelated |
|  | Grana, C., Ghosn, L., Evrenoglou, T., Jarde, A., Minozzi, S., Bergman, H., Buckley, B. S., Probyn, K., Villanueva, G., Henschke, N., Bonnet, H., Assi, R., Menon, S., Marti, M., Devane, D., Mallon, P., Lelievre, J. D., Askie, L. M., Kredo, T., Ferrand, G., Davidson, M., Riveros, C., Tovey, D., Meerpohl, J. J., Grasselli, G., Rada, G., Hrobjartsson, A., Ravaud, P., Chaimani, A., and Boutron, I. (2022). Efficacy and safety of COVID-19 vaccines. Cochrane Database of Systematic Reviews 2022, CD015477. doi: 10.1002/14651858.CD015477 | Unrelated |
|  | Gregorczyk, M., and Roskal-Walek, J. (2022). [Ocular symptoms in SARS-CoV-2 infection]. Objawy oczne w zakazeniu SARS-CoV-2. 50, 86-93. | Unrelated |
|  | Grossi, U., Gallo, G., Ortenzi, M., Piccino, M., Salimian, N., Guerrieri, M., Sammarco, G., Felice, C., Santoro, G. A., Di Saverio, S., Di Tanna, G. L., and Zanus, G. (2022). Changes in hospital admissions and complications of acute appendicitis during the COVID-19 pandemic: A systematic review and meta-analysis. Health sciences review (Oxford, England) 3, 100021. doi: 10.1016/j.hsr.2022.100021 | Unrelated |
|  | Haas, J. W., Bender, F. L., Ballou, S., Kelley, J. M., Wilhelm, M., Miller, F. G., Rief, W., and Kaptchuk, T. J. (2022). Frequency of Adverse Events in the Placebo Arms of COVID-19 Vaccine Trials: A Systematic Review and Meta-analysis. JAMA Network Open, e2143955. doi: 10.1001/jamanetworkopen.2021.43955 | Unrelated |
|  | Hagrass, A. I., Almadhoon, H. W., Al-kafarna, M., Almaghary, B. K., Nourelden, A. Z., Fathallah, A. H., Hasan, M. T., Mohammed, Y. A., Al-Nabahin, A. O., Wafi, D. S., Ismail, I. O., Hamam, Y. A., Sayad, R., Hamouda, M., Zaazouee, M. S., and Ragab, K. M. (2022). Maternal and neonatal safety outcomes after SAR-CoV-2 vaccination during pregnancy: a systematic review and meta-analysis. BMC Pregnancy and Childbirth 22, 581. doi: 10.1186/s12884-022-04884-9 | Unrelated |
|  | Hameed, I., Khan, M. O., Nusrat, K., Mahmood, S., Nashit, M., Malik, S., Siddiqui, O. M., Samad, S. A., Marsia, S., Usman, M. S., and Siddiqi, T. J. (2022). Is it safe and effective to administer COVID-19 vaccines during pregnancy? A systematic review and meta-analysis. American Journal of Infection Control. doi: 10.1016/j.ajic.2022.08.014 | Unrelated |
|  | Hammour, K. A., Farha, R. A., Manaseer, Q., Dawoud, T., and Hammour, W. A. (2022). Is COVID-19 multisystem inflammatory syndrome a new variant of Kawasaki Disease? Archives of Rheumatology 37, 230-244. doi: 10.46497/ArchRheumatol.2022.9086 | Unrelated |
|  | Hanson, S. W., Abbafati, C., Aerts, J. G., Al-Aly, Z., Ashbaugh, C., Ballouz, T., Blyuss, O., Bobkova, P., Bonsel, G., Borzakova, S., Buonsenso, D., Butnaru, D., Carter, A., Chu, H., De Rose, C., Diab, M. M., Ekbom, E., El Tantawi, M., Fomin, V., Frithiof, R., Gamirova, A., Glybochko, P. V., Haagsma, J. A., Javanmard, S. H., Hamilton, E. B., Harris, G., Heijenbrok-Kal, M. H., Helbok, R., Hellemons, M. E., Hillus, D., Huijts, S. M., Hultstrom, M., Jassat, W., Kurth, F., Ing-Marie, L., Lipcsey, M., Liu, C., Loflin, C. D., Malinovschi, A., Mao, W., Mazankova, L., McCulloch, D., Menges, D., Mohammadifard, N., Munblit, D., Nekliudov, N. A., Ogbuoji, O., Osmanov, I. M., Penalvo, J. L., Skaalum Petersen, M., Puhan, M. A., Rahman, M., Rass, V., Reinig, N., Ribbers, G. M., Ricchiuto, A., Rubertsson, S., Samitova, E., Sarrafzadegan, N., Shikhaleva, A., Simpson, K. E., Sinatti, D., Soriano, J. B., Spiridonova, E., Steinbeis, F., Svistunov, A. A., Valentini, P., van de Water, B. J., van den Berg-Emons, R., Wallin, E., Witzenrath, M., Wu, Y., Xu, H., Zoller, T., Adolph, C., Albright, J., Amlag, J. O., Aravkin, A. Y., Bang-Jensen, B. L., Bisignano, C., Castellano, R., Castro, E., Chakrabarti, S., Collins, J. K., Dai, X., Daoud, F., Dapper, C., Deen, A., Duncan, B. B., Erickson, M., Ewald, S. B., Ferrari, A. J., Flaxman, A. D., Fullman, N., Gamkrelidze, A., Giles, J. R., Guo, G., Hay, S. I., He, J., Helak, M., et al. (2022). A global systematic analysis of the occurrence, severity, and recovery pattern of long COVID in 2020 and 2021. medRxiv. doi: 10.1101/2022.05.26.22275532 | Unrelated |
|  | Hartman-Munick, S. M., Lin, J. A., Milliren, C. E., Braverman, P. K., Fisher, M. M., Jary, J. M., Lemly, D. C., Ornstein, R. M., Roche, A., Rome, E. S., Rosen, E. L., Thew, M., Vo, M., Voss, M., Woods, E. R., Forman, S. F., and Richmond, T. K. (2022). 12. The Impact of the COVID-19 Pandemic on Adolescent/Young Adult Eating Disorder Patient Visits: Data from the National Eating Disorder Quality Improvement Collaborative. Journal of Adolescent Health 70, S7. doi: 10.1016/j.jadohealth.2022.01.016 | Unrelated |
|  | Hartwell, M., Lin, V., Gatewood, A., Sajjadi, N. B., Garrett, M., Reddy, A. K., Greiner, B., and Price, J. (2022). Health disparities, COVID-19, and maternal and childbirth outcomes: a meta-epidemiological study of equity reporting in systematic reviews. Journal of Maternal-Fetal and Neonatal Medicine 35, 9622-9630. doi: 10.1080/14767058.2022.2049750 | Unrelated |
|  | Hawco, S., Rolnik, D. L., Woolner, A., Cameron, N. J., Wyness, V., Mol, B. W., and Black, M. (2022). The impact of mitigation measures on perinatal outcomes during the first nine months of the COVID-19 pandemic: A systematic review with meta-analysis. European journal of obstetrics, gynecology, and reproductive biology 274, 117-127. doi: 10.1016/j.ejogrb.2022.05.007 | Unrelated |
|  | Hviid, A., Nieminen, T. A., Pihlstrom, N., Gunnes, N., Dahl, J., Karlstad, O., Gulseth, H. L., Sundstrom, A., Husby, A., Hansen, J. V., Ljung, R., and Hovi, P. (2022). Booster Vaccination with SARS-CoV-2 mRNA Vaccines and Myocarditis Risk in Adolescents and Young Adults: A Nordic Cohort Study of 8.9 Million Residents. medRxiv. doi: 10.1101/2022.12.16.22283603 | Unrelated |
|  | Jaganath, D. (2022). Advances in New Diagnostics for Pulmonary TB. Pediatric Pulmonology 57, S48-S49. doi: 10.1002/ppul.25961 | Unrelated |
|  | Kaggwa, M. M., Najjuka, S. M., Bongomin, F., Mamun, M. A., and Griffiths, M. D. (2022). Prevalence of depression in Uganda: A systematic review and meta-analysis. PLoS ONE 17, e0276552. doi: 10.1371/journal.pone.0276552 | Unrelated |
|  | Kelleni, M. T. (2022). NSAIDs and Kelleni's protocol as potential early COVID-19 treatment game changer: could it be the final countdown? Inflammopharmacology 30, 343-348. doi: 10.1007/s10787-021-00896-7 | Unrelated |
|  | Khambaty, M., Silbert, R. E., Devalapalli, A. P., Kashiwagi, D. T., Regan, D. W., Sundsted, K. K., and Mauck, K. F. (2022). Practice-Changing Updates in Perioperative Medicine Literature 2020-2021: A Systematic Review. The American journal of medicine 135, 1306-1314.e1. doi: 10.1016/j.amjmed.2022.06.003 | Unrelated |
|  | Kumar, J., Meena, J., Yadav, A., and Kumar, P. (2022). SARS-CoV-2 detection in human milk: a systematic review. Journal of Maternal-Fetal and Neonatal Medicine 35, 5456-5463. doi: 10.1080/14767058.2021.1882984 | Unrelated |
|  | Kumaran, M., Akande, O., Khalil, H., Lawson, H., Sheikh, J., Allotey, J., Zamora, J., Thangaratinam, S., Chatterjee, S. R., and Kew, T. (2022). Mother-to- child transmission of SARS-CoV- 2 and rates of neonatal positivity. BJOG: An International Journal of Obstetrics and Gynaecology 129, 149. doi: 10.1111/1471-0528.18_17178 | Unrelated |
|  | Lai, K. L., Hu, F. C., Wen, F. Y., and Chen, J. J. (2022). Lymphocyte count is a universal predictor of health outcomes in COVID-19 patients before mass vaccination: A meta-analytical study. Journal of global health 12, 05041. doi: 10.7189/jogh.12.05041 | Unrelated |
|  | Lee, E. K., Donley, G., Ciesielski, T. H., Gill, I., Yamoah, O., Roche, A., Martinez, R., and Freedman, D. A. (2022). Health outcomes in redlined versus non-redlined neighborhoods: A systematic review and meta-analysis. Social Science and Medicine 294, 114696. doi: 10.1016/j.socscimed.2021.114696 | Unrelated |
|  | Lewis, H. C., Ware, H., Whelan, M., Subissi, L., Li, Z., Ma, X., Nardone, A., Valenciano, M., Cheng, B., Noel, K., Cao, C., Yanes-Lane, M., Herring, B. L., Talisuna, A., Ngoy, N., Balde, T., Clifton, D., Van Kerkhove, M. D., Buckeridge, D., Bobrovitz, N., Okeibunor, J., Arora, R. K., and Bergeri, I. (2022). SARS-CoV-2 infection in Africa: A systematic review and meta-analysis of standardised seroprevalence studies, from January 2020 to December 2021. BMJ Global Health 7, e008793. doi: 10.1136/bmjgh-2022-008793 | Unrelated |
|  | Li, L., Zhang, Q., Zhu, L., Zeng, G., Huang, H., Zhuge, J., Kuang, X., Yang, S., Yang, D., Chen, Z., Gan, Y., Lu, Z., and Wu, C. (2022). Screen time and depression risk: A meta-analysis of cohort studies. Frontiers in Psychiatry 13, 1058572. doi: 10.3389/fpsyt.2022.1058572 | Unrelated |
|  | Li, M., Xu, L., Tan, C. S., Lanca, C., Foo, L. L., Sabanayagam, C., and Saw, S. M. (2022). Systematic Review and Meta-Analysis on the Impact of COVID-19 Pandemic-Related Lifestyle on Myopia. Asia-Pacific journal of ophthalmology (Philadelphia, Pa.) 11, 470-480. doi: 10.1097/APO.0000000000000559 | Unrelated |
|  | Li, X., Burn, E., Duarte-Salles, T., Yin, C., Reich, C., Delmestri, A., Verhamme, K., Rijnbeek, P., Suchard, M. A., Li, K., Mosseveld, M., John, L. H., Mayer, M.-A., Ramirez-Anguita, J.-M., Cohet, C., Strauss, V., and Prieto-Alhambra, D. (2022). Comparative risk of thrombosis with thrombocytopenia syndrome or thromboembolic events associated with different covid-19 vaccines: international network cohort study from five European countries and the US. BMJ (Clinical research ed.) 379, e071594. doi: 10.1136/bmj-2022-071594 | Unrelated |
|  | Liang, H., Zhang, M., Chen, M., Lin, T. P. H., Lai, M., and Chen, H. (2022). Ocular Trauma During COVID-19 Pandemic: A Systematic Review and Meta-analysis. Asia-Pacific journal of ophthalmology (Philadelphia, Pa.) 11, 481-487. doi: 10.1097/APO.0000000000000539 | Unrelated |
|  | Liang, Y., Sun, L., and Tan, X. (2022). Mental Health Research During the COVID-19 Pandemic: Focuses and Trends. Frontiers in public health 10, 895121. doi: 10.3389/fpubh.2022.895121 | Unrelated |
|  | Ling, R. R., Ramanathan, K., Tan, F. L., Tai, B. C., Somani, J., Fisher, D., and MacLaren, G. (2022). Myopericarditis following COVID-19 vaccination and non-COVID-19 vaccination: a systematic review and meta-analysis. The Lancet Respiratory Medicine 10, 679-688. doi: 10.1016/S2213-2600%2822%2900059-5 | Unrelated |
|  | Lippi, G., Henry, B. M., and Plebani, M. (2022). LumiraDX SARS-CoV-2 Antigen Test for Diagnosing Acute SARS-CoV-2 Infection: Critical Literature Review and Meta-Analysis. Diagnostics 12, 947. doi: 10.3390/diagnostics12040947 | Unrelated |
|  | Lott, A., Roberts, T., and Carter, C. W. (2022). Mask Use for Athletes: A Systematic Review of Safety and Performance Outcomes. Sports health 14, 632-647. doi: 10.1177/19417381221111395 | Unrelated |
|  | Mahroum, N., and Shoenfeld, Y. (2022). Classic Pityriasis Rosea. Israel Medical Association Journal 24, 549. | Unrelated |
|  | Manothummetha, K., Chuleerarux, N., Sanguankeo, A., Kates, O. S., Hirankarn, N., Thongkam, A., Dioverti-Prono, M. V., Torvorapanit, P., Langsiri, N., Worasilchai, N., Moonla, C., Plongla, R., Garneau, W. M., Chindamporn, A., Nissaisorakarn, P., Thaniyavarn, T., Nematollahi, S., and Permpalung, N. (2022). Immunogenicity and Risk Factors Associated With Poor Humoral Immune Response of SARS-CoV-2 Vaccines in Recipients of Solid Organ Transplant: A Systematic Review and Meta-Analysis. JAMA network open 5, e226822. doi: 10.1001/jamanetworkopen.2022.6822 | Unrelated |
|  | Mehta, A., Suman, P., and Garg, S. (2022). A SYSTEMATIC REVIEW AND META ANALYSIS OF THE EVIDENCE FROM THE PUBLISHED CASE REPORTS AND VACCINE ADVERSE EVENTS REPORTING SYSTEMS FOR THE MYOCARDITIS FOLLOWING COVID-19 VACCINATION. Journal of the American College of Cardiology 79, 2065. doi: 10.1016/S0735-1097%2822%2903056-X | Unrelated |
|  | Michelen, M., Sigfrid, L., Kartsonaki, C., Shemilt, I., Hastie, C., O'Hara, M. E., Suett, J. C., Stelson, E. A., Bugaeva, P., Dahmash, D. T., Rigby, I., Munblit, D., Harriss, E., Burls, A., Cheng, V., Scott, J. T., Carson, G., Olliaro, P., and Stavropoulou, C. (2022). Characterising Long Covid: a living systematic review update with controlled studies. medRxiv. doi: 10.1016/S0735-1097%2822%2903056-X | Unrelated |
|  | Miraglia del Giudice, M., Indolfi, C., Dinardo, G., Decimo, F., Decimo, A., and Klain, A. (2022). Vitamin D status can affect COVID-19 outcomes also in pediatric population. PharmaNutrition 22, 100319. doi: 10.1016/j.phanu.2022.100319 | Unrelated |
|  | Morgado-Carrasco, D., Ibaceta-Ayala, J., and Piquero-Casals, J. (2022). [Hydroxychloroquine: An Essential Drug in Dermatology and Its Controversial Use in COVID-19]. La hidroxicloroquina como farmaco fundamental en dermatologia y su papel controvertido en la COVID-19. 113, 166-175. doi: 10.1016/j.ad.2021.07.005 | Unrelated |
|  | Ningtiar, H. W., Heryawan, R., Pattayasuci, N. R., Purnama, Y., and Aditya, F. (2022). SYSTEMATIC REVIEW AND META-ANALYSIS ON THE INCIDENCE OF SHOCK AND THE USAGE OF VASOPRESSOR SUPPORT IN SEVERE COVID-19 AND MULTISYSTEM INFLAMMATORY SYNDROME IN CHILDREN. Pediatric Critical Care Medicine 23. doi: 10.1097/01.pcc.0000901348.95641.c0 | Unrelated |
|  | Nourmohammadi, H., Dehkordi, A. H., Adibi, A., Amin Hashemipour, S. M., Abdan, M., Fakhri, M., Abdan, Z., and Sarokhani, D. (2022). Seroprevalence of COVID-19 in Blood Donors: A Systematic Review and Meta-Analysis. Advances in Virology 2022, 9342680. doi: 10.1155/2022/9342680 | Unrelated |
|  | Otunla, A., Rees, K., Dennison, P., Hobbs, R., Suklan, J., Schofield, E., Gunnell, J., Mighiu, A., and Hartmann-Boyce, J. (2022). Risks of infection, hospital and ICU admission, and death from COVID-19 in people with asthma: systematic review and meta-analyses. BMJ evidence-based medicine 27, 263-273. doi: 10.1136/bmjebm-2021-111788 | Unrelated |
|  | Oza, P., Umbarkar, R., Goyal, V., and Shukla, P. (2022). Retrospective Analysis of Arterial Carbon Dioxide Level and Arterial pH Level at the Time of Initiation of Respiratory ECMO and Outcome. Journal of Cardiac Critical Care 6, 103-107. doi: 10.1055/s-0042-1757395 | Unrelated |
|  | Pata, D., Buonsenso, D., and Valentini, P. (2022). Comparison of the Clinical and Laboratory Features of COVID and Influenza in Children. Mediterranean Journal of Hematology and Infectious Diseases 14, e2022065. doi: 10.4084/MJHID.2022.065 | Unrelated |
|  | Ramirez-Soto, M. C., Ortega-Caceres, G., and Arroyo-Hernandez, H. (2022). Excess all-cause deaths stratified by sex and age in Peru: A time series analysis during the COVID-19 pandemic. BMJ Open 12, e057056. doi: 10.1136/bmjopen-2021-057056 | Unrelated |
|  | Ramirez-Suarez, K. I., Miranda-Schaeubinger, M., Rapp, J. B., Sodhi, K. S., Saul, D., and Andronikou, S. (2022). Publication timeline of chest imaging reporting in children with coronavirus disease 2019 (COVID-19): a systematic review spanning 2020. Pediatric Radiology 52, 1998-2008. doi: 10.1007/s00247-022-05466-9 | Unrelated |
|  | Redaelli, S., Magliocca, A., Malhotra, R., Ristagno, G., Citerio, G., Bellani, G., Berra, L., and Rezoagli, E. (2022). Nitric oxide: Clinical applications in critically ill patients. Nitric oxide: biology and chemistry 121, 20-33. doi: 10.1016/j.niox.2022.01.007 | Unrelated |
|  | Rouhani, S., and Flaherman, V. (2022). DELAYED NEURODEVELOPMENTAL ALTERATIONS IN INFANTS EXPOSED TO ZIKA VIRUS IN UTERO. Irish Journal of Medical Science 191, S41. doi: 10.1007/s11845-022-02939-6 | Unrelated |
|  | Shi, A., Tang, X., Xia, P., Hao, M., Shu, Y., Nakanishi, H., Smayra, K., Farzad, A., Hu, K., Liu, Q., Pan, S., Dixon, R. A. F., Wu, Y., Cai, P., Yu, P., and Li, P. (2022). Cardiac Arrhythmia after COVID-19 Vaccination versus Non-COVID-19 Vaccination: A Systematic Review and Meta-Analysis. medRxiv. doi: 10.1101/2022.11.21.22282554 | Unrelated |
|  | Wang, Z., Chen, S., and Fang, Y. (2022). Parental Willingness and Associated Factors of Pediatric Vaccination in the Era of COVID-19 Pandemic: A Systematic Review and Meta-Analysis. Vaccines 10, 1453. doi: 10.3390/vaccines10091453 | Unrelated |
|  | Wang, Z., Zhao, S., Tang, Y., Wang, Z., Shi, Q., Dang, X., Gan, L., Peng, S., Li, W., Zhou, Q., Li, Q., Mafiana, J. J., Cortes, R. G., Luo, Z., Liu, E., and Chen, Y. (2022). Potentially effective drugs for the treatment of COVID-19 or MIS-C in children: a systematic review. European journal of pediatrics 181, 2135-2146. doi: 10.1007/s00431-022-04388-w | Unrelated |
|  | Ye, Z., Chen, H., Liu, X., and Wei, G. (2022). Delayed Care Seeking and Outcomes of Testicular Torsion among Children during the COVID-19 Pandemic: A Systematic Review and Meta-Analysis. European Journal of Pediatric Surgery. doi: 10.1055/s-0042-1758153 | Unrelated |
|  | Zhao, S., Tang, Y., Wang, Z., Shi, Q., Dang, X., Gan, L., Peng, S., Li, W., Zhou, Q., Li, Q., Mafiana, J. J., Cortes, R. G., Luo, Z., Liu, E., and Chen, Y. (2022). Potentially effective drugs for the treatment of COVID-19 or MIS-C in children: a systematic review. European Journal of Pediatrics 181, 2135-2146. doi: 10.1007/s00431-022-04388-w | Unrelated |
|  | Cohen, M. A., Edelman, A., Paynter, R., and Henderson, J. T. (2023). Risk of thromboembolism in patients with COVID-19 who are using hormonal contraception. Cochrane Database of Systematic Reviews 2023, CD014908. doi: 10.1002/14651858.CD014908.pub2 | Unrelated |
|  | Jefferson, T., Dooley, L., Ferroni, E., Al-Ansary, L. A., van Driel, M. L., Bawazeer, G. A., Jones, M. A., Hoffmann, T. C., Clark, J., Beller, E. M., and et al. (2023). Physical interventions to interrupt or reduce the spread of respiratory viruses. Cochrane Database of Systematic Reviews. doi: 10.1002/14651858.CD006207.pub6 | Unrelated |
|  | Kulkarni, S., Thampi, V., Deshmukh, D., Gadhari, M., Chandrasekar, R., and Phadke, M. (2023). Trends in Urban Immunization Coverage in India: A Meta-Analysis and Meta-Regression. Indian Journal of Pediatrics 90, 38-48. doi: 10.1007/s12098-021-03843-0 | Unrelated |
|  | Lagathu, G., Grolhier, C., Besombes, J., Maillard, A., Comacle, P., Pronier, C., and Thibault, V. (2023). Using Discarded Facial Tissues to Monitor and Diagnose Viral Respiratory Infections. Emerging infectious diseases 29. doi: 10.3201/eid2903.221416 | Unrelated |
|  | Sugarman, D. E., and Busch, A. B. (2023). Telemental health for clinical assessment and treatment. BMJ (Clinical research ed.) 380, e072398. doi: 10.1136/bmj-2022-072398 | Unrelated |
|  | Menon, P. R. (2020). Pediatric Coronavirus Disease-19 (COVID-19): Meta-analyzing Literature Versus Natural History. Indian pediatrics 57, 869-870. | Unrelated |
|  | Vijenthira, A., Gong, I., Fox, T. A., Booth, S., Cook, G., Fattizzo, B., Martin Moro, F., Razanamahery, J., Riches, J., Zwicker, J. I., Patell, R., Vekemans, M. C., Scarfo, L., Chatzikonstantinou, T., Yildiz, H., Lattenist, R., Mantzaris, I., Wood, W., and Hicks, L. K. (2020). Outcomes of Adult and Pediatric Patients with Hematologic Malignancies and COVID-19: A Systematic Review and Meta-Analysis of 1847 Patients. Blood 136, 39-41. doi: 10.1182/blood-2020-141235 | Unrelated |
|  | Vijenthira, A., Gong, I. Y., Fox, T. A., Booth, S., Cook, G., Fattizzo, B., Martin Moro, F., Razanamahery, J., Riches, J. C., Zwicker, J. I., Patell, R., Vekemans, M. C. M., Scarfo, L., Chatzikonstantinou, T., Yildiz, H., Lattenist, R., Mantzaris, I., Wood, W. A., and Hicks, L. K. (2020). Outcomes of patients with hematologic malignancies and COVID-19: A systematic review and meta-analysis of 3377 patients. Blood. doi: 10.1182/blood.2020008824 | Unrelated |
|  | Cupul-Uicab, L. A., Hernandez-Mariano, J. A., Vazquez-Salas, A., Leyva-Lopez, A., Barrientos-Gutierrez, T., and Villalobos Hernandez, A. (2021). Salud publica de Mexico 63, 242-252. doi: 10.21149/11810 | Unrelated |
|  | Di Toro, F., Gjoka, M., Di Lorenzo, G., De Santo, D., De Seta, F., Maso, G., Risso, F. M., Romano, F., Wiesenfeld, U., Levi-D'Ancona, R., Ronfani, L., and Ricci, G. (2021). Impact of COVID-19 on maternal and neonatal outcomes: a systematic review and meta-analysis. Clinical microbiology and infection: the official publication of the European Society of Clinical Microbiology and Infectious Diseases 27, 36-46. doi: 10.1016/j.cmi.2020.10.007 | Unrelated |
|  | Wei, S. Q., Bilodeau-Bertrand, M., Liu, S., and Auger, N. (2021). Incidence de la COVID-19 sur les issues de grossesse: examen systematique et meta-analyse. 193, E813-E822. doi: 10.1503/cmaj.202604-f | Unrelated |
|  | Alfayez, O. M., Aldmasi, K. S., Alruwais, N. H., Bin Awad, N. M., Al Yami, M. S., Almohammed, O. A., and Almutairi, A. R. (2022). Incidence of Diabetic Ketoacidosis Among Pediatrics With Type 1 Diabetes Prior to and During COVID-19 Pandemic: A Meta-Analysis of Observational Studies. Frontiers in endocrinology 13, 856958. doi: 10.3389/fendo.2022.856958 | Unrelated |
|  | Zheng C, Shao W, Chen X, Zhang B, Wang G, Zhang W. Real-world effectiveness of COVID-19 vaccines: a literature review and meta-analysis. Int J Infect Dis. 2022 Jan;114:252-260. doi: 10.1016/j.ijid.2021.11.009. Epub 2021 Nov 17. PMID:34800687; PMCID: PMC8595975. | Unrelated |
|  | Feikin DR, Higdon MM, Abu-Raddad LJ, Andrews N, Araos R, Goldberg Y, GroomeMJ, Huppert A, O'Brien KL, Smith PG, Wilder-Smith A, Zeger S, Deloria Knoll M,Patel MK. Duration of effectiveness of vaccines against SARS-CoV-2 infection andCOVID-19 disease: results of a systematic review and meta-regression. Lancet.2022 Mar 5;399(10328):924-944. doi: 10.1016/S0140-6736(22)00152-0. Epub 2022 Feb23. Erratum in: Lancet. 2022 Apr 4;: Erratum in: Lancet. 2023 Feb25;401(10377):644. PMID: 35202601; PMCID: PMC8863502. | Unrelated |
|  | Watanabe A, Iwagami M, Yasuhara J, Takagi H, Kuno T. Protective effect ofCOVID-19 vaccination against long COVID syndrome: A systematic review and meta-analysis. Vaccine. 2023 Mar 10;41(11):1783-1790. doi:10.1016/j.vaccine.2023.02.008. Epub 2023 Feb 8. PMID: 36774332; PMCID:PMC9905096. | Unrelated |
|  | Toubasi AA, AbuAnzeh RB, Tawileh HBA, Aldebei RH, Alryalat SAS. A meta-analysis: The mortality and severity of COVID-19 among patients with mentaldisorders. Psychiatry Res. 2021 May;299:113856. doi:10.1016/j.psychres.2021.113856. Epub 2021 Mar 3. PMID: 33740483; PMCID:PMC7927594. | Unrelated |
|  | Tabatabaeizadeh SA. Zinc supplementation and COVID-19 mortality: a meta-analysis. Eur J Med Res. 2022 May 23;27(1):70. doi: 10.1186/s40001-022-00694-z.PMID: 35599332; PMCID: PMC9125011. | Unrelated |
|  | Gao P, Liu J, Liu M. Effect of COVID-19 Vaccines on Reducing the Risk of LongCOVID in the Real World: A Systematic Review and Meta-Analysis. Int J EnvironRes Public Health. 2022 Sep 29;19(19):12422. doi: 10.3390/ijerph191912422. PMID:36231717; PMCID: PMC9566528. | Unrelated |
|  | Coomes EA, Haghbayan H. Interleukin-6 in Covid-19: A systematic review andmeta-analysis. Rev Med Virol. 2020 Nov;30(6):1-9. doi: 10.1002/rmv.2141. Epub2020 Aug 26. PMID: 32845568; PMCID: PMC7460877. | Unrelated |
|  | Alimohamadi Y, Tola HH, Abbasi-Ghahramanloo A, Janani M, Sepandi M. Casefatality rate of COVID-19: a systematic review and meta-analysis. J Prev MedHyg. 2021 Jul 30;62(2):E311-E320. doi: 10.15167/2421-4248/jpmh2021.62.2.1627.PMID: 34604571; PMCID: PMC8451339. | Unrelated |
|  | Healey Q, Sheikh A, Daines L, Vasileiou E. Symptoms and signs of long COVID:A rapid review and meta-analysis. J Glob Health. 2022 May 21;12:05014. doi:10.7189/jogh.12.05014. PMID: 35596571; PMCID: PMC9125197. | Unrelated |
|  | Di Toro F, Gjoka M, Di Lorenzo G, De Santo D, De Seta F, Maso G, Risso FM,Romano F, Wiesenfeld U, Levi-D'Ancona R, Ronfani L, Ricci G. Impact of COVID-19on maternal and neonatal outcomes: a systematic review and meta-analysis. ClinMicrobiol Infect. 2021 Jan;27(1):36-46. doi: 10.1016/j.cmi.2020.10.007. Epub2020 Nov 2. PMID: 33148440; PMCID: PMC7605748. | Unrelated |
