# Supplementary material for: Evidence linking COVID-19 and the health/well-being of children and adolescents: an umbrella review
Source: BMC Med. 2024 Mar 13;22:116. doi: 10.1186/s12916-024-03334-x (PMC10938697; doi:10.1186/s12916-024-03334-x)
Supplement: Supplementary file 8 — Additional file 8. Additional COVID-19-related evidence from single-armed meta-analyses. [file 12916_2024_3334_MOESM8_ESM.docx]

**Additional file 8: Additional COVID-19-related evidence from single-armed meta-analyses**

**COVID-19-associated multisystem inflammatory syndrome (MIS-C).** For clinical manifestations, the commonest symptoms were fever, conjunctivitis and rash (**Additional file 9**), with a prevalence of 90.85% (95% CI: 89.86–91.84%), 54.9% (95% CI: 43.2–66.7%), and 49.63% (95% CI: 47.8–51.47%), respectively. The most prominent symptoms were organ damage or impairments of liver, kidney, and cardiovascular system. The prevalence of myocardial dysfunction, myocarditis, coronary vessel abnormalities, gastrointestinal symptoms, liver function damage, and acute kidney failure were 52.2% (95% CI: 48.4–56.01%), 29.34% (95% CI:27.66–31.02%),17.83% (95% CI: 13.29–22.87%), 51.98% (95% CI: 50.13–53.83%),46.29% (95% CI: 32.78–60.08%), 20.0% (95% CI: 14.0–28.0%), and 89.7% (95% CI: 79.1–96.7%), respectively. For laboratory findings, CRP, IL-6, FIB, and PCT increased significantly, with incidences of 93.22% (95% CI: 92.26–94.17%), 89.3% (95% CI: 75.3–97.88%), 87.01% (95% CI: 73.97–95.98%), and 85.1% (95% CI: 75.65–92.55%), respectively. However, it is worth mentioning that the prevelance of platelet reduction was 26.42% (95% CI: 18.19–35.58%). In addition, severe outcomes were more common in pediatric patients with MIC, exhibiting a 56.0% (95% CI: 48.5–62.0%) shock rate, a 64.81% (95% CI: 63.15–66.48%) ICU admission rate, and a 4.0% (95% CI: 1.0–14.0%) mortality rate.

**Newborns from COVID-19-diagnosed mothers.** In neonates born to infected mothers, we observed that 80.4% (95% CI: 67.1–93.7%) did not exhibit any clinical abnormalities (**Additional file 10**). Among symptomatic neonates, dyspnea, vomiting, and moaning were the most commonly reported clinical features, with prevalences of 40.1% (95% CI: 18.3–61.9%), 38.1% (95% CI: 13.8–61.2%), and 37.5% (95% CI: 17.5–84.5%), respectively. Among neonates who underwent COVID19 testing, 12.8% (95% CI: 6.6–19.0%) tested positive. Regarding fetal complications, the prevalence of preterm birth was 27.1% (95% CI: 16.3–37.8%). Furthermore, while 81.8% (95% CI: 72.9–90.8%) of newborns were discharged from the hospital, 57.7% (95% CI: 29.1–86.3%) required admission to the neonatal intensive care unit (NICU) for immediate isolation or intensive care treatment.

**Long-COVID.** The prevalence of long-COVID (as defined by the presence of one or more symptoms more than 4 weeks following a COVID19 infection) in children and adolescents was 25.24% (95% CI: 67.1–93.7%) (**Additional file 11**). The five most prevalent clinical manifestations were fatigue (17.36%, 95% CI: 11.83–23.69), mood symptoms (e.g., sadness, tension, anger, depression, and anxiety) (17.36%, 95% CI: 11.83–23.69), dyspnea (17.36%, 95% CI: 11.83–23.69), sore throat (17.36%, 95% CI: 11.83–23.69), and sleep disorders (17.36%, 95% CI: 11.83–23.69). In addition, after 3 months of infection, abnormal pulmonary function was reported as 24% (95% CI: 4–43%), and exercise limitations were identified with a prevalence of 48% (95% CI: 25.0–70.0%).

**Events caused by the COVID-19 vaccine.** Local adverse events (AEs) such as pain, swelling, erythema, and redness, as well as systemic AEs like fatigue, headache, and myalgia, were the most commonly reported adverse reactions to vaccines (with the corresponding incidence rates detailed in **Additional file 12**). The frequency of both local and systemic AEs was highest after the booster dose (25.64%, 95% CI: 2.08–63.01; 28.75%, 95% CI: 15.3–44.47%), followed by the second dose (23.93%, 95% CI: 10.52–40.73; 24.67%, 95% CI: 19.51–30.23%) and the first dose (17.6%, 95% CI: 6.17–33.27; 13.52%, 95% CI: 10.02–17.45%). Serious AEs, including carditis (0.00376%, 95% CI: 0.002357–0.005919%) and severe allergic reactions (0.64%, 95% CI: 0.37–0.99%), were less frequently reported among vaccinated adolescents when all doses were considered. Among the health impacts of vaccines, antipyretic use was frequently reported at 43.93% (95% CI: 30.52–57.81), while inability to perform daily activities (17.36%, 95% CI: 11.83–23.69) or attend school or work (6.96%, 95% CI: 3.75–11.08) were reported less frequently.

**Health impacts during the pandemic.** The COVID-19 pandemic has had a profound impact on mental health, sleep patterns, and physical activity among children and adolescents. Symptoms such as depression, anxiety, and inattention have become increasingly prevalent in this age group. The rates of depressive symptoms, anxiety symptoms, and sleep disturbances were found to be 31.0% (95% CI: 27.0–35.0%), 31.0% (95% CI: 27.0–35.0%), and 49.0% (95% CI: 39.0–58.0%) respectively (**Additional file 13**). Additionally, the percentage of youths participating in physical activity before the pandemic was 46.4% (95% CI: 35.5–57.7%), which decreased to 19.5% (95% CI: 15.8–23.8%) and 40.6% (95% CI: 24.3–59.2%) during the pandemic, with and without restrictions, respectively.
